# Modelling successful primary care for multimorbidity: a realist synthesis of successes and failures in concurrent learning and healthcare delivery

**DOI:** 10.1186/s12875-015-0234-9

**Published:** 2015-02-25

**Authors:** Sarah Yardley, Elizabeth Cottrell, Eliot Rees, Joanne Protheroe

**Affiliations:** Primary Care and Health Sciences, Keele University, Keele, Staffs, ST5 5BG UK

**Keywords:** General practice, Health service delivery, Medical education, Multimorbidity, Primary care, Realist synthesis, Socio-cultural theories

## Abstract

**Background:**

People are increasingly living for longer with multimorbidity. Medical education and healthcare delivery must be re-orientated to meet the societal and individual patient needs that multimorbidity confers. The impact of multimorbidity on the educational needs of doctors is little understood. There has been little critique of how learning alongside healthcare provision is negotiated by patients, general practitioners and trainee doctors. This study asked ‘what is known about how and why concurrent healthcare delivery and professional experiential learning interact to generate outcomes, valued by patients, general practitioners and trainees, for patients with multimorbidity in primary care?’

**Methods:**

This realist synthesis is reported using RAMESES standards. Relationship-centred negotiation of needs-based learning and care was the primary outcome of interest. Healthcare, social science and educational literature were sought as evidence. Data extraction focused on context, mechanism and outcome configurations within studies and on data which might assist understanding and explain; i) these configurations; ii) the relationships between them and; iii) their role and place in evolving programme theories arising from data synthesis. Mind-mapping software and team meetings were used to aid interpretative analysis.

**Results:**

The final synthesis included 141 papers of which 34 contained models for workplace-based experiential learning and/or patient care. Models of experiential learning for practitioners and for patient engagement were congruent, frequently referencing theories of transformation and socio-cultural processes as mechanisms for improving clinical care. Key issues included the perceived impossibility of reconciling personalised concepts of success with measurability of clinical markers or adherence to guidelines, and the need for greater recognition of social dynamics between patients, GPs and trainees including the complexities of shared responsibilities. A model for considering the implications of concurrency for learning and healthcare delivery in the context of multimorbidity in primary care is proposed and supporting evidence is presented.

**Conclusions:**

This study is novel in considering empirical evidence from patients, GPs *and* trainees engaged in concurrent learning and healthcare delivery. The findings should inform future interventions designed to produce a medical workforce equipped to provide multimorbidity care.

**Trial registration:**

PROSPERO International prospective register of systematic reviews CRD42013003862

**Electronic supplementary material:**

The online version of this article (doi:10.1186/s12875-015-0234-9) contains supplementary material, which is available to authorized users.

## Background

Demographics of health and illness are changing; people are increasingly living for longer with multimorbidity, defined as the ‘co-existence of two or more conditions, where one is not necessarily more central’ [1]. The rising prevalence of multimorbidity creates new challenges for patients and professionals from the necessity of living with and managing long term conditions. The co-existence of multiple non-curative problems within individuals requires complex management which is poorly supported by current clinical guidelines. A significant challenge to contemporary medical practice is the need to promote positive and holistic approaches to patient care in a range of situations where ‘cure’ is not an option [1].

There is a gap in understanding of how the education of future professionals might address the consequences of this deficit [2]. Medical practice needs to be re-orientated to meet the societal and individual patient needs conferred by multimorbidity. Traditionally, healthcare and medical education has been delivered using index condition models, i.e. those focused primarily on a single disease with the inherent assumption this is the sole or pre-eminent clinical problem, and often defined in purely biomedical terms. Such models inadequately equip a medical workforce to collaborate with patients to address multimorbidity when the sum of multiple morbidities can have effects greater than the constituent parts [3]. Although the construct of doctors as healers, with cure the ultimate marker of success, remains a strong societal narrative, it is problematic in the context of multimorbidity arising from progressive incurable diseases.

The impact of this on educational needs of trainee doctors (‘trainees’) and general practitioners (GPs) is little understood. There is also a lack of critique regarding how concurrent healthcare provision and experiential learning (in workplaces) is negotiated by patients, GPs and trainees. Medical education relies heavily on experiential learning. Therefore learning and healthcare delivery are concurrent in clinical workplaces. Professionals in primary care training sites must reconcile the goals of providing individualised health care with the provision of constructive workplace-based learning for future professionals. Models derived from both theoretical and empirical research for the ‘ideal’ delivery of care and the ‘ideal’ delivery of education have tended to ignore the fact that both occur in the same place, at the same time, involving the same people, and both are affected by how people think, feel and act in relation to each other and the circumstances in which they find themselves. Seeking to understand patient, GP and trainee definitions of success and failure in the absence of cure is key for developing an understanding about how and why social interactions lead to different learning and care delivery outcomes in multimorbidity.

Credible learning solutions in the context of multimorbidity must account for inherent uncertainty and unpredictability. Constructive learning from experience requires a willingness to change and be transformed as new experiences are assimilated and accommodated to refine existing knowledge, skills and behaviours [4]. Personal and professional experiences of multimorbidity may produce transformative learning when new ways to make sense of, and create meaning from, an uncertain and evolving experience of chronic conditions are sought. It is, therefore, desirable that transformative learning is encouraged among patients and professionals as both a coping mechanism and to prevent potential iatrogenic harms arising from the uncritical application of scientifically possible, but not necessarily beneficial, medical interventions. An example of this is blanket application of clinical guidance for preventative medical interventions despite lack of evidence to demonstrate benefits outweigh risks in all patient populations. To make a judgment about the burden of hospitalisation in the event of an acute deterioration without recourse to the individual patient’s priorities and values, as well as the likelihood of benefit for that particular individual is an example of how professionals can fail to learn in ways that integrate evidence with experiential expertise.

The overarching research question driving this realist synthesis was ‘What is known about how and why concurrent healthcare delivery and professional experiential learning interact to generate outcomes, valued by patients, GPs and trainees, for patients with multimorbidity in primary care?’

Specifically as the synthesis developed we sought to:

1. Describe what is known about relationship-centred negotiation of needs-based learning^a^ and needs-based care^b^ with a focus on models of patient care and/or workplace learning;
2. Understand the mechanisms at play when healthcare delivery and workplace-based education concurrently occur, and;
3. Synthesise conceptualisations of success and failure in the absence of cure, identifying how this affects learner and patient outcomes in the context of multimorbidity in primary care.

To make healthcare service provision and education appropriate for multimorbidity management requires an understanding of patients’, GPs’ and trainees’ definitions of success and failure when cure of illness is not an option. Such management needs to include relationship-based negotiation of needs-based learning (for patients and professionals including trainees) and needs-based care (for patients, delivered by professionals including trainees), and so this formed our primary outcome of interest. The elements of this primary outcome are chosen deliberately. It is not possible for a GP who is both delivering patient care and providing experiential learning to trainees to simultaneously and concurrently be 100 percent patient centred *and* 100 percent learner centred. We use the term ‘needs-based’ for both learning and care because an underpinning tenet of the theoretical orientation of this work is that learning should be based on the need of the learner, just as care should be available according to the need of the patient. Negotiation between patients, GPs and trainees has to occur to ensure different, potentially competing, needs are held in balance. This negotiation is dependent on relationships. These relationships and the social interactions arising from them will lead to contextually based understandings of success and failure.

Given that healthcare is inherently dependent on human action, it is surprising that little attention has been given to understanding how and why human action may strengthen or impede mechanisms for needs-based healthcare delivery and experiential learning to make this sustainable in the context of multimorbidity. The study commenced from the premise that socio-cultural theories could be used to identify and understand mechanisms at play when healthcare delivery is concurrent with workplace-based experiential learning. In our synthesis Vygotskian derived socio-cultural theories informed our initial theories of how social mechanisms might function in the context of multimorbidity. These theories consider the social context in which people create meaning, construct knowledge and generate learning from experiences. The theoretical orientation of the synthesis was directed to considering both the individual and the collective learning that might arise from interactions between patients, GPs and trainees with regard to conceptualisations of success or failure in the context of multimorbidity and the absence of cure. Use of these theories also directed our data extraction to consider unintended as well as intended consequences of human interactions. The protocol [3] contains further details regarding the theoretical and methodological orientation, and justification of the choices made during study design.

In this paper we provide a summary report of the realist synthesis considering two main findings: (i) issues of concurrency in learning and healthcare delivery and (ii) conceptualisations of success (and failure) in the absence of cure. With this focus we present:

1. A descriptive summary of the synthesis data focusing on the two main findings;
2. Novel findings and emergent programme theories^c^ arising from the synthesis;
3. A new model of specific programme elements^d^ most likely to achieve desired outcomes for patients and trainees.

Initial findings from the review identified a paucity of work addressing the interactivity between models of experiential learning and models of patient care, despite the similarities regarding socio-cultural processes in each. This observation, combined with the identification of evidence which could be used to build understanding of how people define success and failure in multimorbidity when there is an absence of cure, led us to focus our review on non-linear (i.e. not sequential or straightforward) transitions that are mediated through social interactions.

A full study report prepared for the purposes of capturing the entire history including a complete record of the methodological and analytical documents generated during the course of the study on which this paper is based has been made freely available as an Additional file 1. It contains expanded methodological commentary, including how trainees were engaged with the project, evolution of the search strategy, further information on judgements regarding data extraction and data synthesis plus summaries of all studies containing models, all other empirical studies included in the synthesis and tabulated data identified in the synthesis as markers of success and failure (i.e. our raw data extractions). A second paper is in submission in which we provide an interpretative synthesis comparing patients’, GPs’ and trainees’ lived experiences of multimorbidity following a secondary analysis of qualitative data identified in the course of this realist work.

## Methods

This is a realist synthesis reported using RAMESES standards [5]. The review team included patients, medical students, a postgraduate trainee, and clinical academics (including GPs). A wider pool of GPs provided feedback on the emerging findings.

For this study, with respect to multimorbidity it was decided that relevant conditions must: be distinct clinical entities rather than one condition being an extension or direct complication of another, cause patients to experience troublesome symptoms, and currently have no definitive cure (at least for the vast majority of patients). We have not limited our interest to any specific stage of condition trajectories [3]. Primary care encompasses care led by GPs in the UK or the nearest equivalent elsewhere [3]. Health service delivery refers to any care provided to individuals or groups of patients by qualified health professionals and the associated structures and institutions through which this care is organised [3]. The term ‘experiential learning’ is used in this paper to describe any learning that arises from workplace-based interactions, that is, the creation of meaning or construction of knowledge from ‘real life’ experience [6]. The term ‘trainees’ is used to refer to both undergraduate students and postgraduate trainees.

### Design

Realist synthesis was the most appropriate method for exploring concurrency between two complex social interventions occurring within clinical workplaces (healthcare delivery and experiential learning) because it allows an attempt to identify transferable recommendations on which to base educational innovation and improvement [7]. An outline of the processes of the study is given in Table 1 with further details and justifications for modification to the previously published design [3] are presented below.

**Table 1:** **Steps in the realist synthesis – a summary of the study approach**

**Step**	**Summary of approach**
1 Focusing the review	The social interactions between GPs, patients and trainees where chosen as a focus within the context of multimorbidity in primary care because (i) multimorbidity is an increasing clinical and educational challenge, (ii) workplace-based learning has to occur concurrently with healthcare delivery to ensure future doctors are equipped to meet the needs of patients with multimorbidity
2 Developing a theory:	(a) Initial rough theory – we theorised that as social interactions are known to shape learning (meaning-making and knowledge construction) it was likely that social mechanisms influenced concepts of success and failure in the absence of cure and hence understanding this was essential to understanding mechanisms that would lead to relationship-centred needs-based learning and care delivery
	(b) Review of evidence – an extensive systematically conducted database search with citation follow-up was conducted as described in this paper and our protocol.
	(c) Refined theory – the model presented below represents the mechanisms which, if triggered, are most likely to lead to constructive transformations and learning for GPs, patients and trainees
3 Search strategies:	These are detailed fully in the protocol, with the Medline search provided in additional file 2 as an exemplar.
4 Selection and appraisal of documents	As described in the main text citations were selected according to relevance and rigour.
5 Applying realist principles in analysis	The data extraction sheet provided a framework for ensure that data was pulled from each citation to inform understanding of social interactions, complexity, concurrency, success or failure in multimorbidity, learning and service provision (version 1 and 2 – a more focused version for later rounds of data extraction can be found in additional files 4 & 5). The use of this framework which incorporated the elements of the VICTORE model [8] as well as members of the study team being required to keep note of how each citation informed ‘what works , for whom, in what circumstances and why?’ with respect to the study aims ensured that data extraction included the seeking of explanations (how in a given context did a mechanism generate an outcome), comparison of interventions, aligning evidence to theory taking a bi-directional approach (allowing the evidence to refine our theory as well as theoretically informed searching for evidence) and iterative development of the proposed model

Further details on the design of the review can be found in our published protocol.

### Completion of study processes and modifications

1. Final search strategy
  Healthcare, social science and educational literature were sought as evidence. Multimorbidity is not currently a MeSH term. Therefore, in addition to searching for ‘multi-morbidity’ and ‘multimorbidity’ as keywords, MeSH terms for chronic disease and comorbidity were used.
  In the final strategy four search threads were combined: (multimorbidity) AND (primary care) AND ((education) OR (workplace experiences)). No date, language, design or other limitations were used. Following comprehensive searching in 20 databases (see Table 2) citation searches were conducted alongside review of relevant grey literature. The complete strategy is available in Additional file 2. All identified citations were screened.

**Table 2:** **Database list**

**Database list**		
•Academic Search Complete	•Cochrane	•Opengrey
•Applied Social Sciences Index and Abstracts	•Embase	•PsychInfo
	•Education Resources Information Center	•Science Direct
		•Social Services Abstracts
•Australian Education Index	•Health Management Information Consortium	•Sociological abstracts
•British Education Index	•Joanna Brigg Institute	•Web of Science
•Best Evidence Medical Education	•Kellogg Foundation	
•British Nursing Index	•Medline	
•CINAHL
  Table 2 Databases included in searches (initially conducted on 1^st^ Aug 2012, and updated via alert systems until 1^st^ Aug 2013).
  Articles were retained for review if they were about chronic disease, for example, chronic disease models, as likely to include multimorbidity, and if they pertained to primary care or similar settings (i.e. those in which the same mechanisms may be in operation). Articles were excluded through consensus among the research team if these did not describe healthcare delivery, medical education or social processes or if specifically focussed on single diseases.
2. Iterative searching
  After completing the protocol, there was a paucity of data regarding models of best practice for experiential learning. Therefore two iterative strategies were implemented: (i) additional searching to extend the ‘educational’ search thread and (ii) re-screening of citations initially excluded due to them not being set in primary care to identify data relating to concurrency of education and healthcare delivery which might be transferable to primary care settings.
  Additional searching included cross-checking against previous review work [9-11] and purposively selecting experiential learning literature with reference to medical trainees. This led to the inclusion of the ‘experience based learning’ (ExBL) model [11] and Werne *et al*’s work [10]. Search terms from this work, not used in our previous searches, but with potential relevance to our objectives, were: ambulatory care, role model, supervisor and supervisee. A renewed search was performed in Medline using these terms to detect further data. A further 10 citations were added to the synthesis following title screening of the search results. Inclusion and exclusion criteria and judgements about quality were applied to whole papers identified as data as described in our protocol [3].

### Critical appraisal

At least two members of the study team critically appraised each citation for relevance to the research questions and synthesis objectives (i.e. richness, defined as the citation offering data that could contribute to theory building), in addition to completing an assessment of rigour (defined as an appraisal of whether the citation included methods appropriate to generate credible and trustworthy data). Given that the citations contained a range of methods, and our focus on social interactions we did not employ a single set of appraisal criteria but instead judged each citation according to the methodological standards of its genre. All citations from which data was used are listed in Additional file 3: citation list.

### Data extraction and synthesis

The whole text of citations was viewed as ‘raw data’ for this synthesis. Because the outcome of an intervention depends on the nature of the intervention itself and the context it occurs in, in realist methodology one must seek to understand the ‘CMO’ (‘context^e^, mechanism^f^(s) and outcomes^g^ (s)’) in order to make sense of what and how any intervention works and in whom. Traditionally ‘an intervention’ may be thought of as a ‘program’ that can be introduced to seek a particular outcome. In the context of this work the definition of an intervention was necessarily looser. Simply having patients with multimorbidity, GPs and trainees in primary care in each others’ presence means *something will happen* as a *natural experiment* occurs. Empirical data reporting such interventions was included in the synthesis as well as that from pre-designed and controlled interventions.

A data extraction sheet (see Additional files 4 and 5) was designed to focus data extraction on ‘CMO’ (context, mechanisms and outcomes) configurations within studies and on data which might assist understanding and explain; i) the CMO configurations themselves; ii) the relationships between them and; iii) their role and place in evolving programme theories. Social interactions are inherently unpredictable and complex, therefore the realist view on complexity, captured in the VICTORE model [8] was used to guide data extraction. VICTORE identifies seven component parts to complexity: volitions (i.e. the choices and actions of people), implementation (the realities of any intervention including unintended consequences), contexts, time, outcomes, rivalry (between competing ideas, priorities etc.) and emergence (a holistic view of what takes shape once a change sets various mechanisms in motion). Considering these components from a socio-cultural theoretical orientation was essential to understanding how patients, GPs and trainees interacted with and influenced each other in their understanding of multimorbidity. We used the seven components to sensitise us to these issues in our data extraction, making each a ‘code’ to which data could be allocated when it informed understanding of that particular aspect of complexity within primary care. With each round of data extraction, modifications were made to the data extraction sheet to sharpen the focus on the most useful and/or outstanding elements of the review.

**Figure 1:**
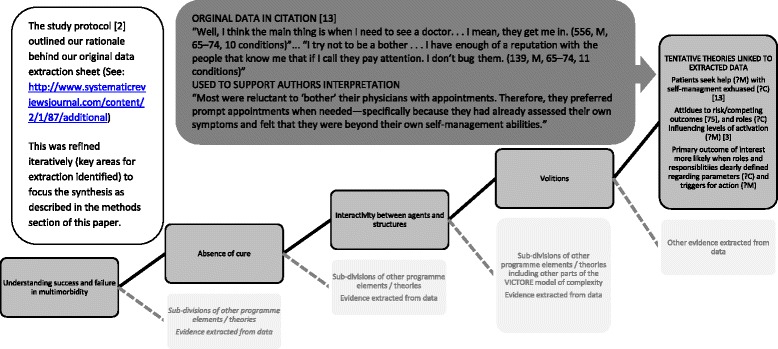
**In the dark grey text box are verbatim extracts from a citation used as data.** These point to a summary of what was extracted from this citation (mid-grey box on right-hand side). In this summary possible context (?C) and mechanisms (?M) are noted. During data extraction this evidence was linked to the concept of volitions, which as we gradually developed a mind-map of the data and its interpretation was linked in turn to ‘interactivity’ in the context of ‘absence of cure’ as we sought to understand success and failure within our synthesis. The line of mid-grey boxes demonstrating these links can be read from left to right or vice versa, mirroring the iterative process between theories and data extraction during the synthesis. Pale grey boxes provide a representation of the rest of the mind-map as it is too detailed to show in its entirety. Further details of the full data extraction and synthesis process are given in the methods section of this paper and text Table 1.

Data synthesis was led by SY although all members of the review team actively participated. During the synthesis the team engaged in iterative selection and testing of candidate theories and actively sought data to refute or refine these. This process led to the selection of transformative learning as a theory to pursue, when it emerged that this held commonality across patient and trainee trajectories. Mind-mapping software and team meetings were used to develop an interpretative analysis by progressive assimilation and creation of tentative links between different data sources (See Figure 1 for a worked example of this).

As described above transformative learning was identified as a theory that appeared to align with the commonalities within the interactions for the three groups. Having identified this we discussed further the citations in which relevant theoretical or empirical models for learning or patient care were presented. Common ideas and theories within these citations were used to develop an integrated interpretation of transformative learning in the form of a new programme theory (expressed as a model, see below) to describe current data on how and why conceptualisations of success and failure impact on non-linear transitions (i.e. transformations) for patients, GPs and trainees. This model was then finessed using confirmatory/conflicting evidence from other data extractions.

As the synthesis progressed the initial focus (models of patient care and/or workplace learning) within our primary outcome of interest (relationship-based negotiation of needs-based learning and needs-based care) was refined to consider more specifically non-linear transitions in multimorbidity for patients, GPs and trainees. Re-focusing was necessary as it emerged that transitions were an important process for all three groups, with implications common to their interactions. Patients, GPs and trainees learn and adapt to their situation through cycles of change, sense-making, learning and adaptation. A change in context triggers this situation off again. These transitions were identified as a recurring theme in the data which also provided evidence that transitions are mediated through social interactions and are influenced by levels of belief or non-belief in the potential for change or transformation. Therefore data was specifically sought to illuminate how transitions are mediated through social interactions and influenced by levels of belief or non-belief in the potential for change or transformation. In the context of multimorbidity the data extracted for this synthesis supports the idea that ongoing transitions for all three groups are mediated through social interactions and influenced by levels of belief or non-belief in the potential for change or transformation.

Unless otherwise stated, results from this synthesis are reported as interpretations of data found in the included studies, rather than simply what others said in their study. References are, however given to allow tracing to the key sources of data informing each interpretation, or where we have adopted a specific term from the included data.

## Results

In this section we first present a descriptive summary of the synthesis data focusing on the two main findings (i) issues of concurrency in learning and healthcare delivery and (ii) conceptualisations of success (and failure) in the absence of cure. We then describe novel findings and emergent programme theories arising from the synthesis before describing an explanatory model that interprets the synthesis data into a new emergent theory of how social interactions can function as mechanisms to trigger constructive transformative learning for patients, GPs and trainees with respect to multimorbidity in primary care.

1. A descriptive summary of the synthesis data

**Figure 2:**
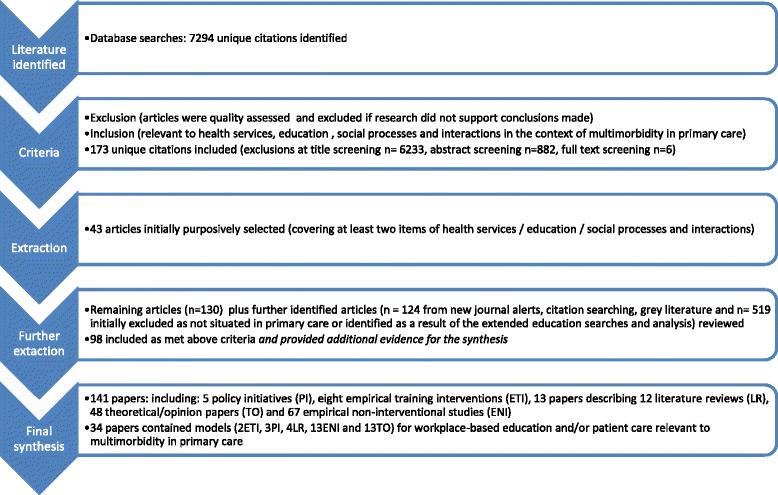
**Document flow.**

On completion the final synthesis included 141 papers of which 34 papers contained models for workplace-based experiential learning and/or patient care (see Figure 2). As described above this was the result of an iterative process during which concurrent data extraction and theory building were undertaken by the study team. Sixteen citations contained empirical or model-based studies that were specific to multimorbidity in primary care. Brief summaries of the methods and key findings of each of these citations are provided in Table 3 in order to illustrate the current range of proposed interventions relevant to our research question. None contained purposely designed interventions for improving learning *and* healthcare delivery. Thirteen were empirical non-interventional studies (ENI) (two of which produced explanatory models for healthcare delivery and interactions). Of the remaining three, all produced models; one was a literature review (LR) and two were theoretical papers (TO).

**Table 3:** **Empirical and model based studies specific to multimorbidity in primary care**

**Study**	**Methods**	**Key findngs**
Loffler [12]	ENI with model: grounded theory analysis of patient interviews producing a model of coping categories, strategies and outcomes for patients	•Multifaceted coping strategies among patients (aged 65–85) with multimorbidity
		•Patients distinguished between emotional coping (when it is believed nothing can be done to change the situation) and problem-solving focuses of coping which they used when they had expectation of change (social and practical coping)
		•Patients keen to preserve their autonomy but described emotional oscillation between anxiety and strength
		•Many of them were making reasoned choices about their use of medication, even when this conflicted with professional advice
Morris [13]	ENI with model: longitudinal semi-structured interviews with patients	•Theoretical model produced identifying four factors which influenced self-management
		1.disruption by conditions (lack of engagement, confusion, being overwhelmed, uncertainty, separation of conditions)
		2.accommodation of conditions (continuity from existing illness behaviour/integration with existing practices, control over conditions and symptoms, enough understanding of conditions, confidence)
		3.factors influencing the shift from accommodation to disruption (exacerbations, confusion and contradictory information, events, loss of control, medication)
		4.factors influencing shift from disruption to accommodation (taking control, links between existing knowledge and experiences, adapting information and practices into new routines, interaction with health care professionals)
		•Patients sought to make new diagnoses minimally disruptive and may have benefited from discussion of their priorities with professionals and/or better information on which to prioritise
Barnett [2]	ENI: cross-sectional epidemiological study	•Demonstrated that over 40% of patients in large Scottish sample had one or more chronic disease and over 20% had multimorbidity, defined as two or more chronic conditions
		•Multimorbidity increased with age (although the absolute number of people was higher under 65 years) and with social deprivation
		•Mental health disorders were a significant feature
Bower [14]	ENI: qualitative interviews with GPs and practice nurses	•Identified tensions primary care professionals experience between delivering care to meet externally imposed targets and achieving patients’ personal agendas
		•Amongst interviewees there was limited consideration of interactions or synergies between conditions and their management
Fortin [15]	ENI: psychiatric symptom questionnaire study	•Significant association with increased distress as severity of morbidities increased (although not with a simple count of number of conditions suggesting that functional impact may be relevant)
Luijks [16]	ENI: Group interviews with GPs	•Themes that were important in the practical experiences of GPs: managing multimorbidity in the face of limited scientific evidence, applying an integrated approach, medical considerations placed into perspective of patients, shared decision-making and responsibility
		•Outworking of themes influenced by the personal relationship between doctor and patient, whether the patient had mental health problems, interacting conditions and practical problems such as shortage of time and polypharmacy
Moth [17]	ENI: cross-sectional study	•Over 30% of Danish GP consultations were with patients who had more than one chronic disease and a rise in time consumption and contact burden was associated with this
		•Diagnoses of depression and dementia led to particularly complex consultations as did additional psychosocial problems
		•Few contacts were considered appropriate to delegate to other members of the primary care team by the GPs
Frueh [18]	ENI: focus groups with patients	•Identified problems of poor levels of function, negative psychological reactions, negative effects on relationships and interference with work or leisure activities
		•Polypharmacy a major concern
		•Some patients described problematic interactions with professionals and health care systems
		•Patients were willing to engage in self-management and the use of technology but did not want this to replace human contact
		•Support from professionals other than doctors was considered acceptable if complementary rather than replacing doctor consultations
Noel [19]	ENI: cross-sectional survey	•Patients with multimorbidity were significantly more likely to express willingness to learn self-management techniques than those with a single chronic condition, and a higher percentage of those with multimorbidity were willing to see non-physician providers
O’Brien [20]	ENI: qualitative study of GPs and practice nurses	•Management of multimorbidity experienced as an ‘endless struggle’ of trying to manage illness in the context of chaotic lives with few resources, personal consequences for some professionals and a desire to pursue holistic approaches
		•Authors conclude that data confirms the presence of an inverse care law in the context of multimorbidity
		•Professionals were concerned that these patients lacked the self-efficacy to pursue self-management and thought there was a need for health care delivery systems to be redesigned
Schuling [21]	ENI: qualitative focus groups with GPs	•GPs were able to delineate differences between symptomatic and preventative medication but found the latter more difficult to deprescribe with concerns about patients feeling they had given up on, conversations about life expectancy versus quality, and contradicting guidelines
Smith [22]	ENI: qualitative focus groups with GPs and pharmacists	•Problems with health systems included: lack of time, inter-professional communication difficulties and fragmentation of care
		•Personal issues for these clinicians with respect to roles, clinical uncertainty, avoidance, patient concerns and potential management solutions
Townsend [23]	ENI: patient interviews using Bourdieu’s concepts for analysis	•Broader cultural structures became part of individuals’ narratives of their illness with for example GPs perceived to be the dispenser of capital (e.g. legitimising the sick role)
		•Patients experienced losses of previously taken for granted activities, disrupted family relationships, and awareness of a sense that they were not fulfilling societal expectations
		•Many adopted strategies such as stoicism to try and regain control and avoid being judged as ‘failures’
AGS [24]	LR with model	•Model approach recommends first focusing on the each patient’s primary concern before either addressing a specific aspect of care in negotiation with the patient or reviewing the whole care plan
		•Consideration of prognosis, interactions within and among conditions and treatments, benefit and harm and regular reassessment should all form part of the negotiation
		•Model was not tested in practice.
Boyd [1]	TO with model	•Draws heavily on the ‘Chronic Care Model’ [25] with its emphasis on a patient-centred approach
Soubhi [26]	TO with model	•Theoretical model of care which draws on communities of practice theory to develop shared learning between patients, their families and professionals

Concurrency of learning and healthcare delivery, as mediated by social interactions and particularly in the context of multimorbidity, has not been well studied to date. No papers identified contained empirical evidence from patients, GPs *and* trainees engaged in concurrent learning and healthcare delivery in a single model. Only two papers considered healthcare delivery, professional education *and* social processes/interactions [27,28]. Further, most papers describing experiential learning did not focus on multimorbidity, although chronic illness care was included within the spectrum of learning. Due to these limitations in the literature, transferring models and theories required theoretically guided interpretation for which socio-cultural theories [3,29-34] were used. The value of socio-cultural theories in unmasking mechanisms at play in concurrent healthcare delivery and workplace-based experiential learning was confirmed as the analysis proceeded. The synthesis highlighted a strong recognition within the literature of the complex, socially constructed realities of practice. Socio-cultural theories provided a way to approach the analysis of these realities, considering the perspectives of patients, GPs and trainees engaged in concurrent healthcare delivery and experiential learning (i.e. both activities at the same time and in the same physical setting). Two related themes emerged that were shared between patient and trainee experiences: (i) experiential and transformative learning, and (ii) socio-cultural elements of success. These are discussed in relation to concurrency of learning and healthcare delivery, followed by conceptualisations of success and failure in multimorbidity.

### Issues of concurrency of learning and healthcare delivery with respect to multimorbidity in primary care

Regarding concurrency, there appeared to be two key issues for learning and healthcare delivery: 1) personalised concepts of success (for patients, GPs and trainees) that were incommensurable with measurability of clinical markers or adherence to guidelines [21,22,26,28,35-41], and 2) the need for greater recognition and evaluation of how role modelling and interpersonal dynamics in social interactions impact on and influence experiential learning [10,12,23,26-28,42-53]. It is important to also note that, from the clinical perspective, learning from experience is dependent on ‘readiness for change’ [45,47] as transformation requires assimilation and accommodation. Approaching this issue from an educational perspective Leykum *et al.* [48] propose a similar theory for change with their concept of engaging in ‘reciprocal learning’. People need to be willing and interested in integrating new understanding into their existing conceptualisations rather than rejecting outright new information or taking a tokenistic approach to apparently adopting behaviour without actually believing it is of value [54,55].

In two empirical interventional (EI) model studies [27,28], learning and healthcare delivery were implicitly intertwined as changes in attitude were required for both patients and GPs in order for them to learn and work as a team [27]. Professional ownership of change was identified as an essential mechanism to bring about practice developments [28]. In support of these findings Henschen *et al.* [47] found that integration of medical students into workplaces improved and patient support increased when students were actively engaged in provision of authentic healthcare delivery in chronic disease. Supporting trainees to provide meaningful care provision generated ‘knowledge in practice’ [26,48] and potentially benefited patients [50]. Successful care and learning were dependent on good interpersonal relationships based on trust.

A few specific constructs of failure were also identified, although often these were simply the negative corollaries of success constructs. The potential for misapplication of ‘safety agendas’ to produce risk avoidance (as opposed to risk management) that would be detrimental to experiential learning in the short term and patient care (as a secondary effect) in the long term [11]. A lack of readiness or willingness to change was identified as a cause of difficulties in interactions between patients and practitioners [45,46]. However, other evidence identified that even if there was a breakdown in relationships and interaction this was potentially surmountable if all involved were willing and able to reflect on and learn from the experience [22]. The importance of the doctor-patient interpersonal relationship as a mechanism to overcome challenges was identified [16,20] although these relationships could sometimes be problematic [15,18-20]. The complexity of these interactions was highlighted by Townsend [23] who identified patient perceptions that GPs were the dispensers of illness capital in situations when patients felt unable to fulfil social roles.

### Conceptualisations of success and failure with respect to multimorbidity in primary care

Much of the data was dedicated to exposing the depth and breadth of angst related to the ‘social problem(s)’ of multimorbidity [1]. Conceptualisations of success for both patients (i.e. good care) and trainees (i.e. learning to give good care) were socially constructed and dependent on positive relationships, trust and support from others including doctors [11,12,27,28,47,50,56,57]. Success in the form of high quality care was subject to flexible, personalised and changeable definitions [44,58-60] which usually went beyond clinical markers of disease or quality markers in current healthcare policy. Patients identified their proactive behaviour as key to coping with multimorbidity. Mechanisms used by patients, (i.e. their own ‘free-style’ interventions), to achieve this included maintaining a social role and/or meaningfulness, choice in the context of support when needed, achieving goals, understanding diseases and having autonomy to prioritise medication [12,13]. Patients’ individual levels of ability to engage in these mechanisms often fluctuated, mirroring their experiences of cycles of disruption – accommodation – disruption – accommodation over time as multimorbidities changed and impact on patients’ ability to function evolved [13]. Accommodation required mechanisms such as engagement with illness management and development of new learning and understanding to develop coping strategies. These findings were replicated in many of the other empirical non-interventional studies specific to multimorbidity in primary care and papers suggesting models of care indicated that improvements to healthcare delivery should include collaborative working with patients [1,2,9,24,26] and similar ideas linked to communities of practice theory [30] as a means for ongoing professional learning.

Conceptualisations of failure tended to relate to quantifiable aspects of care [1,27,28,61-63]. This may simply reflect the nature of tools currently available to assess effectiveness and efficiency of care and learning in the context of multimorbidity. However, it may be that success is expressed in qualitative terms while failure may be conceptualised according to clinical biomarkers and other quantifiable measures. Others issues highlighted as being counterproductive to collaborative working with patients, such as time constraints and targets mismatched to patient needs, or preferences, were also highlighted [14,16,17].

Examples of key constructs of success and failure in the context of multimorbidity in primary care, among patients, GPs and trainees are summarised in Table 4. Notable for its absence in the synthesis was a positive construct about clinical biomarkers. This suggests that a focus on clinical biomarkers alone is never sufficient, and may sometimes be unnecessary/inappropriate, particularly for patients. Very similar themes including the importance of collaboration, negotiation, trust and relational working can be seen in the key constructs of success for experiential learning and healthcare delivery.

**Table 4:** **Key constructs of success and failure**

	**Key constructs of success**	**Key constructs of failure**
Health care delivery	•Collaborative working practices	•Repeated/prolonged hospital admissions
	•Holistic and transparent goals developed through negotiation	•Clinician reluctance to look beyond biomedical markers
	•Integration of medical and experiential knowledge regarding diseases and impact	•Negative corollaries of the described constructs of success [1,13,24,26,37,44,57,61-65]
	•Professional sharing of best practice	
	•Transformative learning through trusted relationships between patients and practitioners to enable self-management [1,10,12,24,26-28,37,44-48,51,52,57,61,63-69]	
Experiential learning in workplaces	•Learning to engage in and benefiting from collaborative working	•Contexts which reduced students and patients to passive roles
	•Reciprocal learning: viewing learning as a shared social process	•Negative workplace cultures
	•Learning from direct interaction with patients	•Lack of exposure to multimorbidity with excessive focus on single-disease frameworks
	•A supportive environment for the appropriate mix of responsibility, challenge and scaffolding to permit a safe but legitimate role in practice	•Overreliance on guidelines often not developed on evidence applicable to patients with multimorbidity in primary care [27,28,50,65]
	•Physical space to allow interactions between patients and trainees	
	•Patients and practitioners needed to learn how to make personalised trade-offs between risks and benefits in multimorbidity and to manage competing priorities which could change over time [10-12,26,27,47,48,50-53,56-59,64,68-75]	

### Novel findings and emergent programme theories arising from the realist synthesis

Within the synthesis a number of proposed models for learning, care delivery or both were identified. None of the citations contained full implementation studies of these models but important aspects of implementation and intended outcomes within these studies could be identified. A summary of the analysis of these models is provided in Table 5, which demonstrates that more is known about the intended outcomes of proposed models and interventions than about what happens in reality. There was some limited evidence of naturally occurring mechanisms and contextual constraints which could lead to undesirable outcomes for both patients (e.g. dissatisfaction with care offered) and trainees (e.g. replication of practices which would not serve patients well) [13,27,28,37,50,57,63,64,70,71]. Concerns were also raised about the potential risk of disrupting good practice by imposing interventions based on assumptions rather than understanding of what had been occurring prior to the intervention [41].

**Table 5:** **Potential theories**

		**Implementation aspects**	**Intended outcomes**
**…models for learning**	Educational alliances [10]	•Need to trigger interpersonal connections between trainee and supervisor	•Educational alliance – defined as partnership producing just the right amount of responsibility – a balance between support and challenge with professional acting as safety net for patient and trainee
	Beacon practices [67]	•Need to trigger inter-practice links	•Collaborative and extended roles in primary care for professionals
		•Contextual infrastructure required	
	Communities of practice [26,40,48,74,76]	•Need to trigger genuine team-working between patients, trainees and professionals	•Harnessing of emergent learning from practice and experience
		•Trust required between all and relationship building a crucial mechanism for interventions to work	•Dynamic approach to care aligned to shared goals
		•Studies of actual working practices including during interventions needed	•Able to capture in-practice learning and innovation to further develop and improve outcomes (emergent learning)
		•Any intervention needs to focus not just on education or decision-support for individuals but also the dynamic system in which they are situated	•Reciprocal learning and sharing of best practice through system adjustments to support this
			•Development of communities of practice
	ExBL [11,77]	•Need to trigger ‘virtuous learning cycles’ – participation, balance of support and challenge, graded responsibilities	•Practical competence
			•State of mind conducive to practice (confidence, motivation, sense of professional identity)
	Breakdowns [78]	•When a breakdown (a situation where a person is not achieving expected effectiveness) occurs then interventions must trigger reflective learning and an effective response from others	•Constructive learning for future practice
		•Contextual factors: patient engagement, responsibility matched to authority, tools matched to task, information resources matched to need, values shared between co-participants, expectations matched to capacity	
	Developmental space [79]	•Creation of developmental space to permit learning and development of professional identity – space created through workplace context, personal and professional interactions and emotions such as feeling respected and confident	•Mindful learning and development
**…models for care delivery**	Guided Care [66]	•Increased staff resources for patient support	•Increased satisfaction with communication and increased knowledge of patient clinical characteristics
	Patient Centred Medical Home [48,61]	•Need to trigger social, psychological and physical assessment	•Holistic care developed through patient and professional collaboration
		•Need to trigger active patient and professional participation	
	CARE approach [57]	•Need to trigger connections between patients and professionals	•Holistic assessment, appropriate responses and patient empowerment
	Chronic Illness Care Plans [27]	•Need to trigger holistic assessment – requires professionals rethinking their roles	•Individualised care plans
	The Chronic Care Model [1,25,48,62]	•Need to trigger a patient centred approach including relational and management continuity	•Holistic care shared between patient and provider
		•Need to trigger reciprocal learning	•Sharing of best practice
		•Contextual factors are community resources and policies	
	Self-management support five A’s [65]	•Need to trigger assessment, appropriate advice, agreement of goals, assistance in behavioural change, and monitoring	•Personal action plans for patients and increased purposeful self-management
		•Context ‘self-management’ of some sort is inevitable as clinicians are only present for a fraction of a patient’s life	
	Shared decision-making [80,81]	•Need to trigger desire for patient involvement (varies according to reason for encounter)	•Appropriate shared decision making
		•Mechanism – education of health professionals about sharing decisions alongside patient mediated interventions	
**…models for both**	Transformative learning [44-46]	•Triggers are lived experiences combined with readiness for change leading to critical reflection, restructuring of meanings and development of new meanings	•New rules, ways or guidelines, new behaviours, feelings, beliefs, perspectives, identity
			•Learning about self and chronic illness in an iterative and continually changing manner
	Response shift [44]	•Triggers are lived experiences combined with readiness for change leading to critical reflection, restructuring of meanings and development of new meanings	•New rules, ways or guidelines, new behaviours, feelings, beliefs, perspectives, identity
	Education centred medical home [47]	•Need to trigger legitimate participation of trainees in continuity of patient care	•Increased patient support
			•Practice based learning experiences

**Figure 3:**
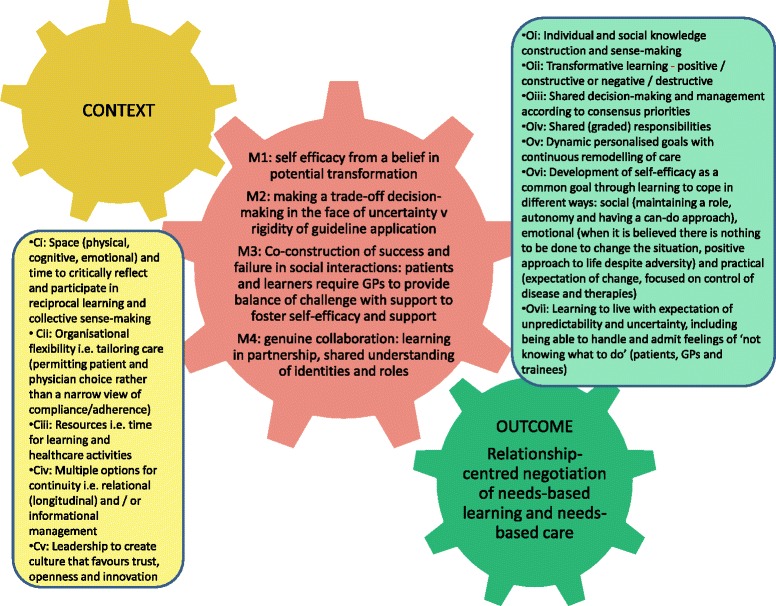
**Consider each tooth on each cog to represent a facet of context, mechanisms or outcomes.** With all elements in place the outcome cog will turn at maximum pace. With teeth missing on any of the cogs each will still turn and influence the next but less efficiently. Without any part of the context, the triggering of all the mechanisms is less likely and, therefore, interventions to improve education and healthcare in multimorbidity are at risk of failure due to lack of attention to social processes and education.

The synthesis identified important elements of the contexts in which patients, GPs and trainees interact for achieving relationship-centred negotiation of needs-based learning concurrent with needs-based care. These are space (physical, cognitive, emotional) and time to critically reflect and participate in reciprocal learning and collective sense-making, organisational flexibility with respect to tailoring of care (permitting patient and professional choice rather than a narrow view of compliance/adherence), resources including time for learning and healthcare activities and multiple options for continuity. Figure 3 summarises this and demonstrates how multiple, socially-constructed, contextual elements can cultivate social mechanisms to increase the likelihood of achieving relationship-centred negotiation of needs-based learning and need-based care through increasingly the probability of outcomes that together lead to this goal.

The limitations of currently available data prevent deeper exploration of the so-called realist ‘black box’ [5] beyond what is presented in this paper at present. It is likely that no one facet of context, nor one mechanism alone, will be sufficient to produce the desired outcome in full but further empirical research is required to establish this. We can describe the multifaceted details of our primary outcome but we cannot further breakdown exactly which contexts and mechanisms contribute to each facet. Given the presence of human agency in social interactions this may never be achievable. It should, however, be noted that this synthesis has considered a genre of programmes rather than a single specific intervention or series thereof. The idea that professionals can learn in workplaces is in essence a programme theory, but despite this, interventions to provide such learning opportunities are rarely as tightly designed or controlled (or controllable) as other interventions, for example, to improve public health. However, it might be possible empirically investigate how an intervention can produce a small part of the outcome described. Therefore, at present the best expression that can be achieved of a realist formula as a result of this synthesis is: (Ci + Cii + Ciii + Civ + Cv) → (M1xM2xM3xM4) → O, (where O is multifaceted as described by Oi – Ovii) [82] (see Figure 3 for more details).

Assuming the desired contextual elements are present and/or cultivated (e.g. through organisational culture, supporting structures and external influences such as healthcare and educational institutions) the results of this synthesis indicate that the key mechanisms for achieving relationship-centred negotiation of concurrent needs-based learning and needs-based care are:

- Self-efficacy from a belief in potential transformation
- Making a trade-off between decision-making in the face of uncertainty versus rigidity of guideline application
- Co-construction of success and failure in social interactions: patients and learners require GPs to provide balance of challenge with support to foster self-efficacy and support
- Genuine collaboration: paradigm of learning together in partnership, and team working with shared understanding of identities and roles.

These four mechanisms are, however, likely to also be made up of component parts. For example, individual and social knowledge construction and sense-making, transformative learning (can be positive/constructive or negative/destructive), shared decision-making and management according to consensus priorities, shared (graded) responsibilities, and personalised goals with continuous remodelling of care to meet these as they evolve are elements that could be identified within the synthesis data that could contribute to each of the four mechanisms identified. The current state of the literature is such that it was not possible to pursue further understanding of the implications of each mechanism potentially containing inter-related component parts further.

### A model of specific program elements to achieve desired outcomes

**Figure 4:**
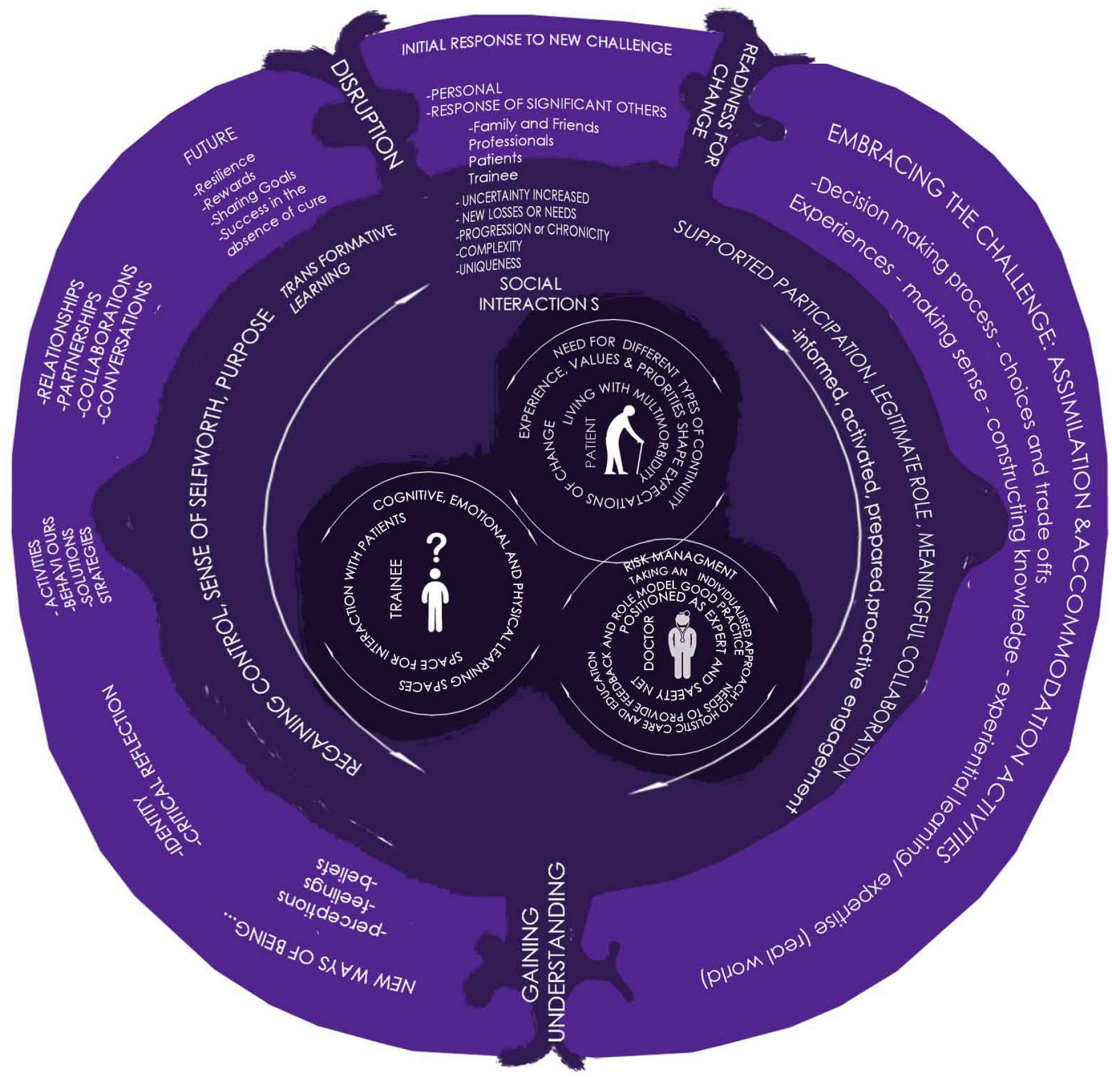
**This model represents the highest level of abstracted interpretation achieved during the synthesis.** There is no pre-defined starting point in this model as it is intended to represent complex, non-linear, fluid social interactions between patients, doctors (in this instance, GPs) and trainees. These three groups are interdependent in generating responses to the challenges of multimorbidity, that at the most constructive will produce a form of transformative learning for all three groups. At the centre of the model are represented each of the groups, with their most pertinent concerns, as identified in this synthesis. The inner loop surrounding this suggests potential mechanism for achieving optimal learning. The outer loop represents the potential for cycles of new understanding and new ways of being which are triggered by disruptions secondary to the ever changing impact of multimorbidity, combined with ‘readiness for change’ in the three groups. This model should be considered as representative of the level of theory development possible from current literature, as synthesised, and viewed in conjunction with Figure 3.

As a result of this synthesis, the emerging concepts arising from empirical findings and the identified socio-cultural, experiential learning and transformation theories have been mapped into a novel programme theory that outlines concurrency of clinical practice and learning in the context of multimorbidity in primary care (see Figure 4). The model outlines the contexts and mechanisms which optimise the chances of achieving the desired outcome of relationship-centred negotiation of needs-based learning and needs-based care. It encapsulates our new emergent theory of how social interactions can function as mechanisms to trigger constructive transformative learning for GPs, patients and trainees with respect to multimorbidity in primary care.

The ‘artistic’ representation is a deliberate representation of the non-linear processes which underlie the social interactions which act as ‘learning’ triggers and experience. These processes may recur and reconfigure as individuals/groups seek to make sense and understand the complexities of multimorbidity.

Experiential learning should provide a mechanism for trainees to put their knowledge into practice. From a patient’s perspective experiential learning related to multimorbidity is not a choice, but simply the means by which they come to understand their ever changing health and illnesses. Strategies such as self-directed care and self-management as well as shared-decision making draw on this experience, seeking to integrate it with professional knowledge and support. For both patient and trainee, therefore, experience is supposed to lead to greater knowledge and understanding about how best to manage multimorbidity.

Underlying these ideas are socio-cultural perspectives on experiential learning theories. Real life experience influences knowledge construction and sense-making through the impact of interpersonal interactions on cognition, emotions and social behaviours. Everyone learns (something) from experience due to intrinsic human desires to engage in sense-making. Whether the emergent learning is constructive or not is dependent on contextual influences as well as interpersonal interactions. This means that learning from experience is, by definition, a transformative process.

At the centre of our model, patients, GPs and trainees are represented in ‘free-floating’ circles around which the most pertinent concerns from the perspectives of each of these groups can be found. These revolving circles represent the multiple possible ways in which interactions and experience can shape sense-making, learning and healthcare delivery in the context of resources available and organisational support (or lack thereof). In the context of primary care, mechanisms interplay to produce experiential expertise, that is, personal and social knowledge construction and sense-making among patients, GPs and trainees. The outcomes consequential to these mechanisms are fluid, and subject to change as the mechanisms can operate both ‘positively’ and ‘negatively’. Important contextual elements for these interactions are the supportive resources available and organisational culture.

The outer loop of the model represents the process of transformative learning for all three groups which is likely to be cyclical. This outlines the proposed necessary steps by which relationship-centred negotiation of needs-based learning and need-based care are achieved. The inner loop suggests mechanisms which need to be triggered by the context and interactions of the groups in order to precipitate these steps. These mechanisms are not claimed to exclusively lead to the desired outcomes but, in recognition of semi-predictable patterns (demi-regularities) in human choices, may be necessary elements.

Learning, meaning and identity are again interlinked, as suggested by theories of transformation. To optimise learning, a trainee needs to be recognised as, and perceive to be, legitimately part of their practice and be supported to move from peripheral to central engagement in activities of the practice, learning along the way. The additional gains to be made through expert support can also be interpreted through theories such as Vygotsky’s zone of proximal development [33]– that is, learning is still seen as critically dependent on interaction with others who can extend the meaning-making and knowledge construction beyond that which one might reach independently. The model can, therefore be considered to have multiple layers of mechanisms: social interactions which are naturally occurring, elements of collective and collaborative approaches to the challenges of multimorbidity, and transformative learning. When these mechanisms are functioning constructively (positively) then it is possible for relationship-centred negotiation of needs-based learning and need-based healthcare to result. Variety in any of these elements can influence individual and collective learning arising from social interactions. It has been well established that context and the potential for genuine participation are particularly important for potentiating learning in workplace-based experiences [83]. Equally important is the need to distinguish intended learning in ideal circumstances from experience in practice when other factors (e.g. pressure of time or completing values and priorities) may lead to unpredictable consequences.

## Discussion

This realist synthesis sought to answer ‘What is known about how and why concurrent healthcare delivery and professional experiential learning interact to generate outcomes, valued by patients, GPs and trainees, for patients with multimorbidity in primary care?’ Based on these findings a refined programme theory for producing relationship-centred negotiation of needs-based learning and needs-based care proceeds as follows.

How patients, GPs and trainees construct their ideas about success and failure in multimorbidity matters because these concepts will influence their priorities, goals, and interactions. These, in turn, influence (i) care received including decision–making in the face of uncertainty, (ii) evolution of interactions between patients, GPs and trainees according to experientially-based knowledge construction and meaning-making about multimorbidity, and (iii) the replication of current clinical practices in the learning of future doctors [4,11,83]. It does not make sense to develop separate idealised models for delivery of care and education when both happen in the same place, at the same time, with the same people. Understanding real world practice is an important contextual element [83-85]; without this, any solution to develop a sustainable workforce to meet patients’ needs cannot adequately account for the influences of human interactions or specific problems in current healthcare delivery. The proposed model for designing future interventions is not intended to suggest that ‘transformation’ is an automatically utopian process, nor will it always produce constructive outcomes. Rather a fresh consideration of transformative learning, defined as accepting that life’s experiences act as stimulants to sense-making which will influence knowledge construction, assimilation (new beliefs, rather than simply tokenistic mastery to meet the demands of others) [54] and interpersonal interactions is important for understanding how social interactions shape the success of interventions in everyday practice. Professionals need to be trained to recognise and support the phases of transformation that individual patients are in and tailoring of management plans needs to take this non-linear learning into account.

### Strengths and limitations

While the size and complexity of this synthesis has been challenging, it was necessary to reflect and understand the complexities of interactions between patients, trainees and GPs.

This synthesis specifically considered interactions between three populations: patients with multimorbidity, doctors who, as fully qualified GPs, are responsible for both workplace supervision of trainees and the care of patients within primary care settings and trainees seeking to become doctors. We acknowledge that other people, (the significant others of patients and other professionals) and service constraints are part of the social world within which these interactions take place.

Literature syntheses are inherently dependent on primary studies and our review identifies gaps in the literature. In conducting this synthesis, review of potential evidence may not have been exhaustive. However, we believe that we have developed a suitably robust search strategy to identify the most relevant literature and this combined with the iterative approach described in the study protocol and methods (above) has allowed us to purposively select evidence to inform concurrency as well as concepts of success and failure in the absence of cure. This is important for the prioritisation of further research.

A realist synthesis seeks to analyse evidence in order to understand interactions between context, mechanisms and outcomes. Given the focus on reasoning, preferences, norms and collective beliefs (conceptualisations) in this synthesis it was logical to draw on literature in which the participants – patients, GPs and trainees outline their theories and angst about multimorbidity and how learning and care function in this context as well as more traditional sources of empirical and theoretical evidence. It can be seen from the descriptive summary of the types of evidence used in this synthesis that robust research evaluating the implementation of specific interventions to improve learning, and hence sustainable healthcare delivery in the context of rising multimorbidity is needed.

We also accept that the literature may, or may not reflect ‘real world practices’ of trainees, patients and GPs due to a paucity of studies using methods able to record and critically analyse concurrency in action.

Due to the inclusive definition of ‘intervention’ used in this study, and the relative absence of pre-designed interventions in the literature, much of the empirical evidence included in this review is derived from ‘natural experiments’. The choice to include such evidence is considered to be a strength of this work as it reflects the reality of the situation, in all its complexity. We considered this approach superior to solely focusing on individual purposively designed interventions implemented in controlled circumstances given our research question and objectives.

We recognise that our model, as a candidate programme theory, cannot be considered definitive until it is tested and refined in the ‘real world’. What we are proposing here is a model of elements for consideration by designers of, and participants in, workplace-based learning in the context of multimorbidity in primary care. Nonetheless, this model may also have wider applicability as a framework in for evaluating and/or redeveloping clinical workplaces where learning and care delivery occur simultaneously.

### Situating findings in existing research and theory

A recent Cochrane review found only eight studies of complex interventions focused on multimorbidity [9]. None of these considered learning or the creation of a sustainable medical workforce. Many of the concerns and failures identified in our synthesis resonate with the work of Sinnott *et al.* [86]. They found four areas of specific difficulty: disorganisation and fragmentation of healthcare; inadequacy of guidelines and evidence-bases; challenges in delivering patient-centred care and barriers to shared decision-making. Reaching shared understanding of goals is crucially important as it has been suggested that goal divergence between patients, caregivers and professionals tends to occur when patients are more medically unstable [87]. Following completion of our synthesis, Dugauy *et al.* [88] published an analysis of multimorbidity from patient perspectives which endorse our interpretations: the patients in their study describe cycles of crisis and accommodation during their experiences of living with multimorbidity with a need to take ownership of their health concerns while relying on professional interactions to assist with coping with uncertainty, complexity and simply loneliness arising from their circumstances.

Our work also resonates with education studies. Steven *et al.* [89] recently studied how undergraduate medical students learn from real patients in practice settings through analysis of audio-diaries. These demonstrated that participation in the practices of workplaces, including direct patient care, was essential for high yield learning but that education was not always coupled with patient care. When coupling did occur through expert supported dialogue, optimal results were produced. van der Zwet *et al.* [90] have reported on the importance of ‘developmental space’ for learning. This space describes a socio-cultural conceptualisation of the intertwining of workplace context, personal and professional interactions and individual affective states to produce ‘space’ for ‘development’. Werne *et al*’s [10] review of the literature on GPs as workplace supervisors in postgraduate clinical education found that a key skill of the supervisor was to intertwine clinical and educational activities. This combined with the formation of relationships with learners (educational alliances) and provision of appropriate challenge and support was necessary for learning and developing roles. More recently Ahern *et al.* [79] have reported on the potential for shared learning across multiple levels of learners as a means to address supervisory and access capacity concerns with increasing numbers of learners requiring experience in general practice if the needs of an aging population are to be met. Ahern *et al*’s findings again emphasised the need for GPs to foster collegiality among learners; these GPs need to be able to manage the social processes and dynamics between different trainees to achieve this.

### Implications for practice

For this synthesis multimorbidity was defined as the ‘co-existence of two or more conditions, where one is not necessarily more central’ [1] In practice, a definition of multimorbidity as ‘a set of unique constellations of problems, shifting priorities and multi-dimensional decision-making’ is more likely to resonate with patients, GPs and trainees [60]. Our synthesis suggests the following items are important for practice development until further research evidence is available:

1. Concurrency of education and care delivery must not be ignored.
2. GPs should be aware of the non-linear nature of transitions for patients and trainees occurring during social interactions and seek to support both in active engagement and meaningful roles by taking responsibility for legitimising participation and providing a safety net which balances challenge with appropriate support.
3. Primary care organisations should seek to create contexts in which patients, GPs and trainees can discuss challenges related to multimorbidity, concepts of success and failure and develop shared goals.
4. Recognition of different sorts of knowledge and practice (including experiential expertise) as valuable for development of new in-practice knowledge is important for patients as well as trainees and GPs.
5. Trusting relationships must be cultivated between patients, GPs and trainees.
6. Interventions should be designed to take account of the dynamic systems in which people work, accounting for breakdowns and work-arounds in interventions (and learning from these) as well as targeting education at individuals.
7. A reduced emphasis on index condition and diagnosis-cure models in long-term conditions is needed.

### Implications for research

Robust research evaluating the implementation of specific interventions to improve learning concurrent to healthcare delivery, and hence sustainable healthcare in the context of rising multimorbidity is needed. Further, work to identify meaningful markers of quality and success that match with patient and clinician priorities in multimorbidity is required.

The existing literature in this area suggests that work drawing on theories of transformation and socio-cultural processes should be pursued to understand and optimise outcomes for patients, GPs and trainees. The almost total absence of study designs to evaluate (i) experimental interventions for concurrent experiential learning or healthcare delivery in the context of multimorbidity or (ii) the impact of interventions on a naturally occurring state of concurrency between workplace-based experiential learning and healthcare delivery demonstrates these are also areas in which further research is needed.

## Conclusions

This study is novel in considering empirical evidence from patients, GPs *and* trainees engaged in concurrent learning and healthcare delivery. The findings should inform interventions to produce a workforce equipped to provide multimorbidity care. Omitting to account for concurrency of learning and healthcare delivery in interventions risks failure to implement, sustain or achieve potent outcomes even if the intervention had significant potential. Failure to recognise how patients, GPs and trainees conceptualise success and failure carries similar risks as people are most likely to be motivated by improvement innovations that resonate with their own personal motivations and goals.

## Endnotes

^a^Needs-based care is defined as the delivery of health and social care, whether this is in the form of treatments, service provision or other interventions according to patient need, rather than by diagnostic grouping. For example, patients with cancer and those with other potentially progressive diseases would not receive different levels of help and support due to differences in diagnosis but each patient would have their individual needs assessed before appropriate personalised care was offered.

^b^Needs-based learning describes targeting learning opportunities and experiences to the needs of individual learners, rather than taking a ‘one-size fits all’ approach or focusing on what is taught instead of what is learnt.

^c^A programme theory is a theory of how an intervention (or program) works.

^d^Programme elements are the parts of the programme or intervention including contextual elements and mechanisms including social interactions/designed pathways or plans to follow.

^e^Context describes the setting in which a particular outcome is being studied, in this case the context is both primary care and specifically multimorbidity [3].

^f^Mechanism is used in realism to describe the causes, processes or agents and structures within a social setting that lead an outcome to arise, specifically mechanisms describe the sequences of actions, events, interactions and subsequent events that lead to the generation of particular outcomes in particular contexts. It should be noted that in the realist context causes are not considered to be simple, linear or deterministic. People can choose to change their behaviour at any moment. Mechanisms are a product of this and the context in which people are situated. Within this synthesis we have used socio-cultural theories to guide our search to better understand how social interactions (mechanisms) lead to relationship-centred learning and needs-based care in the context of multimorbidity in primary care. In essence, as we identified a tentative theory of transformation during the synthesis, we have then sought to understand from the synthesis data ‘what is it about social interactions between GPs, patients and trainees that leads to positive transformative learning?’ [91].

^g^Outcome describes the desired product of an intervention or interaction that is designed to trigger it. A context – mechanism – outcme (CMO) configuration seeks to spell out the relationship between identified features of each. The model presented in this paper seeks to do this as it pictorially represents a possible mid-range theory of how social interactions generate useful learning/transformation (or not) [3].

## Additional files

Additional file 1:
**Full study report containing additional documentary support for the synthesis.**
Additional file 2:
**Search strategy.**
Additional file 3:
**Citation list.**
Additional file 4:
**Data extraction sheet v1.0.**
Additional file 5:
**Data extraction sheet v2.0.**

## Abbreviations

CMO: Context, mechanisms and outcomes
EI: Empirical interventional
ENI: Empirical non-interventional
ExBL: Experience based learning
GP: General practitioner
LR: Literature review
PROSPERO: International prospective register of systematic reviews
RAMESES: Realist And MEta-narrative Evidence Syntheses: Evolving Standards
TO: Theoretical/opinion
VICTORE: Volitions, implementation, contexts, time, outcomes, rivalry, emergence
