# Supplementary material for: Modelling successful primary care for multimorbidity: a realist synthesis of successes and failures in concurrent learning and healthcare delivery
Source: BMC Fam Pract. 2015 Feb 25;16:23. doi: 10.1186/s12875-015-0234-9 (PMC4343192; doi:10.1186/s12875-015-0234-9)
Supplement: Additional file 1: — Full study report containing additional documentary support for the synthesis. [file 12875_2015_234_MOESM1_ESM.pdf]

Concurrent learning and practice in primary care: using realist synthesis to model success, failure and social interactions in multimorbidity

Sarah Yardley, Elizabeth Cottrell, Jo Protheroe

With thanks to our collaborators Anne Warrell, Adele Higginbottom, Eliot Rees, & Harrison Carter

ABSTRACT

Background Human actions and interactions are at the heart of healthcare delivery and experiential learning. Interventions for either rely on adoption into everyday practices, with or without modification by the people involved. In clinical workplaces experiential learning occurs concurrently with healthcare provision. However, there has been little critique in the literature of how these two processes are negotiated simultaneously by patients, trainee doctors and general practitioners (GPs). The impact of multimorbidity on the educational needs of trainee doctors and general practitioners is little understood. Although the construct of doctor as healer, (with cure the ultimate marker of success), remains a strong narrative in society, in the context of rising morbidity from multiple incurable chronic illnesses this is problematic.

Primary care training sites must reconcile two different goals: (1) provision of appropriate individualised healthcare and (2) constructive workplace-based learning for trainees as future practitioners. This realist synthesis addressed the question: 'what is known about how and why concurrent healthcare delivery and professional experiential learning interact to generate outcomes, valued by patients, trainees and GPs, for patients with multimorbidity in primary care?' Prior to commencing the synthesis the research team theorised that identifying how success and failure were conceptualised (by patients, trainees and general practitioners) in the absence of cure would be essential to addressing this question.

Objectives

1. To produce a descriptive summary of what is known about relationship-centred negotiation of needs-based learning and needs-based care with a focus on models of patient care and/or workplace learning
2. To develop understanding of mechanisms at play when healthcare delivery and workplace-based education are occurring concurrently
3. A synthesis of conceptualisations of success and failure in the absence of cure, identifying how this affects learner and patient outcomes in the context of multimorbidity in primary care

Design Workplace-based experiential learning and healthcare delivery regarding multimorbidity in primary care are complex (and interacting) social interventions. We have used realist synthesis to systematically review healthcare, social science and educational literature for evidence. Realist synthesis starts with identification of potential outcomes, and aims to make sense of these by focussing on interaction between mechanisms and contexts. For this review, relationship-centred negotiation of needs-based learning together with needs-based care was the primary outcome of interest.

Published standards informed the study design[1] (PROSPERO registration: CRD42013003862).[2] A literature search, using terms pertaining to (1) multimorbidity, (2) primary care, (3) education and (4) workplace experiences was undertaken in 20 databases followed by further iterative searching. Articles covering at least one item of healthcare delivery, education or social processes were accessed for quality screening and data extraction. Data extraction focused on 'CMO' (context, mechanisms and outcomes) configurations within studies and on data which might assist understanding and explain; i) the CMO configurations themselves; ii) the relationships between them and; iii) their role and place in evolving programme theories arising from the data synthesis. Each article was reviewed by SY in addition to at least one other team member who subsequently discussed the content to reach consensus on data extractions. Mind-mapping software was used to synthesise data into an interpretative analysis. Team meetings hosted discussions of emerging findings.

Results The final synthesis included: five policy initiatives (PI), eight empirical training interventions (ETI), 12 literature reviews (LR) (one published twice, total = 13 papers), 48 theoretical/opinion papers (TO) and 67 empirical non-interventional studies (ENI), totalling 141 papers of which 34 papers contained models (2ETI, 13ENI, 3PI, 4LR and 13TO) for workplace-based experiential learning and/or patient care. Models of experiential learning for practitioners and for patient engagement were congruent. Both frequently included reference to theories of transformation and socio-cultural processes as mechanisms for improving clinical care.

---

[1] Wong G: RAMESES publication standards: realist synthesis. *BMC Medicine* 2013, 11:21 doi:10.1186/1741-7015-11-21

[2] Yardley, S., Cottrell, C., Protheroe, J. Understanding success and failure in multimorbidity: protocol for using realist synthesis to identify how social learning and workplace practices can be optimised. *Systematic Reviews* 2013, 2:87 doi:10.1186/2046-4053-2-87

Key issues included the incommensurable nature of personalised concepts of success with measurability of clinical markers or adherence to guidelines, and the need for greater recognition and evaluation of experiential learning and social dynamics between patients, trainees and GPs including complexities of shared responsibilities.
Conclusions In the context of multimorbidity in primary care we propose a model which considers the educational and healthcare delivery implications of concurrent learning and healthcare delivery. Our model takes account of potential patient, trainee and general practitioner conceptualisations of success and failure in the absence of cure and how these may impact on their social interactions with each other. In this report we present the following evidence in support of the model: (1) context-focused refinements to the original theory that socio-cultural mechanisms are important to explain learning and reconciliation of goals in multimorbidity, and; (2) products of the synthesis including (a) a summary of empirical and model-based studies specific to multimorbidity in primary care, (b) theories of relevance identified in the literature linked to implementation actions and intended outcomes, (c) essential elements for productive concurrency of workplace-based learning and sustainable patient care as mediated through social mechanisms. The model brings together these elements in a blue print for the design and testing of new initiatives to address the integration of learning and practice. This synthesis is important as within the previously existing literature there was a significant gap due to a lack of work drawing on empirical evidence from patients, trainees *and* GPs engaged in concurrent learning and healthcare delivery. However, to bring together these perspectives, and seek to understand the complex 'real life' interactions between patients, trainees and GPs the synthesis was necessarily large and complicated. By informing the direction and design of research-based interventions this work can aid efforts to produce a sustainable medical workforce equipped to provide multimorbidity care and ensure continuity of professional and patient education about the same. *REGISTRY AND NUMBER: PROSPERO, CRD42013003862*

CONTENTS

1. Introduction P5
PROVIDING MULTIMORBIDITY CARE
EDUCATION ABOUT MULTIMORBIDITY IN PRIMARY CARE
COMBINING PATIENT CARE AND EDUCATION FOR TRAINEES
SOCIO-CULTURAL MECHANISMS TO EXPLAIN THE PROCESS UNDERLYING RECONCILIATION OF GOALS
CONCURRENCY
REALIST SYNTHESIS

2. Aims and objectives P8

3. Methods P9
ENGAGEMENT WITH FUTURE PRACTITIONERS: TRAINEES
EXPLANATORY SCOPING TO FINAL SEARCH STRATEGY
CHANGES IN THE REVIEW PROCESS
JUDGEMENTS ABOUT DATA EXTRACTION
DATA SYNTHESIS

4. Results P12
DESCRIPTIVE SUMMARY OF THE SYNTHESIS
CONCURRENCY OF HEALTH SERVICE DELIVERY AND PROFESSIONAL EXPERIENTIAL LEARNING IN THE CONTEXT OF MULTIMORBIDITY IN PRIMARY CARE
PATIENTS', TRAINEES' AND GPS' EXPERIENCES AND CONCEPTUALISATIONS OF MULTIMORBIDITY IN PRIMARY CARE
REFINEMENTS TO ORIGINAL PROGRAMME THEORIES
A MODEL OF SPECIFIC PROGRAM ELEMENTS TO ACHIEVE DESIRED OUTCOMES

6. Discussion P30
IMPLICATIONS FOR PRACTICE

Acknowledgements **P36**
Citation list of articles included in synthesis **P36**
Appendices **P45**
1. ADDITIONAL SENSITIVITY SEARCH IN MEDLINE DATABASE
2. SUMMARY OF STUDIES CONTAINING MODELS / SUMMARY OF OTHER EMPIRICAL STUDIES
3. MARKERS OF SUCCESS AND FAILURE INDENTIFIED IN THE SYNTHESIS

Endnotes **P72**

INTRODUCTION

PROVIDING MULTIMORBIDITY CARE

In the context of an aging population the experience of living with multiple chronic diseases is increasingly common. Aside from demographic changes, advances in medical science have led to a blurring of boundaries between long-term illness, life-limiting illness and end-of-life care all of which exist alongside the concept of survivorship. Multimorbidity has been defined as the 'co-existence of two or more conditions, where one is not necessarily more central' [20]. Despite multimorbidity being a significant and increasingly prevalent problem among the primary care population, aside from epidemiological work, the impact on provision of healthcare and/or workplace-based experiential learning is little understood. There is a tendency for 'patient-centred care' and/or 'integrated care' to be proposed as solutions to this in contemporary policy-making, but little is known about realities of negotiation of care in practice or how issues in multimorbidity which supersede pathways for specific diseases are handled.

Although the construct of doctors as healers, with cure the ultimate marker of success, remains a strong societal narrative, it is problematic in the context of multimorbidity. Identifying more appropriate definitions of success and failure in the absence of cure was seen as key to developing an understanding about how and why social interactions lead to different learning and care delivery outcomes in this context and underpinned this work.

EDUCATION ABOUT MULTIMORBIDITY IN PRIMARY CARE

General Practitioners (GPs) working in primary care training sites have the additional challenges presented by the presence of trainees and therefore they must reconcile two different goals: (1) provision of appropriate individualised healthcare and (2) constructive workplace-based learning for trainee doctors. In this work the term 'experiential learning' is used to describe any learning, undertaken by anyone that arises from workplace-based interactions, that is, the creation of meaning or construction of knowledge from 'real life' experience. The term 'trainees' (abbreviated from trainee doctors) will be used to refer to both undergraduate students and postgraduate trainees. This permits the term 'learning' to be used as needed without automatically signifying a particular group.

COMBINING PATIENT CARE AND EDUCATION FOR TRAINEES

Disease-specific care provision and education carries significant risks of fragmentation of care and creating or perpetuating uncertainties about managing one condition in the context of another. Interventions are needed that will produce a sustainable medical workforce equipped to provide healthcare, when cure is not possible. Development of experience and expertise to achieve this is dependent, in part, on the recognition and acceptance of trainees as legitimate participants in healthcare activities, including holding appropriate responsibilities for patient care.

SOCIO-CULTURAL MECHANISMS TO EXPLAIN THE PROCESS UNDERLYING RECONCILIATION OF GOALS

A socio-cultural perspective on experiential learning recognises that people continuously rearrange their thinking in the light of new experiences and consequences arising from social interactions. Ever since Dewey it has been argued that personal experience alters the quality of understanding.[i] Equally pre-existing knowledge influences future learning.[ii] Mezirow describes transformative learning as a

process which arises out of the intrinsic human need to make meaning out of experiences and to integrate experiential understanding with existing knowledge and beliefs. He focused his writing specifically on the transformative aspects of learning, defining transformation as 'the process by which we transform our taken for granted frames of reference (meaning perspectives, habits of mind, mindsets) to make them more inclusive, discriminating, open, emotionally capable of change and reflective so that they may generate beliefs and opinions that will prove more true or justified to guide action'.[iii] Active dialogue can enhance this process. This definition recognises the significance of pre-existing ideas on the potential for future learning as well as the need for constructive social discourse if experiences are to be maximised as learning opportunities. Once it is understood that for learning to occur, humans intrinsically make sense of life experiences and integrate new meanings with prior understanding, it becomes clear that learning a particular role can be intertwined with learning academic knowledge. When faced with challenges, the support of an experienced guide (e.g. a GP) can reduce improvisation when seeking solutions with its associated risks of unpredictable transformation.

Socio-cultural theory is not synonymous with experiential learning but provides a complementary perspective on how people can learn from experience. Context and process of learning are seen as indivisible influences on outcomes. Learning from experience is 'situated'[iv] – it happens in a particular place, at a particular time, with particular people in particular contexts. Interactions with others shape learning processes. Learning is seen as a collective activity[v] in which people may have common or conflicting goals. The context of their activities to achieve goals may or may not be facilitative. Workplaces exhibiting constructive, facilitative achievement of goals are often described as communities of practice[iv] while those without these features are much less acknowledged but do exist as dysfunctional communities of practice. It is important to realist that learning opportunities do not necessarily result in predictable and repeatable outcomes as most communities within workplaces will fluctuate in levels of functionality. This is highlighted by Eraut[vi] who suggests three questions which should be sought with respect to workplace based learning: what is being learned? How? and What is influencing the learning effort?

Experiential learning workplaces must produce doctors who can function to provide for the populations needs now and in the future. Dornan *et al* [40] have produced a generic model (ExBL) which illustrates the essential elements of experience-based learning. Their model represents one of the few pieces of work in this area specific to medicine, albeit focused on the early years of training. Key elements include participation with graded responsibilities as a core process in experiential learning, support for appropriate challenges and the importance of clarity regarding expectations. This includes being able to participate in shared decision-making and responsibilities. Experiential learning and the delivery of primary care to people with multimorbidity happen in the same places, at the same times and with the same people. Both patients and practitioners make meaning and construct knowledge during the trajectories of patients' lived experiences of multimorbidity. The learning processes occurring for patients with respect to informal and formal management of multimorbidity and impact are not dissimilar to the learning processes of trainees, or even of GPs. Fundamentally all learning is shaped by experience. How people reflect on, and make sense of experiences depends, in part on their interactions with other people during those experiences. However, patients and GPs may have differing priorities and conceptualisations of risk-benefit balance. Given that patients, trainees and GPs may have differing priorities and conceptualisations of success and failure in the absence of cure, achievement of both goals depends on choices which

influence social and cultural mechanisms for interacting with others. The choices people make (what they think, say or do, and other expressions of agency such as what they do not say or do), whether individually or collectively are important mechanisms for the generation of social outcomes.

CONCURRENCY

Workplace-based experiential learning and healthcare delivery may both be viewed as complex interventions which take place in the social world of patients, trainees and GPs, and so are shaped by their interactions. In turn, social interactions are influenced by how people think about challenges in daily life. In the context of multimorbidity in primary care we feel it is important to understand how people conceptualised success and failure in the absence of cure as this is likely to influence both learning and care delivery.

REALIST SYNTHESIS

This work commenced from the premise that socio-cultural theories could be used to identify and understand mechanisms at play when healthcare delivery is concurrent with workplace-based and experiential learning. Given that all healthcare is inherently dependent on human action, it is surprising how little explicit attention has been given to understanding how and why human action may strengthen or impede mechanisms for needs-based healthcare delivery and experiential learning to make this sustainable.

This realist literature synthesis addressed the question: What is known about how and why concurrent healthcare delivery and experiential learning interact to generate outcomes, valued by patients, trainees and GPs, for patients with multimorbidity in primary care?

This work treats both healthcare delivery and experiential learning in clinical workplaces as complex social interventions. Therefore realist synthesis was selected as the review methodology because it provides a rationale and tools for synthesising varied evidence about complex social interventions which involve human choices and interactions. It starts with identification of potential outcomes and aims to make sense of these by focussing on the associated interaction between mechanisms (or processes) and 'real life' contexts while seeking to explore 'what works, for whom, in what circumstances and why?'

Any interventions for either workplace-based learning or individualised care provision for people with multimorbidity found in the literature were unlikely to be simple. Traditionally 'an intervention' may be thought of as a 'program' that can be introduced to seek a particular outcome. In the context of this work the definition of an intervention is necessarily looser. For example, we have classified sets of human actions / interactions, undertaken together in the common belief that they will lead to particular outcomes as social interventions. However these actions/interactions may not be entirely specified and/or co-ordinated in any given context. Instead such social interventions draw on shared theories / principles. Such theories/principles might be collectivised into a 'mid-range theory'. For example, this applies to situations in which people are required to interact with respect to common goals despite the absence of an organised intervention. In general practice, for example, it is in the interests of patients, trainees and GPs to work to a common goal of safe practice through good communication, but this may be done without any specific and explicit intervention to achieve this goal. Given the lesser structure of such interventions, the effects of generating change in this way are often complex, complicated and not entirely predictable. This is because many processes (mechanisms in realist terms) occur, and formal or informal modifications are frequent, as

‘...social programs or interventions work by changing the decision-making of subjects’.[vii] These processes can easily remain invisible unless explicit attention is paid to how and why change does or does not occur, including the underlying theories.

The scope of this synthesis was limited to primary care for both contextual (most people with multimorbidity spend most of their time in the community) and pragmatic (a manageable study) reasons. The synthesis was also limited to trainees seeking to become doctors so that this professional trajectory from trainee to practitioner could be considered alongside triadic interactions between patients, trainees and GPs. We acknowledge that other people, both the significant others of patients and other professionals are part of the wider social world within which these interactions take place. Literature has been included as synthesis data if relevant to multimorbidity in primary care (as defined in the UK or the nearest international equivalent).

AIMS AND OBJECTIVES

This work seeks to identify what is known about how and why concurrent healthcare delivery and professional experiential learning interact together to generate outcomes valued by GPs, trainees and patients for patients with multimorbidity in primary care through:

1. Describing what is known about relationship-centred negotiation of needs-based learning and needs-based care with a focus on models of patient care and/or workplace learning
2. Understanding the mechanisms at play when healthcare delivery and workplace-based education concurrently occur
3. Synthesising conceptualisations of success and failure in the absence of cure, identifying how this affects learner and patient outcomes in the context of multimorbidity in primary care

Initial findings from the review identified a paucity of work addressing the interactivity between models of workplace-based experiential learning and models of patient care, despite the similarities and comparisons regarding socio-cultural processes. This observation combined with the identification of evidence which could be used to build understanding of how people define success and failure in multimorbidity when there is an absence of cure led us to focus our review on non-linear transitions that are mediated through social interactions. How these transitions occur will be influenced by levels of belief in the potential for (positive or negative) change.

This report considers the two main findings arising from the realist synthesis: (1) issues of concurrency and (2) conceptualisations of success (and failure) in the absence of cure. With this focus in this report we present:

1. A descriptive summary of the synthesis data with a focus on models of patient care and/or workplace-based experiential learning in the form of a conceptual map of existing evidence and theory refinements
2. Refinements to our original proposal that socio-cultural theories can be used to unmask and identify mechanisms at play when healthcare delivery and workplace-based experiential learning are occurring concurrently by the same people in the same place at the same time
3. Novel findings and new theories for testing arising from our synthesis of understanding about conceptualisations of success and failure in the absence of cure

A second report is in preparation in which we provide an interpretative synthesis of patients', trainees' and GPs' lived experiences of multimorbidity following a secondary analysis of qualitative data identified in the course of this review.

METHODS

As the protocol for this review has already been published[viii], this section provides a brief overview of the methods contained in that protocol with additional details where iterative developments took place during the review process.

The review team consisted of (1) clinical academic researchers with experience in systematic and narrative reviews, qualitative and quantitative research, medical education, social sciences and general practice; (2) patient and public involvement group members with experience of co-/multimorbidity from chronic conditions, and; (3) undergraduate and postgraduate medical trainees all of whom took active roles in data extraction and synthesis. In addition to this core review team a number of general practitioners with experience of educational supervision (and in some cases education research) at both undergraduate and trainee level provided expert advice during the synthesis process.[viii] Engagement with these 'experts' alongside engagement with future practitioners ensured that iterative developments in the course of the synthesis were focused on issues of relevance for educational and healthcare practices within primary care. Further details of engagement activity undertaken with trainees are given below before discussing the formal synthesis methods from exploratory scoping of the literature to completion of the synthesis.

ENGAGEMENT WITH FUTURE PRACTITIONERS: TRAINEES

During the initial stages of the review a series of informal meetings took place with GPs holding responsibility for training and medical education, academics, undergraduate and postgraduate trainees. These meetings highlighted the challenges of learning to care for patients with multimorbidity. Information gleaned in these meetings was used to direct and focus the aims of this synthesis, thereby improving relevance to practice.

Undergraduates reported a preference for learning about the complexities of multimorbidity during workplace-based experience but identified several challenges in doing so. The students' self-reported exposure to multimorbidity in primary care was not equivalent to the natural prevalence, possibly due to single-disease focussed curriculum learning outcomes and/or protection of students from complexity by GP supervisors. A lack of explicit integration between learning about medical management and learning about social impact of disease was noted. Students also suggested that a lack immediate relevance may have reduced recognition of the value of learning to deal with complexity when seeing patients during experiential learning sessions, particularly in the early years. This may have resulted in missed opportunities to learn about multimorbidity.

Postgraduate trainees agreed that multimorbidity was important and in-practice experience was an effective learning strategy, particularly when providing primary care in residential homes and when working with experienced nurses. Challenges identified by this group were: (1) feeling insufficiently equipped to decide if, when and how to deviate from single condition guidelines if, for example, there was a risk that the treatment of one condition could worsen another, (2) much higher exposure to multimorbidity in secondary care than in primary care which meant most of their experience related to crisis management rather than chronic care, (3) concerns about a tension

between most appropriate care (which they defined according to patient priorities) and meeting clinical targets (which they described as a professional pressure). These trainees described global uncertainty about their learning and ability to deliver care in the context of multimorbidity. Independent practice was recognised as risking missed opportunities for learning in primary care especially as many had not previously considered discussing multimorbidity-related learning needs with their training supervisors.

EXPLORATORY SCOPING TO FINAL SEARCH STRATEGY

The review sought to identify studies of any intervention purporting to provide workplace-based experience in managing the complexities of multimorbidity or lead to relationship centred negotiation of needs-based healthcare and experiential learning.

Preliminary searches of the literature were undertaken to develop search strategies (modified for individual databases) covering (1) multimorbidity, (2) primary care, (3) education, and (4) workplace experiences. Multimorbidity is not currently a MeSH term. Therefore, in addition to searching for this as a keyword (spelt as one or two words) MeSH terms for chronic disease and comorbidity were used as proxies.

In the final strategy these four threads were combined as (1) AND (2) AND ((3) OR (4)). No date, language or other limitations were used. In line with realist methodology searches were not limited by study design. Following comprehensive electronic searching in 20 databases (see Box 1) covering healthcare, education and social sciences literature, citation searches were conducted alongside review of relevant policies and reports in the grey literature. Further searches were conducted in Google Scholar and Web of Science as key author searches and identified experts in the field emailed for further suggestions. References of all included articles were searched for other relevant citations. The complete strategy is available in the protocol.[viii]

Box 1: Databases included in searches
(initially conducted on 1st Aug 2012, and updated via alert systems until 1st Aug 2013)

- Academic Search Complete
- Applied Social Sciences Index and Abstracts
- Australian Education Index
- British Education Index
- Best Evidence Medical Education
- British Nursing Index
- CINAHL
- Cochrane
- Embase
- Education Resources Information Center
- Health Management Information Consortium
- Joanna Brigg Institute
- Kellogg Foundation
- Medline
- Opengrey
- PsychInfo
- Science Direct
- Social Services Abstracts
- Sociological abstracts
- Web of Science

Articles were retained for review if they were about chronic disease, for example, chronic disease models, as likely to include multimorbidity, if about primary care and other similar settings (i.e. those in which the same mechanisms may be in operation). Articles were excluded through

consensus among the research team if they did not pertaining to healthcare delivery, medical education or social processes or if they were specifically about single diseases.

CHANGES IN THE REVIEW PROCESS

In the original protocol two rounds of searches are described. However, following completion of the protocol a specific gap in the synthesis data regarding models of best practice for experiential learning was identified. Therefore a third round of searching involving two iterative search strategies were implemented: (1) a sensitivity analysis of educational search strategy for purposive selection of citations on generic experiential learning and (2) re-screening of citations initially excluded as not relevant to primary care to identify if these contained data relating to concurrency of education and healthcare delivery which might be transferable to multimorbidity in primary care, or any intervention/model of potential use. For the sensitivity analysis we first cross-checked for any additional original articles identified in previous review work [40, 124, 138] relevant to our research questions to screen alongside other articles serendipitously identified by members of the research team. Second, we purposively selected from the literature on experiential learning with reference to two bodies of work among 1) undergraduates and 2) postgraduates. The first was accessed through searching for the 'experience based learning' (ExBL) model developed by Dornan *et al* over a number of years following an extensive body of empirical, review and theoretical work regarding experiential learning [40], particularly among undergraduates. The second of these was informed by a publication [138] in which the authors conducted an integrative review of GPs as supervisors in postgraduate clinical education. The search terms which they included, not used in our previous searches but with potential relevance to our objectives were: ambulatory care, role model, supervisor and supervisee. A renewed search was performed in Medline (see Appendix 1) and incorporated these terms into the original search to detect further work that may be relevant to postgraduate education. No other databases were used as from the previous search rounds it was apparent that use of Medline alone was sufficient to address the research objectives.

Following the first round of data selection (Diagram 1), 7294 citations were identified from 20 databases covering healthcare, education and social sciences. Articles were tagged for relevance to one or more of three areas of interest (health services (HS), education (Ed), social processes (SP)). Those with at least two tags were purposively selected for the initial synthesis. Round 2 included all other articles with at least one tag, plus citations identified through hand-searching the references of included papers / original papers identified through secondary sources and new publications identified on journal alerts set up in the chosen databases. Round three included citations identified through the additional searches described above plus further citation searching and new publications identified by journal alerts.

JUDGEMENTS ABOUT DATA EXTRACTION

In each round titles, then abstracts, then full text citations were screened. Exclusions at the title screening stage were made if the article was not relevant to primary care or symptomatic multimorbidity. At the abstract and full text screening stages, citations that were not relevant to healthcare delivery, nor education, nor social processes and/or interactions were excluded. Given the nature of this work an over-inclusion approach was taken such that citations were reviewed in the next stage if there was any uncertainty about relevance.

A data extraction sheet was designed to focus data extraction on 'CMO' (context, mechanisms and outcomes) configurations within studies and on data which might assist understanding and explain; i) the CMO configurations themselves; ii) the relationships between them and; iii) their role and place in evolving programme theories. In addition to CMO configurations, all citations were reviewed for elements relevant to the review question and robustness of the work, that is, was the method used to generate data rigorous, credible and trustworthy? . Each article was independently reviewed by at least two team members who subsequently discussed the content to reach a consensus on data extractions. With each round of data extraction, iterative modifications were made to the data extraction sheet to focus on elements most useful and still needed to best develop the review.

DATA SYNTHESIS

Data synthesis was led by SY although all members of the review team actively participated. During the synthesis the team engaged in iterative selection and testing of candidate theories and actively sought data to refute or refine these. This process led to the selection of transformative learning as a theory to pursue, when it emerged that this held commonality across patient and trainee trajectories.

As the synthesis progressed the initial focus (models of patient care and/or workplace learning) within our primary outcome of interest (relationship-based negotiation of needs-based learning and needs-based care) was refined to consider more specifically non-linear transitions in multimorbidity for patients, trainees and GPs. These transitions are mediated through social interactions and are influenced by levels of belief or non-belief in the potential for change or transformation. Re-focusing was necessary as it emerged that transitions were an important process for all three groups, with implications common to their interactions.

Mind-mapping software was used to develop an interpretative analysis by progressive assimilation and adaption of data extracted and creation of tentative links between different data sources. The seven key elements of any complex programme (as described by Pawson's VICTORE mnemonic[ix]) were used as a tool to guide our interpretations of interacting social processes. Team meetings hosted discussion of emerging findings.

Following this process we further discussed citations in which either a theoretical or empirical model relevant to our review purpose was presented. These papers are summarised in Appendix 2. Common ideas and theories within these citations were used to develop an integrated interpretation of a 'super-model' to describe current data on how and why conceptualisations of success and failure impact on non-linear transitions in multimorbidity for patients, trainees and GPs that are mediated through social interactions and are influenced by levels of belief or non-belief in the potential for change or transformation.

RESULTS

DESCRIPTIVE SUMMARY OF THE SYNTHESIS

Database searches identified 7294 unique citations of which 6233 were excluded through title screening. A further 888 citations were excluded through abstract (n=882) and full text screening (n=6) as these were not relevant to health services, education or social processes/interactions, or were judged to be of poor quality on the basis that the research did not support the conclusions

made. Of the remaining 173 full text articles reviewed, 43 were purposively selected for round one. In round two, 130 remaining articles, plus 124 newly identified articles were accessed from which 69 were included and 185 were excluded (as not covering health services, education or social processes/interactions, or poor quality). Round three produced an additional 29 articles. The final synthesis included 141 papers: 5 policy initiatives (PI), eight empirical training interventions (ETI), 13 papers describing 12 literature reviews (LR), 48 theoretical/opinion papers (TO) and 67 empirical non-interventional studies (ENI). Thirty-four papers contained models (2ETI, 3PI, 4LR, 13ENI and 13TO) for workplace-based education and/or patient care relevant to multimorbidity in primary care.

No papers identified contained empirical evidence from patients, trainees *and* GPs engaged in concurrent learning and healthcare delivery in a single model. Further, only two papers [24, 112] considered healthcare delivery, professional education and social processes together. The vast majority of papers describing experiential learning did not specifically relate to multimorbidity, although chronic illness care was included within the spectrum of learning discussed. Due to these limitations in the literature, transferability of models and theories required theoretically guided interpretation. Diagram 2 provides a conceptual map of the essential elements involved in the process of evidence synthesis used to refine our initial programme theories. On the left hand side in the blue box is an outline of the initial rough theories with which the literature was approached. In the centre (pink box) is a summary of the evidence reviewed, illustrating the types of evidence sought and the elements within that evidence that we sought to populate our CMO framework with. On the right hand side (purple box) are the emergent mechanisms which were identified as being important to reach our primary outcome of interest. As is indicated in the purple box this links to Table 5 which provides further details of the full CMO configurations which form our refined programme theories. In reality the processes of evidence synthesis were iterative, with movement back and forth between data (evidence from the literature) and theoretical refinement and evolution.

There appeared to be general agreement that patients with multimorbidity should receive personalised care which is tailored to their individual needs (see tables). Continuity, in its various forms, also emerged as important. Given that most affected patients primarily reside in community settings, focussing the work on primary care appeared to be appropriate as this is the setting within the healthcare system in which these goals could be achieved.

The synthesis highlighted a strong recognition of the complex realities of practice. In addition, two emergent themes were shared between patient experience and trainee experience. These themes were: (a) experiential and transformative learning, and (b) socio-cultural elements of success. These themes are discussed further in the results below.

Diagram 1: DOCUMENT FLOW

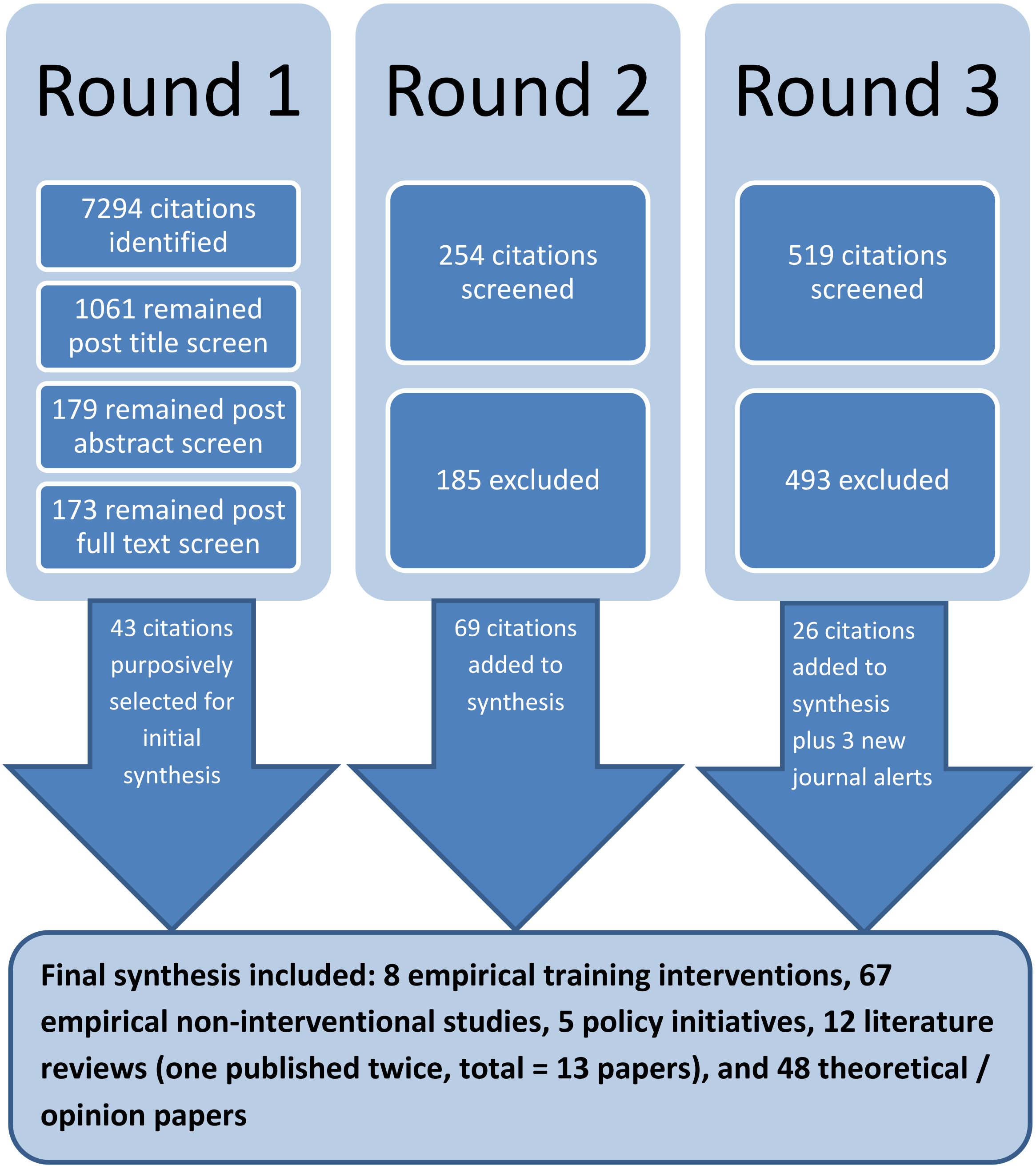

Diagram 2: CONCEPTUAL MAP

PROCESSES OF EVIDENCE SYTHESIS USED TO REFINE THEORY

INITIAL ROUGH THEORY

'Sociocultural theories can be used to identify mechanisms at play when health service delivery is concurrent with workplace-based and experiential learning'

THEORY OF ACTION

- Workplace-based learning and health service delivery occur concurrently with the same people, in the same places at the same time
- Experiential learning produces doctors able to function now and in the future to provide for the populations needs
- Shared decision-making and responsibilities

THEORY OF CHANGE

- Emergence of high quality practice (applied knowledge, skills, attitudes and behaviours) comes from workplace-based learning opportunities
- Improved learning opportunities and clinical outcomes from self-directed learning and self-management
- How people think about success and failure in the absence of cure will affect their interactions

REVIEW OF THE EVIDENCE

EVIDENCE ABOUT (1) concurrency of health service delivery and professional medical education and (2) conceptualisations of patients, doctors and learners (students and trainees) regarding success and failure in the absence of cure …

….IN THE CONTEXT OF multimorbidity in primary care

SEARCH TERMS (i.e. program elements)
(1) multimorbidity (2) primary care (3) education (4) workplace experiences

IMPLEMENTATION:
Is interactivity is important: (1 between people? (2) between learning and practice? And why?
Tools available to assess quality of care and learning, models for delivering care and learning, evidence of change in practice, understanding of risk – benefit balance in interactions and the influence of (unscripted) conversations in shaping learning and care
Chronic care models and educational alliances which permit direct learner-patient interactions

OUTCOMES:

- Primary outcome of interest: relationship-centred negotiation of needs based learning and needs based care: (e.g. stabilising the condition to get patients back to living their lives, support through monitoring and review, timely intervention by appropriate service when things go wrong, providing choice and support towards the end of life (Ref NHS long-term conditions)
- Alternative outcomes
- Outcomes in 'natural course of interactions between people' – evolution and replication of naturally occurring practices

HOW MECHANISMS GENERATE OUTCOMES: Social interactions and personal and social experiences leading to 'experiential knowledge and expertise': perceptions of success and failure shaping priorities and goals, understanding of roles and responsibilities, role modelling, handling of breakdowns, scaffolding and conferring legitimacy, trust, active roles, reciprocal learning, and interpersonal dynamics

HOW CONTEXT AFFECTS MECHANISMS AND OUTCOMES: 'Real world practice', 'Lived experiences', 'Pragmatism in expectations', 'Readiness for change', historical / policy focus on biomedical markers and clinical service outcomes, poor guideline resonance with individual patient circumstances, no one size fits all – complicated / complex, clinical microsystems which are complex and adaptive, time and other resources

REFINED THEORY

Self-efficacy from a belief in potential transformation*

Trade-off decision-making in the face of uncertainty v rigidity of guideline application*

Co-construction of success and failure: balance of challenge and support*

Genuine collaboration: paradigm of learning together in partnership, and team working with shared understanding of identities and roles*

Non-linear transitions for patients and learners: All participants 'buy-in', Individual and social knowledge construction and sense-making, transformative learning

* Mechanisms only represented here. See Table 5 PRODUCTIVE CONCURRENCY: WORKPLACE-BASED LEARNING AND SUSTAINABLE PATIENT CARE for full CMO configurations

CONCURRENCY OF HEALTHCARE DELIVERY AND PROFESSIONAL EXPERIENTIAL LEARNING IN THE CONTEXT OF MULTIMORBIDITY IN PRIMARY CARE

Concurrency and interactions have been inadequately studied to date. However, evidence suggests that students learn even when their teachers' minds are wholly focused on patient care [39]. Attention should be paid to learning situated within patient interactions, with GPs providing both challenge and support.

The only paper to consider the complexity between educational and clinical roles for GPs supervising training [138] presents a review of the relevant published literature and found that recommendations and descriptions outweighed empirical evidence. In practice, clinical and educational activities undertaken by supervisors are intertwined. Although supervisors formed educational alliances with trainees, a co-existent tension was ensuring that patient care was not jeopardised by learning. Supervisors reconciled this by seeking to provide both appropriate challenge and support to trainees.

Regarding concurrency, there appeared to be two key issues: 1) the incommensurable nature of personalised concepts of success with measurability of clinical markers or adherence to guidelines, and 2) the need for greater recognition and evaluation of how role modelling and interpersonal dynamics in social interactions impact on and influence experiential learning. With respect to the latter issue it is important to note that learning from experience is further dependent on 'readiness for change' [41, 42: see Appendix 1 Summary of Studies]. Transformation requires assimilation and accommodation of learning from experiences; unless experiences are approached with a mindset of potential to learn and change then it is likely that any new or conflicting meaning / knowledge arising from experience will be rejected. This holds true regardless of the individual's role, as patient, trainee or GP. Approaching this issue from an educational perspective Leykum *et al* [80] propose a similar theory for change with their concept of engaging in 'reciprocal learning'. People need to be willing and interested in learning in the sense of integrating new understanding into their existing conceptualisations rather than rejecting outright new information or taking a tokenistic approach to apparently 'mastering' a way of being at a superficial level without actually believing it is of value.[x]

In the two EI model studies [24, 112) identified as being relevant learning and healthcare delivery were implicitly intertwined as changes in attitude were required for both patients and GPs in order for them to learn and work as a team [24]. The importance of professional ownership of change was identified as an essential mechanism to bring about practice developments [112]. Other studies also support these findings. For example, Henschen *et al* [63] found that integration of medical students into practice was improved and patient support increased when learning about chronic care occurred through engagement in the implementation of a chronic care model for practice. The need for 'knowledge in practice' to be generated through meaningful care provision as a supported trainee was highlighted in other empirical studies [80, 126] as was the potential benefit to patients of increased trainee engagement [6] and many of the other citations included in the synthesis (for full details and references see Appendix 3). Successful learning and successful care were both dependent on good interpersonal relationships in which trust was a foundation.

A few specific constructs of failure were also identified, although often this was perceived to be simply the negative corollaries of success constructs. Dornan *et al* [40] raised concerns about the potential for misapplication of 'safety agendas' to produce risk avoidance (as opposed to risk

management) that would be detrimental to experiential learning in the short term and patient care (as a secondary effect) in the long term. A lack of readiness or willingness to change was identified by Doubouloz *et al* [41, 42] as a cause of difficulties in interactions between patients and practitioners whereas even if there was a breakdown in relationships and interaction then this was potentially surmountable if all involved were willing and able to reflect on and learn from the experience [123]

Overall parallels were found between models of experiential learning for practitioners and patients, with frequent reference in included studies to theories of transformation and socio-cultural processes as mechanisms for improving clinical care.

PATIENTS', TRAINEES' AND GPS' EXPERIENCES AND CONCEPTUALISATIONS OF MULTIMORBIDITY IN PRIMARY CARE

Realities of primary care in the context of multimorbidity

Sixteen citations contained empirical or model-based studies that were specific to multimorbidity in primary care. Brief summaries of the methods and key findings of each of these citations are provided in Table 1. None contained designed interventions. Thirteen were empirical non-interventional studies (two of which produced explanatory models for practice and interactions). Of the remaining three all produced models, one was a literature review and two were theoretical papers.

Loffler *et al* [81] conducted a grounded theory study with patients in order to identify program theories of coping. The patients distinguished between employing emotional coping strategies when they believed 'nothing could be done' versus problem-solving strategies when change was considered a possibility. Proactive behaviour of patients appeared to be key to coping. Mechanisms utilised included maintaining a social role, sustaining meaningfulness in life, choice in the context of support when needed, experience of achieving goals, understanding diseases and being able to prioritise medication. Morris *et al* [89] also conducted interview studies with patients which highlighted similar issues but also demonstrated that patients experienced a cyclical process of disruption – accommodation – disruption – accommodation over time as multimorbidities changed and the impact of these evolved. Accommodation required mechanisms such as engagement with illness management, new strategies to control conditions and symptoms and new learning and understanding to be triggered.

These findings were replicated in many of the other empirical non-interventional studies specific to multimorbidity in primary care. Barnett *et al* [11] challenge the use of single-disease frameworks to configure care, research and education arguing that the extent of need in multimorbidity combined with increased prevalence associated with social deprivation and the impact of mental health disorders in combination with other conditions renders this framework unfit for purpose. Instead they recommend the development of interventions for personalised comprehensive continuity of care. This approach was also the model recommended by the American Geriatrics Society (2012) following a review of the literature, and by the two theoretical papers which contained models [20, 126] the latter of which also drew on communities of practice theory as a means for ongoing professional learning embedded in practice.

Bower *et al* [19] studied the perspectives of GPs and practice nurses. They identify contextual factors which are counterproductive to collaborative working with patients such as externally imposed targets and time pressures. This study identified the 'additive-sequential model' as a mechanism employed to deal with these challenges. In this model GPs manage each concern presented to them in turn until the consultation time ran out, assuming order of presentation was a proxy for priority. Clearly this mechanism reduces the likelihood of a holistic overview of care which is undesirable if relationship-centred negotiation of needs-based learning and need-based care is the overall aim. Moth *et al* [95] identified similar problems, while Luijks *et al* [84] interviewed GPs who reported it was important to them to apply an integrated approach, place medical considerations within the patients' perspectives, share decision-making and responsibility but that achieving these aims was challenging in the face of limited scientific evidence, shortage of time, polypharmacy, and interacting conditions including mental health problems. This study identified the importance of the doctor-patient interpersonal relationship as a mechanism to overcome these challenges. Similar findings were reported by O'Brien *et al* [101] although the GPs in this study also questioned whether many patients had the self-efficacy to pursue self-management in the context of social deprivation and chaotic lives.

Polypharmacy was a recurrent concern in the synthesis data for both patients and GPs. Schuling *et al* [118] describe complex socially constructed reasoning with respect to deprescribing decisions among GPs and pharmacists. Concerns were expressed about the message conveyed to patients if preventative medications were stopped, and the conversations needed to support this as well as concerns about deviating from recognised guidelines even if the practitioner thought the patient was unlikely to gain. Similar findings were also reported by Smith *et al* [123]. Fortin *et al* [49] identified that psychological stress increased with increases in functional impact of morbidities which in turn could impact negatively on patient engagement. Noel *et al* (51, 100] explored patients' views on self-management, identifying multiple examples of problematic interactions with GPs. A study by Townsend [132] also sheds some light on the complexity of these interactions as she identified patient perceptions that GPs were the dispensers of illness capital in situations when patients were concerned about failure to fulfil social roles.

Table 1: EMPIRICAL AND MODEL BASED STUDIES SPECIFIC TO MULTIMORBIDITY IN PRIMARY CARE

STUDY	METHODS	KEY FINDNGS
Loffler 2012 [81]	ENI with model: grounded theory analysis of patient interviews producing a model of coping categories, strategies and outcomes for patients	Identified multifaceted coping strategies among patients (aged 65-85) with multimorbidity (at least three chronic conditions including one musculoskeletal). These patients distinguished between emotional coping (when it is believed nothing can be done to change the situation) and problem-solving focuses of coping which they used when they had expectation of change (social and practical coping). Patients were keen to preserve their autonomy but described emotional oscillation between anxiety and strength. Many of them were making reasoned choices about their use of medication, even when this conflicted with professional advice.
Morris 2011 [89]	ENI with model: longitudinal semi-structured interviews with patients	Interviews were embedded in a RCT aiming to improve self-management support and sought to identify influences on prioritisation and changes in this over time. Patients had at least one chronic condition, but most had more. A theoretical model was produced from thematic analysis of the interviews identifying four sorts of factors which influenced self-management: (1) disruption by conditions (lack of engagement, confusion, being overwhelmed, uncertainty, separation of conditions); (2) accommodation of conditions (continuity from existing illness behaviour/integration with existing practices, control over conditions and symptoms, enough understanding of conditions, confidence); (3) factors influencing the shift from accommodation to disruption (exacerbations, confusion and contradictory information, events, loss of control, medication); (4) factors influencing shift from disruption to accommodation (taking control, links between existing knowledge and experiences, adapting information and practices into new routines, interaction with health care professionals). Prospective use of this model was not part of the study. Patients in the study sought to make new diagnoses minimally disruptive to existing management plans and may have benefited from discussion of their priorities with professionals and/or better information on which to prioritise.
Barnett 2012 [11]	ENI: cross-sectional epidemiological study	This paper presents cross-sectional findings from data from 1751841 people in Scotland registered with a medical practice. They demonstrated that over 40% of all patients had one or more chronic disease and over 20% had multimorbidity, defined as two or more chronic conditions. Multimorbidity increased with age (although the absolute number of people was higher under 65 years) and with social deprivation. Mental health disorders were a significant feature. The authors discuss how these findings challenge the single-disease framework which is used to configure most health care, research and medical education, arguing that this should be complemented by personalised comprehensive continuity of care.
Bower 2011 [19]	ENI: qualitative interviews with GPs and practice nurses	This study identified the tensions primary care professionals experienced between delivering care to meet externally imposed targets and achieving patients' personal agendas. These tensions were felt to be exacerbated in multimorbidity with multiple appointments, time pressures in consultations leading to an additive-sequential decision-making model of care where problems were dealt with by priority until time ran out, challenges in helping patients co-ordinate their care and self-manage conditions. Amongst interviewees there was limited consideration of interactions or synergies between conditions and their management.
Fortin 2006 [49]	ENI: psychiatric symptom questionnaire study	A questionnaire administered to 238 patients comparing psychological distress with a cumulative illness rating scale for severity of multimorbidity found that there was a significant association with increased distress as severity of morbidities increased (although not with a simple count of number of conditions suggestion that functional impact may be relevant). The authors suggest that this adds additional complexity to the management of patients with multimorbidity as psychological distress can impact negatively on patient engagement.
Luijks 2012 [84]	ENI: Group interviews with GPs	Interviews identified themes that were important in the practical experiences of GPs : managing multimorbidity in the face of limited scientific evidence, applying an integrated approach, medical considerations placed into perspective of patients, shared decision-making and responsibility. The outworking of these themes was influenced by the personal relationship between doctor and patient, whether the patient had mental health problems, interacting conditions and practical problems such as shortage of time and polypharmacy.
Moth 2012 [95]	ENI: cross-sectional study	A survey of all GP contacts (during one day per GP) in the central Denmark region was undertaken over a 12 month period. Data was analysed to compare GP workload with multimorbidity. Over 30% of consultations were with patients who had more than one chronic disease and a rise in time consumption and contact burden was associated with this. Diagnoses of depression and dementia led to particularly complex consultations as did additional psychosocial problems. Few contacts were considered appropriate to delegate to other members of the primary care team by the GPs.
Noel 2005 [51]	ENI: focus groups with patients	The study aimed to explore the care needs and preferences of patients with at least two chronic illnesses in primary care. Identified problems included poor levels of function, negative psychological reactions, negative effects on relationships and interference with work or leisure activities. Polypharmacy was also a major concern. Some patients described problematic interactions with professionals and health care systems

		such as feeling ignored or being offered conflicting advice although most expressed appreciation for the care received in primary care settings. Patients were willing to engage in self-management and the use of technology but did not want this to replace human contact. Support from professionals other than doctors was also considered acceptable if complementary rather than replacing doctor consultations.
Noel 2007 [100]	ENI: cross-sectional survey	422/720 patients were returned surveys following stratified non-proportional sampling to identify their self-management learning needs and willingness to see non-physician providers (comparing patient with and without multimorbidity). Patients with multimorbidity were significantly more likely to express willingness to learn self-management techniques than those with a single chronic condition, and a higher percentage of those with multimorbidity were willing to see non-physician providers.
O'Brien 2011 [101]	ENI: qualitative study of GPs and practice nurses	Interviews were conducted with primary care professionals in four practices with a high percentate of patients living in deprived areas of Scotland. Practitioners described their management of multimorbidity as requiring them to address multiple psychological, social and health problems, experiencing an 'endless struggle' with patients trying to manage illness in the context of chaotic lives and few resources, personal consequences for some professionals and a desire to pursue holistic approaches. The authors conclude that data confirms the presence of an inverse care law in the context of multimorbidity. Professionals were concerned that these patients lacked the self-efficacy to pursue self-management and thought there was a need for health care delivery systems to be redesigned.
Schuling 2012 [118]	ENI: qualitative focus groups with GPs	GPs were interviewed about stopping medications due to the risk of adverse effects from polypharmacy. GPs were able to delineate differences between symptomatic and preventative medication but found the latter more difficult to deprescribe in elderly patients as they were concerned about how patients might interpret this e.g. being given up on, requiring a conversation about life expectancy versus quality, contradicting guidelines, patients not unhappy with polypharmacy. These GPs were reluctant to explore these issues with patients and wanted decision support in trying to deal with several guidelines relevant to an individual with multimorbidity. Further training in shared decision-making seemed to be needed.
Smith 2010 [123]	ENI: qualitative focus groups with GPs and pharmacists	GPs and pharmacists identified problems with health systems in managing multimorbidity which included: lack of time, inter-professional communication difficulties and fragmentation of care. Personal issues also arose for these clinicians with respect to roles, clinical uncertainty, avoidance, patient concerns and potential management solutions. These findings suggest any intervention in multimorbidity needs to address the concerns of professionals in order to bring about any improvement.
Townsend 2012 [132]	ENI: patient interviews using Bourdieu's concepts for analysis	The analysis of interviews with 23 people with 4 or more chronic illnesses in this study highlights how broader cultural structures became part of individuals' narratives of their illness with for example GPs perceived to be the dispenser of capital (e.g. legitimising the sick role). There was evidence of on-going negation between structure and agency which underpinned illness actions such as help-seeking and self-managing. Patients experienced losses of previously taken for granted activities, disrupted family relationships, and awareness of a sense that they were not fulfilling societal expectations. Many adopted strategies such as stoicism to try and regain control and avoid being judged as 'failures'
AGS 2012 [4]	LR with model	Following a literature review (integrating searches for patient preferences, interpreting evidence/prognosis/clinical feasibility, optimising therapies and care plans) the expert panel of the AGS suggested there was a need to develop guiding principles for the care of older adults with multimorbidity. Their model approach recommends first focusing on the each patient's primary concern before either addressing a specific aspect of care in negotiation with the patient or reviewing the whole care plan. Consideration of prognosis, interactions within and among conditions and treatments, benefit and harm and regular reassessment should all form part of the negotiation. The model was not tested in practice.
Boyd 2010 [20]	TO with model	A theoretical review of the implications of multimorbidity for health system design and future research, this paper draws heavily on the 'Chronic Care Model' (Wagner) with its emphasis on a patient-centred approach. The model itself appears to be used in practice but no formal evaluation is reported. It incorporates consideration of all levels of healthcare organisation from individual doctor-patient interactions to system wide organisation.
Soubhi 2010 [126]	TO with model	Starting from the assertion that management of multimorbidity form chronic disease cannot be achieved solely through disease-specific approaches this paper develops a theoretical model of care which draws on communities of practice theory to develop shared learning between patients, their families and professionals. The model was not tested in practice.

Conceptualisations of success and failure

In this section we outline the key constructs of success and of failure with respect to healthcare delivery and experiential learning which were identified in the synthesis. Fully referenced details of the literature sources from which these are drawn are included in Appendix 3 and the citation list at the end of this report.

We have identified above how different people (patients, trainees and GPs) perceive the challenges of multimorbidity and the social interactions in which they engage to attempt to address these issues. Conceptualisations of success for both patients and trainees (i.e. good care for patients, learning to give good care for trainees) were found to be socially constructed and dependent on positive relationships, trust and support from others including doctors .

Success in the form of high quality care was subject to flexible and changeable definitions which went beyond clinical markers of disease, or quality markers in current healthcare policy.

Much of the synthesis data was dedicated to exposing the depth and breadth of angst related to the 'social problem)s)' of multimorbidity. For example, Boyd [20] argues that there is a failure of healthcare to systematically match patients' needs to best therapeutic practices (reflecting challenges of creating an integrated clinical plan), failure through approaches that only look at one factor or dimension and may rely on conventional analytic techniques, failure in care that is fragmented, incomplete, inefficient and ineffective, preventable inpatient admissions or complications, failure of receiving services with little benefit and high risks of adverse events [20].

Within the data much of what could be identified as conceptualisations of failure related to quantifiable aspects of care. It is difficult to know whether this simply reflects the value of tools currently available to assess effectiveness and efficiency of learning and care in the context of multimorbidity or whether success may be expressed in qualitative terms while failure may be conceptualised according to clinical biomarkers and other quantifiable measures.

Key constructs of success for healthcare delivery in the context of multimorbidity in primary care included collaborative working practices, holistic and transparent goals which were developed through negotiation, integration of medical and experiential knowledge regarding diseases and impact, professional sharing of best practice, transformative learning through trusted relationships between patients and practitioners to enable self-management.

Key constructs of success for experiential learning in workplaces in the context of multimorbidity in primary care included very similar ideas: learning to engage in and benefiting from collaborative working reciprocal learning, viewing learning as a shared social process, learning from direct interaction with patients, a supportive environment for the appropriate mix of responsibility, challenge and scaffolding to permit a safe but legitimate role in practice all featured as did the importance of physical space to allow interactions between patients and trainees. Both patients and practitioners needed to learn how to make personalised trade-offs between risks and benefits in multimorbidity and to manage competing priorities which could change over time.

Key constructs of failure for healthcare delivery included repeated / prolonged hospital admissions and a reluctance on the part of clinicians to look beyond biomedical markers in addition to the negative corollaries of the described constructs of success above. Notable for its absence in the

synthesis was a positive construct about clinical biomarkers. This suggests that a focus on clinical biomarkers alone is never sufficient, and may sometimes be unnecessary.

Key constructs of failure for experiential learning included contexts which reduced students and patients to passive roles, negative workplace cultures and lack of exposure to multimorbidity with excessive focus on single-disease frameworks and overreliance on guidelines often not developed on evidence applicable to patients with multimorbidity in primary care.

Implementation and outcomes

Within the synthesis a number of proposed models for learning, care delivery or both were identified. None of the citations contained full implementation studies of these models but important aspects of implementation and intended outcomes within these studies could be identified. A summary of this analysis is provided in Table 2, Potential Theories.

As Table 2 demonstrates, more is known about the intended outcomes of proposed models and interventions than about what happens in reality. There was some limited evidence of naturally occurring mechanisms and contextual constraints which could lead to undesirable outcomes for both patients (dissatisfaction with care of varying suitability for them) and trainees (development and replication of naturally occurring practices which would not serve patients well). However, concerns were also raised about the potential risks of interventions which disrupted functional practice. These are issues which require further research to explore.

Table 2 POTENTIAL THEORIES		Implementation aspects	Intended outcomes
…models for learning	Educational alliances [138]	• Need to trigger interpersonal connections between trainee and supervisor	• Educational alliance – defined as partnership producing just the right amount of responsibility – a balance between support and challenge with professional acting as safety net for patient and trainee
	Beacon practices [67]	• Need to trigger inter-practice links<br>• Contextual infrastructure required	• Collaborative and extended roles in primary care for professionals
	Communities of practice [21, 80, 105, 126, 127, 105]	• Need to trigger genuine team-working between patients, trainees and professionals<br>• Trust required between all and relationship building a crucial mechanism for interventions to work<br>• Studies of actual working practices including during interventions needed<br>• Any intervention needs to focus not just on education or decision-support for individuals but also the dynamic system in which they are situated	• Harnessing of emergent learning from practice and experience<br>• Dynamic approach to care aligned to shared goals<br>• Able to capture in-practice learning and innovation to further develop and improve outcomes (emergent learning)<br>• Reciprocal learning and sharing of best practice through system adjustments to support this<br>• Development of communities of practice
	ExBL [39, 40]	• Need to trigger 'virtuous learning cycles' – participation, balance of support and challenge, graded responsibilities	• Practical competence<br>• State of mind conducive to practice (confidence, motivation, sense of professional identity)
	Breakdowns [122]	• When a breakdown (a situation where a person is not achieving expected effectiveness) occurs then interventions must trigger reflective learning and an effective response from others<br>• Contextual factors: patient engagement, responsibility matched to authority, tools matched to task, information resources matched to need, values shared between co-participants, expectations matched to capacity	• Constructive learning for future practice
	Developmental space [134]	• Creation of developmental space to permit learning and development of professional identity – space created through workplace context, personal and professional interactions and emotions such as feeling respected and confident	• Mindful learning and development
…models for care delivery	Guided Care [87]	• Increased staff resources for patient support	• Increased satisfaction with communication and increased knowledge of patient clinical characteristics
	Patient Centred Medical Home [75, 80]	• Need to trigger social, psychological and physical assessment<br>• Need to trigger active patient and professional participation	• Holistic care developed through patient and professional collaboration
	CARE approach [14]	• Need to trigger connections between patients and professionals	• Holistic assessment, appropriate responses and patient empowerment
	Chronic Illness Care Plans [112]	• Need to trigger holistic assessment – requires professionals rethinking their roles	• Individualised care plans
	The Chronic Care Model [20, 33, 80, 137]	• Need to trigger a patient centred approach including relational and management continuity<br>• Need to trigger reciprocal learning<br>• Contextual factors are community resources and policies	• Holistic care shared between patient and provider<br>• Sharing of best practice
	Self-management support five A's [55]	• Need to trigger assessment, appropriate advice, agreement of goals, assistance in behavioural change, and monitoring<br>• Context 'self-management' of some sort is inevitable as clinicians are only present for a fraction of a patient's life	• Personal action plans for patients and increased purposeful self-management
	Shared decision-making [77,79, 116]	• Need to trigger desire for patient involvement (varies according to reason for encounter)<br>• Mechanism – education of health professionals about sharing decisions alongside patient mediated interventions	• Appropriate shared decision making
…models for both	Transformative learning [10, 41, 42]	• Triggers are lived experiences combined with readiness for change leading to critical reflection, restructuring of meanings and development of new meanings	• New rules, ways or guidelines, new behaviours, feelings, beliefs, perspectives, identity<br>• Learning about self and chronic illness in an iterative and continually changing manner
	Response shift [10]	• Triggers are lived experiences combined with readiness for change leading to critical reflection, restructuring of meanings and development of new meanings	• New rules, ways or guidelines, new behaviours, feelings, beliefs, perspectives, identity
	Education centred medical home [63]	• Need to trigger legitimate participation of trainees in continuity of patient care	• Increased patient support<br>• Practice based learning experiences

REFINEMENTS TO ORIGINAL PROGRAMME THEORIES

Following completion of the synthesis a refined programme theory for producing relationship-centred negotiation of needs-based learning and needs-based care runs as follows:

1. How people (patients, trainees and GPs) think about success and failure in multimorbidity matters because these concepts will influence:
    a. Their priorities for healthcare in multimorbidity
    b. Their goals for people with multimorbidity
    c. How they interact with each other, which in turn will influence:
        i. The care people receive now
        ii. How decisions are made in the face of uncertainty
        iii. How future doctors learn to interact with people with multimorbidity
        iv. The experiential learning of patients, trainees and GPs i.e. knowledge construction and meaning-making about multimorbidity, which then influences new interactions and further learning (in non-linear cyclical processes)

    All the above will influence relationship-centred negotiation of needs-based learning and needs-based care, including whether or not there is good alignment between personal expectations and lived experiences. Dissonance between these is likely to produce emotional distress as well as present cognitive challenges.

2. Doctors must deliver healthcare and provide constructive learning opportunities for future doctors through workplace-based experiential learning which balances individual needs of learners with challenge, support and feedback. It does not make sense to develop isolated idealised models of delivery of care and of education when both have to happen in the same place, at the same time, with the same people.

3. Real world practice is an important contextual element. Without understanding the socio-cultural context of 1. it is not possible to develop a sustainable workforce to deliver needs-based healthcare as we would be seeking to offer solutions without understanding the influences of human actions in any intervention or what specific problems are in current practice.

4. We know that specific disease-based learning, care delivery and research is inadequate: index conditions defined by practitioners according to biomedical models may not match patient perspectives or account for functional impact / proliferation of guidance developed from evidence that does not include the patient in front of a given doctor most of the time / lack of community-based medical education. To be expert in generic skills such as decision-making in the face of uncertainty, for example working beyond guidelines, requires one to learn these skills through multiple exposures in different contexts and with the support of others to make sense of experiences and learning.

5. Meaning influences behaviours, negotiations between patients, trainees and GPs, roles and responsibilities, decision-making and clinical judgements, all of which, in turn, will impact on whether needs-based and relationship-centred outcomes are achieved.

6. Interactivity is important because once we know how and why success and failure are conceptualised in multimorbidity, where there is an absence of cure, and the effect of this on evolution of knowledge, meaning and practice, we can then develop programme theories designed

to inform the development of a sustainable, appropriately equipped, workforce that can deliver holistic patient-centred care for this growing population.

Table 3 (below) summarises the overall findings of this synthesis identifying how multiple socially constructed contextual elements can cultivate social mechanisms to increase the likelihood of achieving relationship-centred negotiation of needs-based learning and need-based care through increasingly the probability of outcomes that together lead to this goal.

This synthesis focused on the concurrency of workplace-based experiential learning with healthcare delivery in the context of multimorbidity in primary care. The professional and public populations involved in this context can be diverse and wide-ranging. Therefore, the synthesis was narrowed by specifically looking at three populations: patients with multimorbidity, trainees seeking to become doctors (i.e. undergraduate medical students and postgraduate trainees) and doctors who, as fully qualified GPs, are responsible for both workplace supervision of trainees and the care of patients within primary care settings.

For these three populations the synthesis has identified important elements of the contexts in which they interact for achieving relationship-centred negotiation of needs-based learning together with needs based care. These are space (physical, cognitive, emotional) and time to critically reflect and participate in reciprocal learning and collective sense-making, organisational flexibility with respect to tailoring of care (permitting patient and physician choice rather than a narrow view of compliance/adherence), resources including time for learning and healthcare activities and multiple options for continuity (as shown in Table 3).

Within these contexts, assuming the desired contextual elements are present and/or cultivated (e.g. through organisational culture, supporting structures and external influences such as healthcare and educational institutions) the main socially constructed mechanisms for achieving relationship-centred negotiation of needs-based learning together with needs-based care are:

- Self-efficacy from a belief in potential transformation
- Trade-off decision-making in the face of uncertainty versus rigidity of guideline application
- Co-construction of success and failure: balance of challenge to foster self-efficacy and support
- Genuine collaboration: paradigm of learning together in partnership, and team working with shared understanding of identities and roles

This overarching outcome is also itself made up of component parts. For example, individual and social knowledge construction and sense-making, transformative learning (can be positive / constructive or negative / destructive, Shared decision-making and management according to consensus priorities, shared (graded) responsibilities, and personalised goals with continuous remodelling of care to meet these as these evolve.

<table>
<tr><th colspan="3">Table 3 PRODUCTIVE CONCURRENCY: WORKPLACE-BASED LEARNING AND SUSTAINABLE PATIENT CARE</th></tr>
<tr><th>Context</th><th>Social Mechanisms</th><th>Outcomes</th></tr>
<tr><td rowspan="4">Space (physical, cognitive, emotional) and time to critically reflect and participate in reciprocal learning and collective sense-making

Organisational flexibility with respect to tailoring of care (permitting patient and physician choice rather than a narrow view of compliance/adherence)

Resources including time for learning and healthcare activities

Multiple options for continuity including relational (longitundinal) and / or temporal (informational) management

Leadership to create culture that favours trust, openness and innovation</td><td>Self-efficacy from a belief in potential transformation</td><td rowspan="4">Relationship-centred negotiation of needs-based learning and need-based care which encompasses:

Individual and social knowledge construction and sense-making

Transformative learning (can be positive / constructive or negative / destructive

Shared decision-making and management according to consensus priorities

Shared (graded) responsibilities

Personalised goals with continuous remodelling of care to meet these as these evolve (e.g. through non-linear trajectories of illness including: Stabilising the condition to get patients back to living their lives, support through monitoring and review, timely intervention by appropriate service when things go wrong, providing choice and support towards the end of life)

Development of self-efficacy as a common goal through learning to cope in different ways: social (maintaining a role, autonomy and having a can-do approach), emotional (when it is believed there is nothing to be done to change the situation, positive approach to life despite adversity) and practical (expectation of change, focused on control of disease and therapies) [81]

Learning to live with expectation of unpredictability and uncertainty, including being able to handle and admit feelings of ‘not knowing what to do’ (trainees, practitioners and patients)</td></tr>
<tr><td>Trade-off decision-making in the face of uncertainty v rigidity of guideline application</td></tr>
<tr><td>Co-construction of success and failure: balance of challenge and support</td></tr>
<tr><td>Genuine collaboration: paradigm of learning together in partnership, and team working with shared understanding of identities and roles</td></tr>
</table>

A MODEL OF SPECIFIC PROGRAM ELEMENTS TO ACHIEVE DESIRED OUTCOMES

The emergent model presented in Figure 1, originates from a process of mapping concepts, either arising from empirical findings, or the use of socio-cultural, experiential learning and transformation theories, from the synthesis data. The model provides a visual representation of the mid-range theories developed and described above. It is a model of principles with respect to the context and mechanisms which are more likely to lead to the desired outcome of relationship-centred negotiation of needs-based learning and need-based care.

The 'artistic' representation is deliberate; to capture the essence of social interactions as 'learning' triggers and experience as non-linear processes with the potential for recurrence and movement in multiple directions as individuals / groups seek to make sense and understand the complexities of multimorbidity.

Experiential learning should provide a mechanism for trainees to put their knowledge into practice. From a patient's perspective experiential learning related to multimorbidity is not a choice, but simply the means by which they come to understand their ever changing health and illnesses. Strategies such as self-directed care and self-management as well as shared-decision making draw on this experience, seeking to integrate it with professional knowledge and support. For both patient and trainee, therefore, experience is supposed to lead to greater knowledge and understanding about how best to manage multimorbidity.

Underlying these ideas are socio-cultural perspectives on experiential learning theories. Real life experience influences knowledge construction and sense-making through the impact of interpersonal interactions on cognition, emotions and social behaviours. Everyone learns (something) from experience due to intrinsic human desires to engage in sense-making. Whether the emergent learning is constructive or not will be subject to contextual influences as well as interpersonal interactions. This means that learning from experience is, by definition, a transformative process. If you could be left unchanged, in even the smallest detail, by an experience then it would be questionable that you had 'learned'.

At the centre of our model patients, trainees and GPs are represented in 'free-floating' circles around which the most pertinent concerns from the perspectives of each of these groups can be found. These revolving circles are intended to represent the multiple possibilities for how interactions and experience can shape sense-making, learning and practice in the context of resources available and organisational support (or lack thereof). In the context of primary care these are the mechanisms which interplay to produce experiential expertise, that is, personal and social knowledge construction and sense-making among patients, trainees and GPs. The outcomes consequential to these mechanisms are fluid, and subject to change as the mechanisms can operate both 'positively' and 'negatively'. Important contextual elements (things likely to create the preconditions for human behaviours) for these interactions are the supportive resources available and organisational culture.

Nonetheless to produce our primary outcome of interest there are identifiable contextual elements and 'ways of being and becoming' which will increase positivity in the underlying mechanism of social interactions. The outer loop of the model represents a process of transformative learning for all three groups which is likely to be both cyclical and non-linear. This outlines the proposed necessary steps by which relationship-centred negotiation of needs-based learning and need-based

care are achieved. The inner loop suggests mechanisms which need to be triggered by the context and interactions of the groups in order to precipitate these steps. These mechanisms are not claimed to exclusively lead to the desired outcomes but in recognition of semi-predictable patterns (demi-regularities) in human choices, but may be necessary elements of the processes which must be in operation as people interact with each other and the resources available to them.

As suggested by theories of transformation, learning, meaning and identity are interlinked. Therefore, to maximise learning a trainee needs to be (and feel) recognised as a legitimate part of the practice activities. The additional gains to be made through expert support can also be interpreted through theories such as Vygotsky's zone of proximal development[xi] – that is, learning is still seen as critically dependent on interaction with others who can extend the meaning-making and knowledge construction beyond that which one might reach independently. The model can, therefore be considered to have multiple layers of mechanisms: social interactions which are naturally occurring, elements of collective and collaborative approaches to the challenges of multimorbidity, and transformative learning. When these mechanisms are functioning constructively (positively) then it is possible for relationship-centred negotiation of needs-based learning and need-based care to result. Variety in any of these elements can influence individual and collective learning arising from social interactions. It has been well established that context and the potential for genuine participation are particularly important for potentiating learning in workplace-based experiences[xii]. Equally important is the need to distinguish intended learning in ideal circumstances from experience in practice when other factors may lead to unpredictable consequences.

MODEL (designed in collaboration with Lucy Robertson)

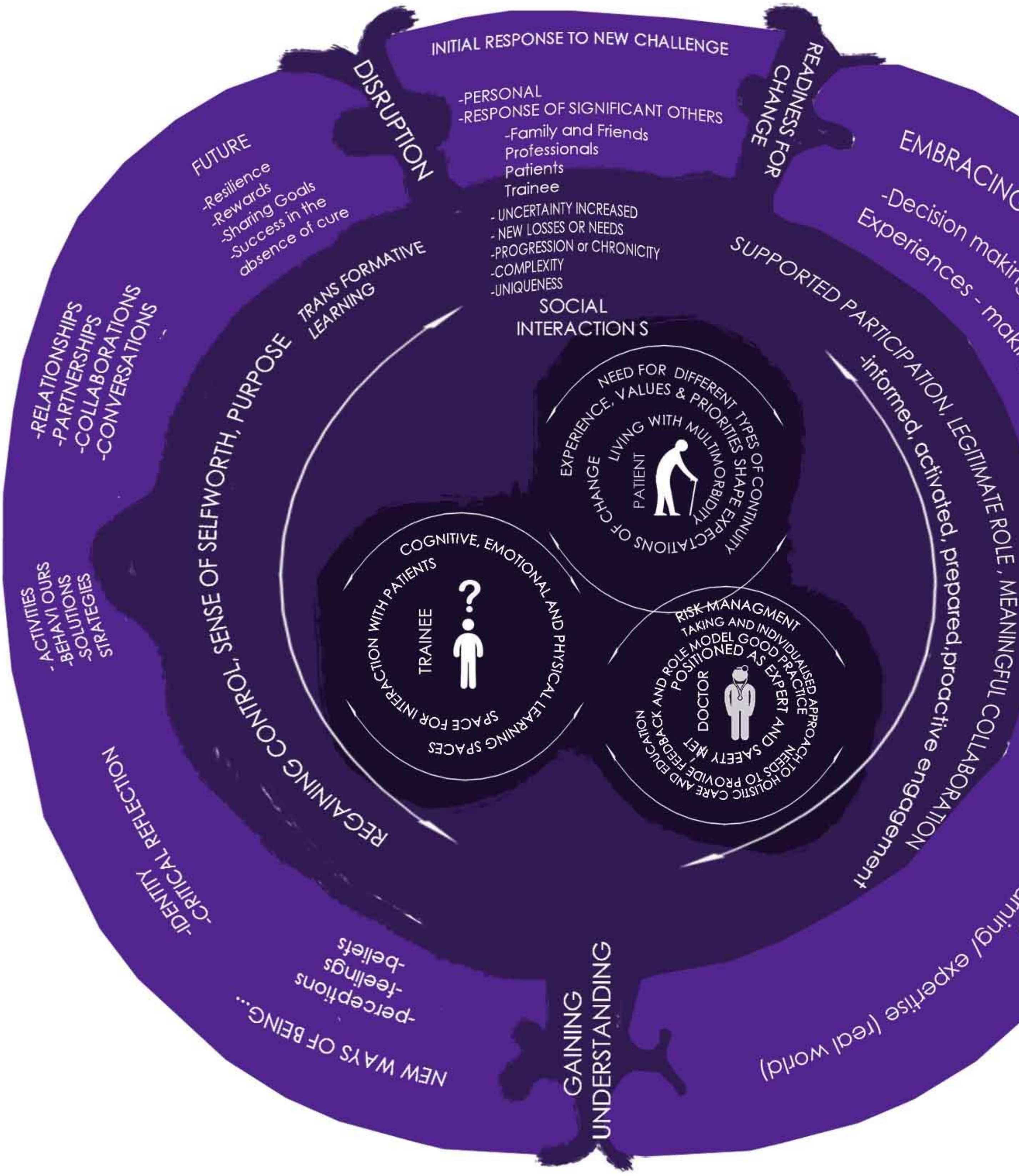

DISCUSSION

Theorising that identifying how success and failure were conceptualised in the absence of cure would be essential to understanding how and why interactions shaped these concurrent activities, and therefore, relationship-centred negotiation of needs-based learning and needs-based care, using realist synthesis we have sought to explicitly account for the influence of interactions between patients, trainees and GPs engaged concurrently in workplace-based experiential learning and healthcare provision related to multimorbidity in primary care. Simply having patients with multimorbidity, trainees and GPs in primary care in each others' presence means *something will happen*. Much of the empirical evidence included in this review can be considered as being derived from such a *natural experiment* that we have studied with respect to understanding context, mechanisms and outcomes of social interaction.

Parallels were identified between models of experiential learning for practitioners and patients, with frequent reference in included studies to theories of transformation and socio-cultural processes as mechanisms for improving clinical care. However, while there is congruence between existing models of experiential learning and patient engagement in healthcare in the context of multimorbidity recognition of the concurrency of these activities has been lacking in the academic literature. In addition, most models were untested in practice. Given that concurrency is the *de facto* non-optional occurrence in practice these are significant oversights. Concurrency cannot be ignored as it affects practice now and in the future through mechanisms of experiential and transformative learning for patients, trainees and GPs. Our realist synthesis of the existing literature has enabled the development of a theoretical model (program theory) to explain why social interactions are important mechanisms and how these might be optimised to produce relationship based negotiation of needs-based learning together with needs-based care in the context of multimorbidity in primary care.

The potential for non-linear bi-directional transitions between different parts of the model we present (whether applied to patients, trainees or GPs) can be explained by social interactions with each other as well as levels of belief or non-belief in the potential for (positive) change or transformation. Readiness for, and expectation of, change are essential elements if transformative learning is to arise from lived experiences. These elements need to be combined with a context that permits change by allowing all participants in the process the time to engage in relational working, shared responsibilities and personalised choices and risk management. Only then can any specific intervention to improve practice become embedded and replicated through social interactions and experiential learning.

Interactivity is particularly important in the context of multimorbidity in primary care when trainees are present because practice is socially constructed and the interactions of trainees will shape their future practice as much as the interactions of patients and GPs shapes future behaviours. If transformation is conceptualised as a process of learning about oneself and chronic illnesses in an iterative and continually changing manner then this applies to both patients and trainees. Both require a 'readiness to change'.

If everyone is conceptualised as a learner and part of a team with the common goal of continually improving healthcare delivery for people with multimorbidity then success and failure can be conceptualised as achieving the evolving goals of that team and learning gained in the process of

caring for individuals can be transferred through negotiation with others. This renders fixed outcomes of success as inappropriate. Aside from anything else the influence of (unscripted) conversations [70] will shape learning and care. Patients, trainees and GPs are all likely to experience cycles of disruption and accommodation in their learning patterns during the trajectories of multimorbidity. This will inevitably lead to ongoing reconsiderations of their definitions of success and failure in the absence of cure. Social interactions provide the mechanisms by which this can be turned into shared learning and care based on needs together with shared goals, rather than disjointed practice occurring secondary to the development of assumptions within any one group about the others.

This is not to suggest that ‘transformation’ is an automatically utopian process, nor will it always produce constructive outcomes. Rather a fresh consideration of transformative learning, defined as accepting that life’s experiences act as a stimulant to sense-making which will influence knowledge construction, assimilation (new beliefs, rather than simply tokenistic mastery to meet the demands of others) and interpersonal interactions is important for understanding how social interactions shape whether interventions stand or fall in everyday practice.

These conclusions resonate with Pawson’s VICTORE model[ix] of complex interventions within complex social systems. The choices made to act (or not) shape social interactions among patients, trainees and GPs. The concept of experiential learning is underpinned by constructionism: different people perceive different versions of reality (different theorists may subscribe to a realist view, that despite this there is ‘truth’ although we can only partially know it, or a constructivist view that there is no ‘truth’[xiii]). Important common threads in the many theories of experiential learning include the following. (1) Learning is viewed as a lifelong continuum. (2) Direct personal experience is important for understanding, and understanding is potentiated by active roles and engagement, with appropriate support and encouragement to think critically about experiences. (3) Learners need to be able to relate new experiences to prior ones in order for learning to develop and meaning to be attached to experience. (4) In workplaces multiple experiences are gradually built into complex organised abstractions recognised as knowledge. This knowledge is likely to draw on social ‘know-how’ and ‘how things are done around here’ as much as formalised academic sources. This process relies on assimilation of new information and accommodation of this; rearranging prior understanding in the light of new experiences. It is important, therefore, to consider not just if someone ‘learns’ the ‘correct’ outcome from an experience but to consider their reasoning if their learning is to be productive in the future. (5) It is much easier when faced with conflicting information or experiences to chose to reject one or other form than to work to find an explanation which is nuanced enough to account for both. (6) Learning from experience is also affected by perceived relevance of that experience to current goals. (7) Viewed as described in statements (1)-(6) experiential learning has an implementation chain which extends historically for any individual to way before their engagement with a specific intervention designed to change or optimise current and future behaviour.

Strengths and limitations

While the size and complexity of this synthesis has been challenging, it was necessary to design it in this way to reflect and understand the complexities of interactions between patients, trainees and

GPs. Literature syntheses are inherently dependent on primary studies and our review identifies gaps in the literature. This is, however, important for the prioritisation of further research.

In conducting this synthesis, review of potential evidence may not have been exhaustive. However, we believe that we have developed a suitably robust search strategy to identify the most relevant literature and this combined with the iterative approach described in the study protocol[viii] and methods (above) has allowed us to purposively select evidence to inform concurrency as well as concepts of success and failure in the absence of cure.

A realist synthesis seeks to analyse evidence in order to understand interactions between context, mechanisms and outcomes. Given the focus on reasoning, preferences, norms and collective beliefs (conceptualisations) in this synthesis it was logical to draw on literature in which the participants – patients, GPs and trainees outline their theories and angst about multimorbidity and how learning and care function in this context as well as more traditional sources of empirical and theoretical evidence. It can be seen from the descriptive summary of the types of evidence used in this synthesis that robust research evaluating the implementation of specific interventions to improve learning, and hence sustainable healthcare in the context of rising multimorbidity is needed. We also accept that the literature may, or may not reflect 'real world practices' of trainees, patients and GPs due to a paucity of studies using methods able to record and critically analyse concurrency in action. This is an example of a specific gap which further research could address.

We recognise that our model, as a candidate theory, cannot be considered definitive until it is tested and refined in the 'real world'. What we are proposing here is a model of elements for consideration by designers of and participants in workplace-based learning in the context of multimorbidity in primary care. The program theories integrated in this model developed through specific study of concurrent workplace-based learning and healthcare delivery in the context of multimorbidity in primary care. Nonetheless, this model may have wider applicability as a framework in clinical workplaces where learning and care delivery occur simultaneously.

As described in the protocol for this work this synthesis has focused on experiential learning and doctors, specifically looking at the triadic relationships between patients, trainees and GPs. We acknowledge that an additional layer of complexity exists in clinical practice as other professionals plus the significant others such as family and friends of patients will also interact with and influence our three groups.

Relationship of findings to other literature

The ultimate goal of medical education is to create doctors (and other professionals) with effective behaviours in daily clinical practice for the best patient care. Experience is a crucial element of this learning process. A Cochrane review of interventions in multimorbidity only found 10 empirical studies of complex interventions of which eight focused on multimorbidity (the other two were on specific co morbidities). Six of these suggested changes in organisation of care delivery and four were patient orientated [124]. This review was focusing on effectiveness and so sought outcomes including any validated means of physical, mental or psychosocial health status including quality of life outcomes, well-being and measures of disability or functional status, measures of provider-patient behaviour including medication adherence, utilisation of health services and acceptability of services and costs. The authors concluded these outcomes were difficult to improve in

multimorbidity and suggested that focusing on specific risk factors or functional difficulties might be more effective. There were no identified economic analyses in their review although changing prescribing could potentially alter cost. Overall there was a paucity of intervention research [124].

Many of the concerns and failures identified in this synthesis resonate with the work of Sinnott *et al* who conducted a metaethnographic synthesis of primary studies using qualitative methods to explore GP's perceptions of clinical management of multimorbidity.[xiv] They found four areas of specific difficulty: disorganisation and fragmentation of healthcare; inadequacy of guidelines and evidence-bases; challenges in delivering patient-centred care and barriers to shared decision making. Reaching shared understanding of goals (when possible in advance of any crisis) is crucially important as it has been suggested that goal divergence between patients, caregivers and professionals tends to occur when patients are more medically unstable.[xv]

Worldwide both undergraduate and postgraduate medical education strategies have traditionally included opportunities for experiential learning in workplaces. Historically this took the form of apprenticeship models pre-Flexner, and these are increasingly being returned to albeit with increased support and scaffolding for learning, alongside greater demarcation of learning from service delivery. This is, perhaps in recognition that although Flexner's emphasis on science was, and still is important, much of the art of medicine is learned through real world practice and experience. Given that experiential learning has been the time-honoured routine in postgraduate medicine, it, has perhaps been taken for granted and therefore not been subject to critical analysis. Within the academic literature most scholarship exists in relation to some specific interventions such as structural support around particular programmes of training.[xvi] Such apprenticeship approaches arose out of practical traditions rather than organised theory leading to designed implementation.

The undergraduate field has been a more active arena. A systematic review of the evidence for experience in clinical and community settings to contribute to early medical education was updated in 2010. The original and update together covered the period 1992-2008.[xvii] Early medical education was defined as the first two years of undergraduate medical degrees in the UK, or the equivalent elsewhere. The use of experiential learning in workplaces has become commonplace at this stage of training, and usually incorporates time spent in general practice as well as elsewhere.[xviii] This means that these initial years will set the scene and lay foundations for potential future learning (or not) from further workplace-based experiences. Findings of the reviews demonstrated several themes in the evidence base. The literature suggested that early experiences could help learning of appropriate attitudes and orientate students towards societal need. Students were seen to learn about patients and community aspects of health and disease in addition to basic communication skills. Perhaps not surprisingly predicting outcomes from any given experience remained challenging, and often varied among individuals even when experiences were similar. Over the time period reviewed there was strong representation in the literature on motivation and quality of learning, career choice, and professional roles, all of which the evidence suggested could be influenced positively through early experiential learning opportunities in workplaces. Fewer outcomes related to communication skills were identified but these were also positive. In the review update (2002-8) period a new trend towards using experiential learning for the primary purpose of ensuring students understood patient perspectives emerged. This might reflect wider concerns within healthcare about the position of patients as active partners and collaborators in illness management rather

than passive recipients, or reflect concerns about reports of unnecessary detachment and cynicism developing as students progress through their training.

Others have also studied the role of real patients in learning once the clinical clerkship years are reached. Studies report student trainees identifying both affective and cognitive enhancements. Affective changes include increases in confidence, motivation, satisfaction and sense of professional identity.[xix] Cognitive changes included gaining appreciation of complexity as well as making learning more meaningful and focused with students reporting the acquisition of complex skills and knowledge. In a study of year 5 students van der Zwet *et al* found that students reported great variance in the learning opportunities and support on offer when working in general practice with supervision, patient mix and independence all identified as contextual influences.[xx] A further study by these authors [134] reported on the importance of 'developmental space' for learning. This space describes a socio-cultural conceptualisation of the intertwining of workplace context, personal and professional interactions and individual affective states to produce 'space' for 'development'. Further research from a socio-cultural perspective to explore workplace learning in medical education was recommended following this study as interactivity has been relatively ignored previously. This realist review begins to address the challenge set by van der Zwet *et al*, considering not only interactivity within education but between education and care in workplaces.

Steven *et al* recently studied how undergraduate medical students learn from real patients in practice settings through analysis of audio-diaries. These demonstrated that participation in the practices of workplaces including direct patient care was essential for high yield learning but that education was not always coupled with patient care.[xxi] This had a direct impact on the nature and quality of learning with expert supported dialogue linking learning opportunities to direct patient care producing optimal results. This further emphasises the importance of accounting for concurrency in workplace-based experiential learning. Van der Zwet *et al* have also begun exploring GP perspectives on their interactions with clerkship students.[xxii] Their work demonstrates that within each clerkship there is a trajectory of relational development that varies according to first impressions of student trainees, the development of dialogue with them and individual conceptions of good medical practices. Werne *et al*'s review [138] of the literature on GPs as workplace supervisors in postgraduate clinical education found that a key skill of the supervisor was to intertwine clinical and educational activities. This combined with the formation of relationships with learners (educational alliances) was necessary as a foundation for learning. As has been found with earlier training these learners also needed a balance of challenge and support within an environment that gave them opportunity to put learning into practice and so develop their understanding and roles.

Human interactions are key to implementing the model [6, 40]. Emergence of high quality practice (applied knowledge, skills, attitudes and behaviours) should come from workplace-based learning opportunities, through improved learning opportunities and clinical outcomes from self-directed learning and self-management. To achieve this requires an understanding of how people think about success and failure in the absence of cure and how this will affect their interactions.

Further research needed

We specifically sought literature related to symptomatic chronic conditions with no current definitive cure in the patient trajectory. The existing literature in this area suggests that further work

drawing on theories of transformation and socio-cultural processes should be pursued to understand and optimise outcomes for trainees, patients and GPs. The emergent model of concurrency for medical education and healthcare delivery presented as a visual representation of our findings together with the explanatory refinements to our original program theories offers a basis to test 'what works, for whom, in what circumstances and why?' in future interventions. This should inform the direction and design of research-based interventions to produce a sustainable medical workforce equipped to provide multimorbidity care.

Smith *et al* [125] offer a 'consensus of experts' for how future interventions to improve patients with multimorbidity should be designed and evaluated. Their structure is useful, particularly in the research context, as it recognises the value of more participatory and collaborative working studies. We realise that experimental designs are favoured, for example, in the MRC recommendations for developing and evaluating complex interventions.[xxiii] The almost total absence of experimental study designs to evaluate either designed interventions for concurrent experiential learning or healthcare delivery in the context of multimorbidity or studies of the impact of other interventions on a naturally occurring state of concurrency between workplace-based experiential learning and healthcare delivery demonstrates this is an area in which further research is needed. Without such approaches also considering the social and experiential learning of professionals there is, however, still as risk studies could still fail to account for the 'messiness' of real world practice. In turn this risks failure of transfer or failure to achieve the same outcomes in routine practice.

We also agree with the MRC advice that process evaluation should not be considered an adequate proxy for outcome evaluation. However, nor should study of outcomes be divorced from identifying and studying the processes (including social interactions) which led to these outcomes. It is only by understanding how and why an outcome may have come about that we can begin to understand more about shaping contexts and mechanisms in multiple local settings. Without this more and more 'solutions' may be invented without understanding what the problems we are trying to address actually are. Action research methods may be one way to address these issues.

IMPLICATIONS FOR PRACTICE

Although for this work multimorbidity was defined as the 'co-existence of two or more conditions, where one is not necessarily more central' [20]. In practice, it seems the definition of multimorbidity as 'a set of unique constellations of problems, shifting priorities and multi-dimensional decision-making' is more likely to resonate with patients, trainees and GPs [13]. Our synthesis suggests the following items are important for practice development in the interim until further research evidence is available:

1. Concurrency of education and care delivery must not be ignored.
2. GPs should be aware of the non-linear transitions for patients and trainees occurring during social interactions and seek to support active engagement and meaningful roles in healthcare for both by taking responsibility for legitimising participation and providing a safety net which balances challenge with appropriate support.
3. Primary care organisations should seek to create contexts in which patients, trainees and GPs can discuss challenges related to multimorbidity, discuss concepts of success and failure and develop shared goals.

4. Recognition of different sorts of knowledge and practice (including experiential expertise) as valuable for development of new in-practice knowledge is important for patients as well as trainees and GPs
5. Trusting relationships must be cultivated between patients, trainees and GPs to allow for ‘unscripted’ conversations, that lead to needs-based education and care.
   Interventions should be designed to take account of the dynamic systems in which people work as well as targeting education at individuals accounting for breakdowns and work-arounds in interventions (and learning from these).
6. A reduced emphasis on index condition and diagnosis-cure models in long term conditions instead focusing on potential reversibility in negotiation with patients, recognising a spectrum from concordant to discordant in morbidities is needed.

ACKNOWLEDGEMENTS

The authors and collaborative research team thank Geoff Wong, Janet Lefroy,Peter Croft, Peter Coventry, Danielle van der Windt, and Joanne Jordan for their assistance and provision of expertise to allow completion of this work. In addition we are very grateful to Lucy Robertson, of lucyrobertsondesigns for collaborating with us to produce the model artwork.

CITATION LIST OF ARTICLES INCLUDED IN SYNTHESIS

1. Ahmed S, Gogovor A, Kosseim M, Poissant L, Riopelle R, Simmonds M, Krelenbaum M, Montague T : Advancing the chronic care road map: a contemporary overview. Health Quart 2010, 13:72-9.
2. Alderson TS, Bateman H, Alderson TSJ: Doctors telling stories: the place of anecdote in GP registrar training. Med Teach 2002, 24:654–7.
3. Alexander JA, Hearld LR, Mittler JN, Harvey J: Patient-physician role relationships and patient activation among individuals with chronic illness. Health Serv Res 2012, 47:1201-1223.
4. American Geriatrics Society Expert Panel on the Care of Older Adults with Multimorbidity: Guiding principles for the care of older adults with multimorbidity: An approach for clinicians. J Am Geriatr Soc 2012, 60:E1-25.
5. Anonymous: New approach to behavior change geared toward individual practitioners. Dis Manag Advis 2002, 8:93-5, 81.
6. Ashley P, Rhodes N, Sari-Kouzel H, Mukherjee A, Dornan T: 'They've all got to learn'. Medical students' learning from patients in ambulatory (outpatient and general practice) consultations. Med Teach 2009, 31:e24-31.
7. Assal JP: Revisiting the approach to treatment of long-term illness: from the acute to the chronic state. A need for educational and managerial skills for long-term follow-up. Pat Educ Counsel 1999, 37:99-111.
8. Aujoulat I, Marcolongo R, Bonadiman L, Deccache A: Reconsidering patient empowerment in chronic illness: a critique of models of self-efficacy and bodily control. Soc Sci Med 2008, 66:1228-39.
9. Bain DJ: Doctor-patient communication in general practice consultations. Med Educ 1976, 10:125-31.
10. Barclay-Goddard R, King J, Dubouloz CJ, Schwartz CE, Response S: Building on transformative learning and response shift theory to investigate health-related quality of life changes over time in individuals with chronic health conditions and disability. Arch Phys Med Rehabil 2012, 93:214-20.
11. Barnett K, Mercer SW, Norbury M, Watt G, Wyke S, Guthrie B: Epidemiology of multimorbidity and implications for health care, research, and medical education: a cross-sectional study. The Lancet 2012, 380:37-43.
12. Baughan DM, Revicki D, Nieman LZ: Management of problem patients with multiple chronic diseases. J Fam Pract 1983, 17: 233-239.
13. Bayliss A, Edwards E, Steiner F, Main S: Processes of care desired by elderly patients with multimorbidities. Fam Pract 2008, 25:287-293.
14. Bikker AP, Mercer SW, Cotton P. Connecting, Assessing, Responding, Empowering (CARE): A universal approach to person-centred, empathic healthcare encounters. Ed Prim Care 2012, 23:454-457.

15. Bleakley A, Bligh J: Students learning from patients: let's get real in medical education. Adv Health Sci Educ Theory Pract 2008, 13:1573-1677.
16. Boeckxstaens P, De Graaf P: Primary care and care for older persons: position paper of the European Forum for Primary Care. Qual Prim Care 2011, 19:369-389.
17. Boendermaker PM, Conradi MH, Schuling J, Meyboom-de Jong B, Zwierstra RP, Metz JC: Core characteristics of the competent general practice trainer: a Delphi study. Adv Health Sci Educ Theory Pract 2003, 8:111–6.
18. Bower PF, Harkness EF, Macdonald WF, Coventry PF, Bundy CF, Moss-Morris R: Illness representations in patients with multimorbid long-term conditions: Qualitative study. Psychol Health 2012, 27:1121-26.
19. Bower P, Macdonald W, Harkness E, Gask L, Kendrick T, Valderas JM, Dickens C, Blakeman T, Sibbald B: Multimorbidity, service organization and clinical decision making in primary care: A qualitative study. Fam Pract 2011, 28:579-587.
20. Boyd CM, Fontin M: Future of multimorbidity research: how should understanding of multimorbidity inform health system design? Pub Health Rev, 2010 32:451-474.
21. Brown JS, Duguid P: Organizational learning and communities-of practice: toward a unified view of working, learning, and innovation. Org Science 1991, 1:40-57.
22. Burgers JS, Voerman GE, Grol R, Faber MJ, Schneider EC: Quality and Coordination of Care for Patients With Multiple Conditions: Results From an International Survey of Patient Experience. Eval Health Prof 2010, 33:343-364.
23. Cassell EJ: Teaching the fundamentals of primary care: a point of view. Milbank Q 1995, 73):373-405.
24. Chan BC, Perkins D, Wan Q, Zwar N, Daniel C, Crookes P, Harris MF: Finding common ground? Evaluating an intervention to improve teamwork among primary health-care professionals. Int J Qual Health Care 2010, 22:519-24.
25. Cherubini A, Corsonello A, Lattanzio F: Underprescription of beneficial medicines in older people: causes, consequences and prevention. Drugs Aging 2012, 29:463-475.
26. Clark NM, Gong M: Management of chronic disease by practitioners and patients: are we teaching the wrong things? BMJ 2000, 320:572-5.
27. Clark NM, Nothwehr F, Gong M, Evans D, Maiman LA, Hurwitz ME, Roloff D, Mellins RB: Physician-patient partnership in managing chronic illness. Academic Medicine 1995, 70:957-9.
28. Colditz GA: Medical education meeting community needs. Med Educ 1983, 17:291-5.
29. Coles C: Educating the health care team. Pat Educ Counsel 1995, 26:239-44.
30. Cornford C, Carrington B: A qualitative study of the experiences of training in general practice: a community of practice? J Educ Teach 2006, 32: 269–82.
31. Corser WD: Increasing Primary Care Comorbidity: A Conceptual Research and Practice Framework. Res Theory Nurs Pract 2011, 25:238-251.
32. Cote L, Leclere H. How clinical teachers perceive the doctor–patient relationship and themselves as role models. Acad Med 2000, 75 :1117–24.
33. Cowie L, Morgan M, White P, Gulliford M: Experience of continuity of care of patients with multiple long-term conditions in England. J Health Serv Res Policy 2009, 14:82-87.
34. Darer JD, Hwang W, Pham HH, Bass EB, Anderson G: More training needed in chronic care: a survey of US physicians. Acad Med 2004, 79:541-8.
35. Dawes M. Co-morbidity: we need a guideline for each patient not a guideline for each disease. Fam Pract 2010, 27:1-2.

36. Dent MM, Mathis MW, Outland M, Thomas M, Industrious D: Chronic disease management: teaching medical students to incorporate community. Fam Med 2010, 42:736-40.
37. de Jong J, Visser MR, Mohrs J, Wieringa-de Waard M: Opening the black box: the patient mix of GP trainees. Brit J Gen Pract 2011, 61:e650-7.
38. Department of Health: Improving quality of life for people with long term conditions. Department of Heath, 2013. London. Available at: [https://www.gov.uk/government/policies/improving-quality-of-life-for-people-with-long-term-conditions] (last accessed 31.07.14)
39. Dornan T, Scherpbier A, Boshuizen H: Supporting medical students' workplace learning: experience-based learning (ExBL). Clin Teach 2009, 6: 167-71.
40. Dornan T, Boshuizen H, King N, Scherpbier A: Experience-based learning: a model linking the processes and outcomes of medical students' workplace learning. Med Educ 2007, 41: 84-91.
41. Dubouloz CJ, King J, Paterson B, Ashe B, Chevrier J, Moldoveanu M: A model of the process of transformation in primary care for people living with chronic illnesses. Chron Illness 2010a, 6:282-93.
42. Dubouloz, CJ, King J, Ashe B, Paterson B, Chevrier J, Moldoveanu M: The process of transformation in rehabilitation: what does it look like? Int J Ther Rehabil 2010b, 17:604-15.
43. Durso S: Using clinical guidelines designed for older adults with diabetes mellitus and complex health status. JAMA 2006, 295:1935–1940.
44. Ekdahl AW, Hellstrom I, Andersson L, Friedrichsen M: Too complex and time-consuming to fit in! Physicians' experiences of elderly patients and their participation in medical decision making: a grounded theory study. BMJ Open 2012, 2:e001063.
45. Ferenchick G, Simpson D, Blackman J, DaRosa D, Dunnington G: Strategies for efficient and effective teaching in the ambulatory care setting. Acad Med 1997, 72:277–80.
46. Fernald DH, Staudenmaier CJ, Main DS, O'Brien-Gonzales A, Barley GE: Student perspectives on primary care preceptorships: enhancing the medical student preceptorship learning environment. Teach Learn Medicine 2001, 13:13-20.
47. Fortin M, Hudon C, Bayliss EA, Soubhi H, Lapointe L: Caring for body and soul: The importance of recognizing and managing psychological distress in persons with multimorbidity. Int J Psych Med 2007, 37:1-9.
48. Fortin M, Soubhi H, Hudon C, Bayliss EA, van den Akker M: Multimorbidity's many challenges. BMJ 2007, 334:1016-1017.
49. Fortin M, Bravo G, Hudon C, Lapointe L, Dubois MF, Almirall J: Psychological distress and multimorbidity in primary care. Ann Fam Med 2006, 4:417-422.
50. Fried T, McGraw S, Agostini J, Tinetti M: Views of older persons with multiple morbidities on competing outcomes and clinical decision making. J Am Geriatr Soc 2008, 56: 1839-44.
51. Frueh BC, Larme AC, Noel PH, Pugh JA: Collaborative care needs and preferences of primary care patients with multimorbidity. Health Expect 2005, 8:54-63.
52. Gabbay J, le May A: Evidence based guidelines or collectively constructed "mindlines?" Ethnographic study of knowledge management in primary care. BMJ 2004, 329:1013.
53. Gaver A, Borkan JM, Weingarten MA: Illness in context and families as teachers: a year-long project for medical students. Acad Med 2005, 80:448-51.
54. Geyman JP, Bliss E. What does family practice need to do next? A cross-generational view. Fam Med 2001, 33:259-67.

55. Glasgow RE, Davis CL, Funnell MM, Beck A: Implementing practical interventions to support chronic illness self-management. Joint Commission Journal on Quality & Safety 2003, 29:563-74.
56. Glasgow NJ, Wells R, Butler J, Gear A: The effectiveness of competency-based education in equipping primary health care workers to manage chronic disease in Australian general practice settings. Med J Aust 2008, 188:S92-6.
57. Guthrie B, Payne K, Alderson P, McMurdo MET, Mercer SW: Adapting clinical guidelines to take account of multimorbidity. BMJ 2012, 345:e6341.
58. Haas WH, Crandall LA, Bain DJ: Characteristics of family practitioners with large geriatric practices. J Am Geriatr Soc 1980, 28:289-94.
59. Haggerty JL: Ordering the chaos for patients with multimorbidity. BMJ 2012, 7876:e5915.
60. Haidet P, Kroll TL, Sharf BF: The complexity of patient participation: lessons learned from patients' illness narratives. Pat Educ & Counsel 2006, 62:323-9.
61. Heath I, Rubinstein A, Stange KC, van Driel ML: Quality in primary health care: a multidimensional approach to complexity. Br Med J 2009, 338:b1242.
62. Heath I. In praise of young doctors. BMJ 2012, 345:e4549-e4550.
63. Henschen BL, Garcia P, Jacobson B, Ryan ER, Woods DM, Wayne DB, Evans DB: The patient centred medical home as curricular model: perceived impact of the "Education-Centred Medical Home". J Gen Int Med 2013, 28:1105-9.
64. Higashi T, Wenger NS, Adams JL, Fung C, Roland M, McGlynn EA, Reeves D, Asch SM, Kerr EA, Shekelle PG : Relationship between number of medical conditions and quality of care. N Engl J Med 2007, 356:2496-504.
65. Hobbs J, Speers S, Herbert J, Nixon G, Poteet L, Hatch P: Clinical resources to teach components of a new Family Medicine Clerkship Curriculum. Fam Med 2011, 43:566-73.
66. Hughes LD, McMurdo, ME, Guthrie, B: Guidelines for people not for diseases: the challenges of applying UK clinical guidelines to people with multimorbidity. Age Aging 2013, 42:62-69.
67. Jackson CL, Askew DA, Nicholson C, Brooks PM: The primary care amplification model: taking the best of primary care forward. BMC Health Serv Res 2008, 8:268.
68. Johnson JK, Woods DM, Stevens DP, Bowen JL, Provost LP, Sixta CS, Wagner EH: Joy and challenges in improving chronic illness care: capturing daily experiences of academic primary care teams. J Gen Int Med 2010, 25:S581-5.
69. Jones DS: Needed: A coherent architecture for 21st-century clinical practice and medical education. Alt Ther Health Med 2010, 16:64-7.
70. Jordan ME, Lanham HJ, Crabtree BF, Nutting PA, Miller WL, Stange , Kurt C, McDaniel , Reuben R Jr: The role of conversation in health care interventions: Enabling sensemaking and learning. Implement Sci 4:1-13.
71. Kadam U: Redesigning the general practice consultation to improve care for patients with multimorbidity. BMJ 2012, 345:e6202.
72. Kamerow D: How can we treat multiple chronic conditions? BMJ 2012, 29:e1487.
73. Kernick D: A theoretical framework for multimorbidity: from complicated to chaotic. Br J Gen Pract 2012,:e659-e661.
74. King D Benbow SJ, Elizabeth J, Lye M: Attitudes of elderly patients to medical students. Med Educ 1992, 26:360-3.
75. Klinkman M, van Weel C: Prospects for person-centred diagnosis in general medicine. J Eval Clin Pract 2011, 17:365-370.

76. Lacroix A, Jacquemet S, Assal JP, Benroubi M: The patients' voice: testimonies from patients suffering from chronic disease. Pat Educ Counsel 1995, 26:293-9
77. Légaré F, Ratté S, Stacey D, Kryworuchko J, Gravel K, Graham ID, Turcotte S: Interventions for improving the adoption of shared decision making by healthcare professionals. Cochrane Database Syst Rev 2010, 12: CD006732.
78. Legare F, Stacey D, Graham ID, Elwyn G, Pluye P, Gagnon M, Frosch D, Harrison MB, Kryworuchko J, Pouliot S, Desroches S. Advancing theories, models and measurement for an interprofessional approach to shared decision making in primary care: a study protocol. BMC Health Serv Res 2008, 8:2.
79. Legare F, Turcotte S, Stacey D, Ratte S, Kryworuchko J, Graham ID. Patients' Perceptions of Sharing in Decisions A Systematic Review of Interventions to Enhance Shared Decision Making in Routine Clinical Practice. Patient 2012, 5:1-19.
80. Leykum LK, Palmer R, Lanham H, Jordan M, McDaniel RR, Noel PH, Parchman M: Reciprocal learning and chronic care model implementation in primary care: results from a new scale of learning in primary care. BMC Health Serv Res 2011, 11:44.
81. Loffler C, Kaduszkiewicz H, Stolzenbach C, Streich W, Fuchs A, van den Bussche H, Stolper F, Altiner A: Coping with multimorbidity in old age - a qualitative study. BMC Fam Pract 2012, 13:45.
82. Logan R: The teaching of community medicine in the undergraduate curriculum. Br J Med Educ 1969, 3:185-91.
83. Lugtenberg M, Zegers-van JM,JM, Westert GP, Burgers JS: Why don't physicians adhere to guideline recommendations in practice? An analysis of barriers among Dutch general practitioners. Implement Sci 2009, 4:54.
84. Luijks HD, Loeffen MJW, Lagro-Janssen AL, van Weel C, Lucassen PL, Schermer TR: GP's considerations in multimorbidity management: a qualitative study. Brit J Gen Pract 2012, 62:e504-e510
85. Mangin D, Heath I, Jamoulle M: Beyond diagnosis: rising to the multimorbidity challenge. BMJ 2012, 344:e3526-e3528.
86. Mann K, Holmes DB, Hayes VM, Burge FI, Viscount PW: Community family medicine teachers' perceptions of their teaching role. Med Educ 2001, 35:278-85.
87. Marsteller JA, Hsu YJ, Reider L, Frey K, Wolff J, Boyd C, Leff B, Karm L, Scharfstein D, Boult C: Physician satisfaction with chronic care processes: a cluster-randomized trial of guided care. Ann Fam Med 2010, 8:308-15.
88. Martin C, Rohan BG: Chronic illness care as a balancing act. A qualitative study. Aust Fam Physic 2002, 31:55-9.
89. Morris R, Sanders C, Kennedy A: Shifting priorities in multimorbidity: a longitudinal qualitative study of patients' prioritization of multiple conditions. Chron Illness 2011,7:147.
90. Matthews C: Role modelling: how does it influence teaching in family medicine? Med Educ 2000, 34:443–8.
91. Mayor V: Long-term conditions. 3: Being an expert patient. Br J Comm Nurs 2006, 11:59-63.
92. Mercer SW, Jani BD, Maxwell M, Wong SY, Watt GC: Patient enablement requires physician empathy: a cross-sectional study of general practice consultations in areas of high and low socioeconomic deprivation in Scotland. BMC Fam Pract 2012, 13:6.
93. Mercer. Managing patients with mental and physical multimorbidity. BMJ 2012, 345:e5559.

94. Mishra SI, Gioia D, Childress S, Barnet B, Webster RL: Adherence to medication regimens among low-income patients with multiple comorbid chronic conditions. Health Soc Work 2011, 36:249-58.
95. Moth G: Chronic care management in Danish general practice - a cross-sectional study of workload and multimorbidity. BMC Fam Pract 2012, 13:52.
96. Muir F: Placing the patient at the core of teaching. Med Teach 2007, 29:258-60.
97. Munro N, Hornung R, McAleer S: What are the key attributes of a good general practice trainer: a Delphi study. Educ Gen Pract 1998, 9:263–70.
98. Nieman LZ, Cheng L: Chronic illness needs educated doctors: An innovative primary care training program for chronic illness education. Med Teach 2011; 33:e340-8.
99. NHS Improvement: Effective pathways for long term conditions. Leicester : NHS Improvement, 2012 Web publication. http://www.improvement.nhs.uk/documents/LTC_Brochure.pdf
100. Noel PH, Parchman ML, Williams JW, Cornell JE, Shuko L, Zeber JE, Kazis LE, Lee AFS, Pugh JA:The challenges of multimorbidity from the patient perspective. Journal of General Internal Medicine 2007, 22: 419-24.
101. O'Brien R, Wyke S, Guthrie B, Watt G, Mercer S: An 'endless struggle': A qualitative study of general practitioners' and practice nurses' experiences of managing multimorbidity in socio-economically deprived areas of Scotland. Chronic Illness, 2011 7:45-59.
102. O'Flynn N, Spencer J, Jones R: Does teaching during a general practice consultation affect patient care? Br J Gen Pract, 1999. 49:7-9
103. O'Flynn N. Improving the experience of care for people using NHS services: summary of NICE guidance. BMJ 2012, 344:d6422.
104. O'Sullivan M, Martin J, Murray E: Students' perceptions of the relative advantages and disadvantages of community-based and hospital-based teaching: A qualitative study. Medical Education. 2000, 34:648–655.
105. Parboosingh IJ, Reed VA, Palmer JC, Bernstein HH: Enhancing Practice Improvement by Facilitating Practitioner Interactivity: New Roles for Providers of Continuing Medical Education. J Contin Educ Health Prof 2011, 31:122-127.
106. Pearson DJ, Lucas BJ: Engagement and opportunity in clinical learning: findings from a case study in primary care. Med Teach 2011, 33:670–7.
107. Peile EB, Easton GP, Johnson N: The year in a training practice: what has lasting value? Grounded theoretical categories and dimensions from a pilot study. Med Teach 2001, 23:205–11.
108. Phillips J. Patients can educate doctors about long term disease. BMJ 1999, 319:785.
109. Pichlhofer O, Tonies H, Spiegel W, Wilhelm-Mitteracker A, Maier M: Patient and preceptor attitudes towards teaching medical students in general practice. BMC Medical Education 2013, 13:33.
110. Rakel D: Creating expertise in health and healing. Journal of the American Board of Family Medicine. J Am Board Fam Med 2007, 20:611.
111. Roland M, Paddison, C: Better management of patients with multimorbidity. BMJ 2013, 346:f2510.
112. Russell G, Thille P, Hogg W, Lemelin J: Beyond fighting fires and chasing tails? Chronic illness care plans in Ontario, Canada. Ann Fam Med 2008, 6:146-153.
113. Salisbury C. Multimorbidity: redesigning health care for people who use it. Lancet 2012, 380:7-9.

114. Sagasser MH, Kramer AWM, van der Vleuten CPM: How do postgraduate GP trainees regulate their learning and what helps and hinders them? A qualitative study. BMC Med Educ 2012, 12: 67.
115. Schers H, Webster S, van den Hoogen H, Avery A, Grol R, van den Bosch W: Continuity of care in general practice: a survey of patients' views. Br J Gen Pract 2002, 52: 459-62.
116. Schneider A, Korner T, Mehring M, Wensing M, Elwyn G, Szecsenyi J: Impact of age, health locus of control and psychological co-morbidity on patients' preferences for shared decision making in general practice. Pat Educ Counsel 2006, 61:292-8.
117. Schuez B, Wurm S, Warner LM, Ziegelmann JP: Self-efficacy and multiple illness representations in older adults: A multilevel approach. Psychol Health 2012, 27:13-29.
118. Schuling J, Gebben H, Veehof LJG, Haaijer-Ruskamp FM: Deprescribing medication in very elderly patients with multimorbidity: the view of Dutch GPs. A qualitative study. BMC Fam Pract 2012, 13:56-61.
119. Scott J, Tallia A, Crosson JC, Orazano AJ, Stroebel C, DiCicco-Bloom B, O'Malley D, Crabtree B: Social network analysis as an analytic tool for interaction patterns in primary care practices. Ann Fam Med. 2005, 3:443-448.
120. Sherbourne CD, Sturm R, Wells KB: What outcomes matter to patients? J Gen Intern Med 1999, 14:357-66.
121. Smith CS, Irby DM: The roles of experience and reflection in ambulatory care education. Acad Med 1997, 72:32–5.
122. Smith CS, Morris M, Francovich C, Hill W, Gieselman J: A qualitative study of resident learning in the ambulatory clinic. The importance of exposure to 'breakdown' in settings that support effective response. Adv Health Sci Educ 2004, 9:93–105.
123. Smith SM, O'Kelly S, O'Dowd T: GPs' and pharmacists' experiences of managing multimorbidity a 'Pandora's box'. Br J Gen Pract 2010, 60:501-503.
124. Smith SM, Soubhi H, Fortin M, Hudon C, O'Dowd T: Interventions for improving outcomes in patients with multimorbidity in primary care and community settings. Cochrane Database Syst Rev 2012, CD006560.
125. Smith SM: Managing patients with Multimorbidity: systematic review of interventions in primary care and community settings. BMJ 2012, 345:e5205.
126. Soubbi H, Bayliss EA, Fontin M, Hudon C, van der Akker M, Thivierge R, Posel N, Fleiszer D: Learning and caring in communities of practice: using relationships and collective learning to improve primary care for patients with multimorbidity. Annals Fam Med 2010, 8:170-177.
127. Soubhi H: Toward an ecosystemic approach to chronic care design and practice in primary care. Annals Fam Med 2007, 5:263-9.
128. Stewart JHP: Learning from the learners: what do trainees want from general practice vocational training? Asia Pac Fam Med 2002, 1:28–32.
129. Svenberg K: A memorable consultation: Writing reflective accounts articulates students' learning in general practice. Scand J of Prim Health Care 2007, 25:75–79.
130. Swanick T, Plint S: From supernumerary to supervised professional development: work-based learning for specialist training for general practice. Educ Prim Care 2006, 17 :97–103.
131. Tinetti ME, Fried TR, Boyd CM: Designing health care for the most common chronic condition – Multimorbidity. J Am Med Assoc 2012, 307:2493-2494.
132. Townsend A. Applying Bourdieu's theory to accounts of living with multimorbidity. Chron Illness 2012, 8:89-101.

133. Treasure W: First do no harm:education deals with the application of general principles in uncertain situations. Br J Gen Pract 2013, 63:151.
134. van der Zwet J, Zwietering PJ, Teunissen P, van der Vleuten CPM, Scherpbier, AJ J A: Workplace learning from a socio-cultural perspective: creating developmental space during the general practice clerkship. Adv Health Sci Educ 2011, 16:359-73.
135. van Dijk-de Vries A, Moser A, Mertens V, van der Linden J, van der Weijden T, Th. M van Eijk, J: The ideal of biopsychosocial chronic care: how o make it real? A qualitative study among Dutch stakeholders. BMC Fam Pract 2012, 13:14.
136. van Walraven C, Oake N, Jennings A, Forster AJ: The association between continuity of care and outcomes: a systematic and critical review. J Eval Clin Pract 2010, 16:947-56.
137. Wagner EH, Austin BT, Davis C, Hindmarsh M, Schaefer J, Bonomi A: Improving chronic illness care: translating evidence into action. Health Affairs 2001, 20:64-78.
138. Wearne S, Dornan T, Teunissen PW, Skinner T: General practitioners as supervisors in postgraduate clinical education: an integrative review. Med Educ 2012, 46:1161-73.
139. Wehling, M: Guideline-Driven Polypharmacy in Elderly, Multimorbid Patients is Basically Flawed: there are almost no Guidelines for these Patients. J Am Geriatr Soc 2011, 59:376-376.
140. Yardley S, Teunissen PW, Dornan T: Experiential learning: AMEE Guide No. 63. Med Teach. 2012; 34:e102-15
141. Zweig S, Williamson HA, Jr: Adverse effects of faculty practice on diagnostic content of residents' outpatient experience. J Fam Pract 1987, 25:491-6.

APPENDICES

4. ADDITIONAL SENSITIVITY SEARCH IN MEDLINE DATABASE
5. SUMMARY OF STUDIES CONTAINING MODELS / SUMMARY OF OTHER EMPIRICAL STUDIES
6. MARKERS OF SUCCESS AND FAILURE INDENTIFIED IN THE SYNTHESIS

APPENDIX 1: ADDITIONAL SENSITIVITY SEARCH IN MEDLINE DATABASE

**1 AND 2**	**1025 (974 in humans – imported into refworks for screening AND REDUCED TO 10 ON TITLE SCREENING)**

1. Multimorbidity

**No**	**Search term**
**1**	exp COMORBIDITY/
**2**	comorbid*.ti,ab
**3**	co-morbid*.ti,ab
**4**	multimorbid*.ti,ab
**5**	multi-morbid*.ti,ab
**6**	exp CHRONIC DISEASE
**7**	(chronic adj2 disease*) .ti,ab
**8**	exp DISEASE PROGRESSION
**9**	(disease adj2progress*). Ti, ab
**10**	1 OR 2 OR 3 OR 4 OR 5 OR 6 OR 7 OR 8 OR 9

2. Additional search terms

**No**	**Search term**	
**1**	Exp *AMBULATORY CARE/ed (ed=education)	7
**2**	(role adj2 model)	1366
**3**	Supervis* (to cover supervisor and supervisee)	35989
**4**	1 OR 2 OR 3	37324

APPENDIX 2: SUMMARY OF STUDIES CONTAINING MODELS / OTHER EMPIRICAL STUDIES*

*see citation list following references in main paper for full citations

Studies containing models

CITATION 4: AGS 2012	
Country of origin	USA
Study design	Literature review using 4 search strategies: patient preferences, interpreting evidence/prognosis/clinical feasibility, optimising therapies, and care plans.
Context	Work conducted by an expert panel from the American Geriatrics Society in order to achieve consensus on best practice in clinical approaches to the care of older adults with multimorbidity.<br><br>Drivers to develop 'guiding principles': Multimorbidity associated with multiple adverse consequences; Non-disease specific healthcare delivery strategies required; Best approaches to decision making and clinical management of older adults with multimorbidity unclear, and; Clinicians need a management approach that will consider the multiple problems particular to each individual with multimorbidity.
Intervention	Theory: disease specific approaches to clinical guidelines / healthcare and research systems fail patients (particularly the elderly) with multimorbidity as they are excluded from studies of best practice.<br><br>Identified 5 domains of importance: patient preferences; interpreting evidence; prognosis; clinical feasibility; optimising therapies/care plans.
Mechanisms triggered	Guiding principles not tested in practice.
Reported outcomes	N/A
Authors interpretation	Recommends a model approach which focuses first on each patient's primary concern; this should trigger either a complete review of their care plan or addressing a specific aspect in negotiation with the patient. Prognosis should be considered as an important contextual factor along with interactions within and among treatments and conditions, weighing benefit and harm. Regular reassessment should also be planned to monitor implementation and adjust the plan as required.
Strengths and limitations	This is a well-constructed piece of work which combines literature review with expert consensus. As such it demonstrates current thinking amongst clinicians about best practice. The model of care is, however, currently untested in research studies.
Our interpretation	There is need for work to understand how to develop individualised patient care for people with multimorbidity. Success and failure are conceptualised in this paper as determined by patient preference with shared patient-professional responsibility for risk.
Use made of citation in synthesis (contribution to theory building)	Model approach used to develop new theory of important elements of doctor-patient-learner interactions in the context of multimorbidity.

CITATION 6: Ashley et al 2008	
Country of origin	UK
Study design	Semi structured exit interviews with patients (n=25) and medical students (n=8) following ambulatory teaching consultations (n=25). Focus groups (n=2) with students and students/doctors to discuss the emerging analysis. Grounded theory analysis.
Context	These were year 3 undergraduate students who had selected a study module in ambulatory care (outpatient clinic or general practice). Patients were unselected from these settings.
Intervention	Students set own learning outcomes and had an induction (practice consultation) prior to attending clinics. Weekly meetings with supervisor and senior students to supplement 'on the job' learning. On average students attended 3 outpatient and 1 general practice clinic sessions.
Mechanisms triggered	Major themes identified: patient/student reactions to teaching consultations; dynamics between patients and students; factors affecting dynamics. Effective consultations were defined as those in which doctors promoted participation and direct interaction between patients and students, as well as providing debriefing for students.
Reported outcomes	Suggests that there is a need to re-orientate relationships between students, doctors and patients to enhance collaboration between students and patients, with doctors positioned as an expert resource. Without this there is a risk both students and patients will default to passive roles which decrease the value of interaction for both groups.
Authors interpretation	Identified that learning required a supportive environment with clear expectations of students and allowing them to have a purposeful (authentic) role in patient care.<br><br>Patients were keen to participate and had insight into the learning that could be gained from their experiences of illness; some reported benefit from students asking questions they didn't feel able to ask the doctors themselves.<br><br>Both students and patients identified positive emotional, cognitive and practical benefits from teaching consultations.

Strengths and limitations	Although this paper was not specifically about multimorbidity (it was about specific chronic illness management) it is a well conducted study that sought to analyse and interpret underlying social processes of student-patient-doctor interactions which are very relevant to our research question, particularly with respect to highlighting the power accorded by students and patients to the role of the doctor.
Our interpretation	Doctors needed to be able to take responsibility for supervision of student-patient interactions through risk management rather than avoidance.
Use made of citation in synthesis (contribution to theory building)	Findings used to refine new theory of important elements of doctor-patient-learner interactions in the context of multimorbidity.

**CITATION 10: Barclay-Goddard et al 2012**	
Country of origin	Canada
Study design	Think-tank working group (policy developers) comparing transformative learning theory and response shift theory.
Context	People with disability or chronic illness.
Intervention	Theoretical paper.<br>(NB. Models of Transformation also commonly used in experiential learning theories)
Mechanisms triggered	Considers two models of change: Transformative learning theory; and Response shift.<br><br>Drawing on these the authors produce a model for the process of transformation in chronic illness and disability. The process is broadly divided into three categorisations: trigger(s), process of changing and outcomes.<br><br>Triggers: lived experiences combined with readiness for change<br>Process of changing: critical reflection, identifying issues, restructuring meanings / constructing new meanings, addressing barriers to change, support from facilitators to change<br>Outcomes: new rules, ways or guidelines, new behaviours, new feelings, new beliefs, new perspectives, new identity
Reported outcomes	N/A
Authors interpretation	Argues that chronic health conditions can be considered in similar ways to disability: both are triggered by health crises that demands in-depth personal change to regaining meaning in life. Suggests that transformative learning and response shift provide theoretical models for professionals and patients to work together to redefine success in the absence of cure.
Strengths and limitations	Based around a clinical scenario of paraplegia that illustrates how perceptions of health change unpredictably following altered circumstances. Limitations of both models are noted to be the requisite need for someone (learner or patient) to be in a state of 'readiness for change'. Model not tested in practice
Our interpretation	Transformative learning is a model appearing across our literature dataset, with respect to both education and healthcare. It involves a complex process of personal change which is triggered by experiences. Critique of the model often focuses on tendencies to assume 'good' transformation can always occur but this is not what proponents of the model necessarily mean by 'transformation'. For example, Mezirow et al (ref) argue that we are all changed by our life experiences, for better or worse, and that we all learn in the sense of creating meaning and constructing personal knowledge about the world as a result of this.<br>Response shift theory is not dissimilar in approach: this describes a change in internal standards, values or definitions of constructs over time (and with experience).
Use made of citation in synthesis (contribution to theory building)	Theoretical basis for new model developed from synthesis.

**CITATION 14: Bikker et al 2012**	
Country of origin	UK
Study design	Report on the theoretical development of a model (CARE Approach).
Context	Model of consultation targeted at general practice / community consultations.
Intervention	Development of a model of practice 'The CARE approach' for general practice consultations which emphasises human / relational aspects of healthcare.<br><br>Described as a 'multidisciplinary learning tool that focuses on human aspects of care' with 4 components: connecting, assessing, responding and empowering. Measure of application also developed (patient ratings on components).
Mechanisms triggered	Implementation in progress (pilot projects but results not yet published) about using the model as part of an educational package.
Reported outcomes	Not yet tested.
Authors interpretation	N/A
Strengths and limitations	Theoretical model of best practice in patient centred consultations which needs further testing in 'real world' practices.
Our interpretation	Implicitly defines concepts of success and failure as a result of negotiation between individual patients and professionals.
Use made of citation in	Provided theoretical basis for human / relational focus in new model of interactions: components of CARE

synthesis (contribution to theory building)	model support the proposed model of interactions as ways in which all participants can learn new ways of working collaboratively together.

**CITATION 15: Bleakley et al 2008**	
Country of origin	UK
Study design	Theoretical development of concept of patient-centred medical education
Context	Medical students are the main focus of the paper although the underlying argument is that learning should be more patient-centred.
Intervention	Development of a theoretical model of patient-centred medical education.
Mechanisms triggered	Theoretical model, not tested.
Reported outcomes	N/A
Authors interpretation	Argues for greater patient-learner interaction with the doctor (trainer) as an expert director rather than on-hand overseer i.e. the doctor should be positioned as an educational resource rather than the patient. Suggests that medical students need to be legitimate participants in authentic patient care to produce optimal care as well as optimal learning in the long term. Learning should be for patients.
Strengths and limitations	A well-reasoned argument that resonates with other papers in the synthesis.
Our interpretation	Successful medical education is conceptualised as what produces optimal patient care.
Use made of citation in synthesis (contribution to theory building)	Used to refine ideas about interactions between 2 or more of patients, learners and doctors.

**CITATION 18: Bower et al 2012**	
Country of origin	UK
Study design	Qualitative study of patients' representations of multi-morbid long term conditions using semi-structured interviews (n=28).
Context	Patients with multimorbidity.
Intervention	Theories of self-regulation such as the 'common sense model' of paying attention to both cognitive and emotional aspects of illness.
Mechanisms triggered	Explored patient representations of their individual illnesses and of multimorbidity: the latter was found to impact on identity, perceived cause, coherence and consequences of illnesses. The burden of medication and perceived priorities, synergies or antagonisms among conditions / management were common themes. Living with combinations of conditions was perceived to have greater impact than the sum of the parts.
Reported outcomes	Very few patients told a story of complex interacting and interweaved conditions, most rather described variable priorities and foci according to current functional impact.
Authors interpretation	Presents patient perspectives on multimorbidity highlighting how these might impact on care models which seek to increase shared-decision making and self-management.
Strengths and limitations	Well conducted study which provides useful understanding of the complexities of multimorbidity.
Our interpretation	No clear outcomes identified.
Use made of citation in synthesis (contribution to theory building)	Used to enrich new interpretation of transformation in the face of multimorbidity from a patient perspective.

**CITATION 20: Boyd et al 2010**	
Country of origin	USA/Canada
Study design	Theoretical review of implications of multimorbidity for health system design and research needs.
Context	Sets out to review the implications of multimorbidity for the design of healthcare systems and to understand research needs in this area.
Intervention	Provides what has been accepted as a standard definition of multimorbidity 'the co-existence of two or more chronic conditions, where one is not necessarily more central than the others'<br><br>Draws on the 'Chronic Care Model' (Wagner et al) – with an emphasis on patient-centred approach. This model considers community resources and policies, self-management support, delivery system design, decision support and clinical information systems as essential parts of health organisation that are required to produce productive interactions between an informed and activated patient (plus their family) with prepared and proactive healthcare teams.
Mechanisms triggered	Within this paper the Chronic Care Model is not tested although other papers within the synthesis also use this model, demonstrating it has resonance with practitioners and researchers.

Reported outcomes	Model not tested.
Authors interpretation	See below.
Strengths and limitations	This is a useful analysis of the different component parts of healthcare systems which need to be considered although it is a theoretical paper.
Our interpretation	Failures of healthcare systems are identified as including: lack of matching patients' needs to best therapeutic practices; focus on single dimension of care; fragmentation of care; avoidable inpatient admissions or preventable complications; receiving interventions for which there is little evidence of benefit and high risk of adverse health events.<br><br>Success is conceptualised as: decisions made within the context of a person; appropriate prioritisation of goals and management; holistic approaches.
Use made of citation in synthesis (contribution to theory building)	Concepts used to refine new model of interactions between patients, learners and doctors, considering wider context.

**CITATION 24: Chan et al 2010**	
Country of origin	Australia
Study design	Intervention in general practices (workshop and structured facilitation with materials /telephone support) with qualitative study of impact.
Context	Intervention targeted at improving teamwork between primary care professionals – general practice staff and allied health providers.<br><br>Team-working had been incentivised by the Australian government: GPs received funding to refer patients to subsidised services with allied health professionals. GPs act as co-ordinators who negotiate with patients and professionals about treatment goals and appropriate care.
Intervention	Evening workshop for GPs (n=35) and a range of healthcare professionals (n=39) providing chronic disease care. This was followed by structured facilitation activities using written materials and telephone support in 26 practices. Goal was to improve MDT working. Drew on theories of collaboration: who linked with who. Data was generated through facilitators' observations, GP reports and a survey of allied health professionals. This was analysed qualitatively.
Mechanisms triggered	Patients were more likely to be playing an active role in three way interactions with GPs and allied health professionals.
Reported outcomes	Improved attitudes to MDT working and increased patient roles in healthcare.
Authors interpretation	Creating personal relationship and connections important.
Strengths and limitations	This paper is about learning for patients and qualified professional including doctors – learning to work as a team together. Although measureable outcomes are discussed the actual work only reports changes in attitude.
Our interpretation	Patients, GPs and allied health professionals knowing each other personally increased interactions and team-working: collaboration seemed to depend on personal relationships.<br><br>Success - Reduced hospitalisations, improved care co-ordination, better health outcomes, GPs sharing tasks that were one their sole province that lead to more efficient care.<br><br>Failure – Lack of team work, face to face interaction and understanding of roles and capabilities.
Use made of citation in synthesis (contribution to theory building)	Informed focus on three-way interactivity.

**CITATION 31: Corser et al 2011**	
Country of origin	USA
Study design	Theoretical development of a conceptual framework for research and practice in comorbidity
Context	Comorbid (defined as multiple chronic conditions leading to medical complexity) adults interacting with primary care clinicians.
Intervention	Theoretical article which attempts to develop a conceptual framework for research and practice. Suggests that 'contrasting perspectives' and 'perceptual differences' between patients, clinicians and systems need to be reconciled to improve care outcomes. Suggests conditions contributing to comorbidity should be viewed on a spectrum from concordant to discordant.
Mechanisms triggered	Suggests further work is needed in primary care to develop frameworks for care delivery that account for the experiences of adults with comorbidity. Critiques models reliant on an index condition as the impact of interacting chronic conditions can be synergistic, vary between individuals (regarding symptoms, impact and patient responses).

Reported outcomes	Patient-centred care is the aim of the theoretical model proposed. Suggests development of individualised care plans / profiles for patients and clinicians to work together from.
Authors interpretation	Suggests that a framework explicitly recognising contrasting perspectives could be used to design interventions compatible with patient needs, clinicians practice approaches and circumstances and to address patient-centeredness in interactions. Emphasises the need to prospective study interventions to engage patients and professionals in order to understand underlying beliefs that shape interactions, as well as referring to the Chronic Care Model (Wagner et al 2001) regarding system design, although these authors argue that standardisation is an inappropriate aim due to the multiple variables present in any specific patient-professional interaction.
Strengths and limitations	High quality theoretical paper.
Our interpretation	Highlights the debates about how best to conceptualise comorbidity, approach contrasting clinician and patient perceptions, and the need to better understand interactions between people in this context.<br><br>Suggests that patients are usually more focused on types and severity of symptoms and/or conditions, functional demands, informational / management needs. In contrast suggests that primary care clinicians are more focused on identification of an index condition, complications risks, healthcare system constraints, medications and lab values and provider preferences. This leads, as argued by the authors, to push clinicians towards episodic medical management while patients are seeking help with routine self-management. Tensions between these perspectives impact on patterns of healthcare use and outcomes.
Use made of citation in synthesis (contribution to theory building)	Elements of model used to develop new theory of interactions.

**CITATION 40: Dornan et al. 2007**	
Country of origin	UK
Study design	Model synthesised from grounded theory analysis of group discussions before and after experimental changes in workplace-based learning for medical students (n=36).
Context	Medical students (UK undergraduates), learning in workplaces.
Intervention	Model developed from grounded theory analysis of focus groups: key elements include recognition of the role human interactions and curriculum factors play, the need for challenge to be coupled with support for supported participation that comes with graded responsibilities as the learner progresses from observer to actor. Goals are a positive state of mind and practical competence to be a doctor.
Mechanisms triggered	Identifies that learners need two qualities: practical competence and a state of mind that includes confidence, motivation and sense of professional identity. This must be combined with participation in practice settings to generate workplace-based clinical learning. Supervisors need to balance being supportive with being challenging – offering learners active roles and responsibilities while providing legitimacy and scaffolding for learning.
Reported outcomes	Model highlights the possibility of 'virtuous' or 'vicious' learning cycles: developing competence and a positive state of mind increases capacity for participation in the workplace but this is strongly dependent on interactions with other people. Negative attitudes to learners can produce destructive effects.
Authors interpretation	Suggests that a misapplication of 'safety agendas' can be detrimental to learning in the short-term and patient care in the long-term: through the employment of risk avoidance, rather than risk management, strategies. These authors suggest that more attention should be paid to contextual factors, including human interactions and curriculum design in order to improve the process of supported participation and learning outcomes associated with a positive state of mind to being able to perform and enact the learning necessary if the goal of producing doctors is to be realised.
Strengths and limitations	Good use of empirical data to inform a model of workplace-based learning.
Our interpretation	Highlights that student's motivation to succeed in learning is often defined as pleasing their supervisors and 'fitting in' to the workplace. By implication it is dependent on these supervisors to both give patients a voice in learning and to role model constructive approaches to thinking about success and failure in the absence of cure.
Use made of citation in synthesis (contribution to theory building)	Model used to develop new theory of learner – patient, learner-workplace, learner-supervisor interactions and also to conceptualise interactions between relatively short-term learning goals and impact on patient care in the longer term (i.e. developing a workforce able to deliver appropriate care in the future).

**CITATION 41: Dubouloz et al 2010a**	
Country of origin	Canada
Study design	Literature review of qualitative research investigating personal change in chronic illness or disability with metasynthesis of subset of articles focusing on transformation in primary care.
Context	Model is derived from a literature review (qualitative work) the theories of Mezirow: e.g. motivation to create meaning following a 'crisis', and critical thinking as a mechanism for doing so.
Intervention	Draws on adult learning theories of transformation.
Mechanisms triggered	This paper is focused on rehabilitation: model focuses on trigger phase, process of changing phase, and outcomes phase. The authors have modified this (see paper below) for application in primary care and chronic disease to initial phase, embracing the challenge phase and new ways of being phase.

	The model has not been empirically tested.
Reported outcomes	It argued that engaging in transformation models lead to new rules, ways or guidelines, new behaviours, new feelings, new beliefs, new perspectives and new sense of identity.
Authors interpretation	The trigger phase is described as a time in which the experience of being ill or disabled produces limits or losses, signs and symptoms, or a diagnosis is given, and/or challenging interactions occur with healthcare providers / systems. If this prompts readiness for change then it leads to critical reflection and a process of restructuring which can then lead into the outcomes above. Recognition of barriers to the process is made.
Strengths and limitations	This paper provides a theoretical foundation for adaptions which may occur in response to living with chronic disease(s). It identifies theories of transformation as being important for learning from lived experience (whether as a patient or professional).
Our interpretation	Readiness for change is emphasised as a crucial element to meet any of the outcomes above (defining success).
Use made of citation in synthesis (contribution to theory building)	Transformation ideas used to develop new model of concurrent learning for patients, learners (students and trainees) and professionals: all changing as a result of lived experiences of multimorbidity. Note taken to avoid assuming all transformation will be constructive.

**CITATION 42: Dubouloz, et al 2010b**	
Country of origin	Canada
Study design	Development of a model of the process or transformation in rehabilitation and application in chronic health conditions.
Context	Model derived from qualitative studies in the literature that sought to understanding personal change in chronic illness. Focus on patients with diabetes or HIV as exemplar illnesses in studies selected for meta-synthesis.
Intervention	This version of the authors' transformation model attempts to provide a conceptual framework for personal transformation within a primary care context for people living with chronic illnesses. Transformation is presented as a process of learning about oneself and the chronic illness in an iterative and continually changing manner.
Mechanisms triggered	Transformation conceptualised in the primary care context as an initial reaction followed by 2 distinct phases: embracing the challenge (critically reflecting on personal context and illness) and integration of new ways of being (changing perspectives leading to new understanding of self).<br><br>Although model not tested in the 'real world' authors identified a gap in the literature: further understanding needed of how interactions with professionals shape the process.
Reported outcomes	Suggests that professionals who understand how and why people change (transform) when living with chronic illnesses will be able to apply this model to identify needs and provide appropriate support for a constructive adaption.
Authors interpretation	The authors suggest that an initial phase (of up to 5 years) can follow diagnosis with a chronic illness, when both personal responses and the responses of others will shape what happens next. In response to challenges those ready for change will later engage in 'embracing the challenge' – acknowledging the disease, taking stock, exploring, experimenting and learning to develop adjustments in perceptions of self, activities and relationships. This can lead to integration of new ways of being that provide meaning to the person and allow them to have meaningful lives despite their morbidities.
Strengths and limitations	This is an evidence-based model although its function in practice has not been tested.
Our interpretation	Success is implicitly defined as creating new ways to experience a satisfying and meaningful existence despite presence of chronic illness.
Use made of citation in synthesis (contribution to theory building)	Used to inform 'learning' in the widest sense in the interactions between patients, defined learners (students and trainees) and professionals.

**CITATION 45: Ferenchick et al 1997**	
Country of origin	USA
Study design	Theoretical overview of strategies for teaching in ambulatory care.
Context	Medical students learning in ambulatory care.
Intervention	Facilitation of learner-patient interactions: seeks to offer practical advice on how to find time for this and provide appropriate support. Emphasises need for a three part model: planning, teaching and reflection. Also identifies need for learners to hold some responsibility for patient care.
Mechanisms triggered	Model not tested.
Reported outcomes	Argues that this prevents learning activities from impinging negatively on time resources.
Authors interpretation	Suggestions include phased timing of students seeing patients first ('the wave model') in order to prevent the logistics of time for learners conflicting with time pressures in care delivery, orientating students to patients' past problems, and presentation of findings.
Strengths and limitations	This is not a high quality paper due to the absence of testing but it highlights the need to resolve perceptions that learning activities are a drain on resources / in competition with patient care.
Our interpretation	Success described as greater learner-patient interaction.
Use made of citation in synthesis (contribution to	To sensitise ourselves to issue of time.

theory building)	

CITATION 46: Fernald et al. 2001	
Country of origin	USA
Study design	Medical student (n=171) focus groups (n=24) analysed to identify students perspectives on context and process in longitudinal primary care preceptorships.
Context	Medical students spending time in primary care.
Intervention	Model developed which emphasises the need for active engagement of learners, support and challenge, plus shared time for students to work with supervisors to reflect and make sense of experiences.
Mechanisms triggered	Focus groups used to identify how workplace-based learning could be improved. Students identified the importance of being engaged in meaningful activities, trusted and supported.
Reported outcomes	Learning appeared to be consequential to learner autonomy and responsibilities within supported parameters.
Authors interpretation	Students believed this model would allow them to develop new knowledge and skills, take on more responsibility, build relational learning and develop models for practice.
Strengths and limitations	Although not specific to multimorbidity this model mirrors others for generic learning in workplaces and links with transformative models of learning.
Our interpretation	Students needed to be able to develop models of practice that would serve future patients.
Use made of citation in synthesis (contribution to theory building)	Additional evidence of how students believe they are best supported to develop into practitioners of the future.

CITATION 52: Gabbay et al 2004	
Country of origin	UK
Study design	Ethnographic study of two general practices including 9 GPs, 3 nurses, 1 phlebotomist and other associated staff in the first practice and 3 GPs in the second.
Context	Non participatory observations and semi-structured interviews were conducted in GP practices over 2 years.
Intervention	Study sought to understand how primary care clinicians reached individual and collective healthcare decisions in practice.
Mechanisms triggered	Authors found that clinicians used 'mindlines' rather than explicitly referring to research evidence or other sources. Mindlines were defined as 'collectively reinforced tacit guidelines' - formed through interactions with other clinicians, patients, opinion leaders and others involved in healthcare. Identified this as the way knowledge was constructed and iteratively generated though social practices.
Reported outcomes	Authors suggest that given the social construction of mindlines it may be important to use networking as a mechanism to convey evidence to clinicians or bring about change.
Authors interpretation	Lived experiences are important sources of 'knowledge in practice'.
Strengths and limitations	A high quality qualitative study.
Our interpretation	Although no specific concepts of success or failure are offered the underlying theory that how people interact with each other to share knowledge in practice may be crucial to change is relevant and important.
Use made of citation in synthesis (contribution to theory building)	Used to refine theory of how social interactions influence learning and practice.

CITATION 55: Glasgow et al 2003	
Country of origin	USA
Study design	Policy publication advocating self-management in chronic illness.
Context	Model is developed by the authors who describe themselves as experts in the field of self-management interventions for patients and also draw on existing literature to inform their model.
Intervention	Goal was to describe a model of self-support applicable across different chronic illnesses and healthcare systems.<br><br>Underlying theory that self-management is necessary for change and collaboration between patients and professionals is required to achieve this. The model is said to require implementation at three levels: individual interactions, practice level (e.g. local service context) and systems / policy level.
Mechanisms triggered	The model is based around the concept of a 'Personal action plan' for each patient which details goals in behavioural terms, barriers and strategies to address these, follow-up plans and sharing the plan with professionals / those providing social support for patients. The development of these plans is said to arise from a 5 step process of: assess (beliefs, behaviours, knowledge); advise (specific information); agree (goals); assist (changes in behaviour) and arrange (progress monitoring).<br><br>This model is reported to derive in part from experience of real life practices but the derivation and mechanisms within it have not been prospectively tested.
Reported outcomes	Anticipated consequences are greater satisfaction for patients all involved in chronic illness care.
Authors interpretation	Model has not been tested.
Strengths and limitations	Difficult to judge from information presented. Much of the model has 'common sense' validity and it highlights that self-management is a popular contemporary approach to chronic illness care at present.

Our interpretation	Clear or specific outcomes not given beyond shared decision making and goal setting. Failure is conceptualised as not working in partnership with patients.
Use made of citation in synthesis (contribution to theory building)	Note made of the role of perceptions of self-management in current thinking about chronic illness.

**CITATION 60: Haidet et al 2006**	
Country of origin	USA
Study design	Narrative analysis of semi-structured interviews with primary care patients (n=16).
Context	Patient narratives collected from 16 open ended qualitative interviews with primary care patients in Texas.
Intervention	Better understanding of patient illness narratives can inform practitioners seeking to negotiate patient participation in shared care. The authors developed a model of patient participation that suggests patient perspectives on the centrality of illness and potential to change will interact with their willingness to engage in illness activity and partnership with professionals but importantly both these are subject to the influence of more or less productive strategies for coping.
Mechanisms triggered	Identified themes: Perspectives of illness (centrality to life, changeability); actions pursued (illness related activity, partnership in decision making and illness management).<br><br>Within this there was evidence of variability between patients and their expectations. Identified strategies used by patients for coping with illness ranged from passivity/fatalism, unguided searching, adherence, self-motivated change to negotiated empowerment.
Reported outcomes	The authors conclude that improvisation is an essential skill for professionals to develop effective and individualised ways of working with patients.
Authors interpretation	Overall it is suggested that communication skills need to be developed so that practitioners can hold unscripted conversations with patients.
Strengths and limitations	Suitable qualitative methods used.
Our interpretation	The authors place 'passivity/fatalism' at the failure end of a spectrum with 'negotiated empowerment' as the successful polar opposite. They argue this applies to communication and illness care.
Use made of citation in synthesis (contribution to theory building)	Used to refine ideas about how interactions are negotiated and influences on these.

**CITATION 67: Jackson et al 2008**	
Country of origin	Australia
Study design	Theoretical model for primary care.
Context	Considers all patients registered with a GP and the learning / development needs of qualified doctors to meet the needs of patients and learners.
Intervention	Describes the 'Primary Care Amplification Model' to create 'beacon practices' around which primary care activities can be undertaken collaborative and extended roles for primary care developed. Describes pilot of model in practice: 4 key features are (1) supporting primary care within and external to the practice (2) expanded clinical model of care (3) governance directed to meeting local community needs and (4) appropriate infrastructure.
Mechanisms triggered	Focus on service organisation and supporting activities. No details are given about the realities of this beyond noting the following challenges to implementation: the need for a clear and shared vision, getting a strategic fit with governance partners, identifying a skilled and supportive executive with clinician leadership and engagement of local practices and communities.
Reported outcomes	No outcomes or information about impact on patients or learners is given.
Authors interpretation	The authors describe this as a model with potential that fits well with policy drivers to increase community-based and community led care.
Strengths and limitations	This is an interesting model but it does not appear to have been evaluated in practice, rather the paper presents a description of the model as designed 'on paper'.
Our interpretation	No clear definitions given.
Use made of citation in synthesis (contribution to theory building)	Consideration of the key elements in developing our ideas.

**CITATION 70: Jordan et al 2009**	
Country of origin	USA
Study design	Theoretical analysis of the role of conversations in health care interventions.
Context	Theoretical paper which draws on the authors experiences of observational and implementation studies in primary care in tandem to sociolinguistics literature and complex adaptive systems theory.
Intervention	Purpose of paper was to explore the role of conversation in healthcare interventions as a means of making sense and learning.
Mechanisms triggered	Authors acknowledge that implementation in healthcare of new interventions often produced unexpected outcomes. They suggest this is partly due to conversations between people, and therefore, greater attention

	should be paid to conversation as a social interaction mediating implementation in practice.
Reported outcomes	Argues that across implementation activities variable conversations occur leading to varied sense-making, learning and unexpected outcomes. Without this understanding then it is not possible to achieve sustained change in primary care. Highlights the need to modify belief as well as action to produce learning and change.
Authors interpretation	Social interactions need further study to develop implementation science. Suggests greater use of complex adaptive theory can help with this. Suggests that those seeking to implement new interventions should consider the role of collaborative and improvised conversations, how to emphasise qualities that harness conversation for constructive learning and build opportunities to use social interaction into the implementation plans.
Strengths and limitations	Although not specific to multimorbidity this paper draws on relevant theory and empirical data generated in the context of primary care: sense-making is important for patients, learners and doctors and will occur through their conversations and social interactions.
Our interpretation	The active creation of space for conversations in which shared learning can be developed and people can work collaboratively.
Use made of citation in synthesis (contribution to theory building)	Used to draw attention to the importance of language, conversation and social interaction which leads to meaning making for everyone involved.

**CITATION 75: Klinkman et al 2011**	
Country of origin	USA/Netherlands
Study design	Theoretical discussion of person-centred diagnosis in general medicine.
Context	Discusses the infrastructure needed to provide patient centred care (rather than disease-specific approaches).
Intervention	Describes a theoretical 3D matrix model of patient assessment: social, psychological and medical needs all contribute to the burden of illness in multimorbidity. Draws on the concept of the patient-centred medical home (ref) including an on-going personal relationship with a doctor.
Mechanisms triggered	Although not tested in 'real' practice the authors highlight that time (and undervaluing of time) is a major challenge to provision of this model in current healthcare systems. Identifies risk of 'satisficing' when time is pressured.
Reported outcomes	Intended consequence is more holistic care with GPs and primary care practices acting co-ordinating hubs for patients with multimorbidity. Risks are discussed, particularly time pressures, but the model has not been tested in practice.
Authors interpretation	Identifies the need to ensure that person-centred care is facilitated by evolving technologies, rather than these becoming the driving force of change in ways that produce disease technology but without clear patient benefit.<br><br>Suggests that this should be achieved by combining inputs from patients, clinicians and routine data to assess person, problems, clinical modifiers, actions, timescales, and further data generated should be looked at longitudinally as well as in cross-section for individual patients.
Strengths and limitations	There is little new in this paper but it brings together several ideas which are themes in the literature to suggest a model of working. It would be challenging to get ownership in practice due to pressures of time and / or professionals perhaps thinking they already do what they can.
Our interpretation	Patient-centred care is seen as success while on-going focus on disease-specific details and technology in isolation are seen as failures of the current system.
Use made of citation in synthesis (contribution to theory building)	Used to refine emphasis on interpersonal working.

**CITATION 81: Loffler et al 2012**	
Country of origin	Germany
Study design	Grounded theory analysis of in-depth interviews with multimorbid patients (n=19).
Context	Patient group studied were aged 65-85. 13 were female and 6 male. All had at least 3 chronic conditions including one musculoskeletal condition, and had visited the GP at least once in the preceding three months.
Intervention	Draws on concepts of coping – defined as efforts to manage demands on the resources of a person, a process of thinking and action. Within the patient group distinguished between emotional coping (when it is believed that nothing can be done to change the situation) and problem solving focuses of coping which occurred when there was an expectation of change.
Mechanisms triggered	Identified social, emotional and practical coping strategies among participants. Social coping included finding ways to maintain a meaningful life and keep autonomy, maintain social roles or find new ones if physical functioning changed and to have a 'can-do' attitude to life.
Reported outcomes	Each of the three strategies could be used to contribute to the patient being pro-active in their behaviour and interactions in healthcare.<br><br>SOCIAL: keeping a social role, sustaining a meaningful life and self-determined choices of who to ask for support all contributed to preserving autonomy and having a can-do approach.

	EMOTIONAL: putting effort into sustaining abilities and realising desires and dreams were important to give a sense of pride, strength and well-being which contributed to positive approaches to life.<br><br>PRACTICAL: controlling existing diseases and informing oneself actively about disorders and therapeutic actions, awareness of one's own body and prioritising medications shaped the patients' approaches to active disease management. Sometimes this meant less adherence to medication recommendations, through active choice.
Authors interpretation	Patients generally held positive attitudes towards life, and tried to preserve autonomy at a social level. Emotional oscillation between anxiety and strength was identified while patients sought practical ways to keep diseases under control. These patients were often critical of medication, rather than passive recipients.
Strengths and limitations	High quality qualitative research study
Our interpretation	Success is defined as collaborative working with patients to develop pro-active behaviour rather than seeking to enforce disease-specific protocols.
Use made of citation in synthesis (contribution to theory building)	Highlighted the importance of recognising lack of adherence to medical models and strategies is not necessarily due to disinterest or passivity but may be a result of active choice. Individual elements of the findings used to refine new model.

**CITATION 88: Martin et al 2002**	
Country of origin	Canada
Study design	Thematic analysis of interviews with GPs and policy makers (n=18).
Context	Qualitative analysis of telephone interviews about chronic illness care and their responses to examples in the form of clinical vignettes.
Intervention	None made explicit.
Mechanisms triggered	GPs reported that their main responsibilities were to address presenting problems (i.e. addressing the immediate) and managing disease and treatments. It was unclear whether individualised care was something this group really believed in or simply paid lip-service to as current policy.<br><br>It appeared that experience and education shaped care models as much as reforms and incentives.
Reported outcomes	GPs reported that holistic models of care encompassing preventative, psychosocial, and care coordination could only be used if time permitted.
Authors interpretation	Authors identified the need to further understand the dynamics of care at practice and individual level.
Strengths and limitations	Difficult to assess due to lack of detail but the authors may have overstated their findings due to a fairly simplistic thematic analysis which is descriptive rather than interpretative. Nonetheless the issues raised resonate with other literature.
Our interpretation	Not addressed.
Use made of citation in synthesis (contribution to theory building)	Minor role in considering the GP perspective.

**CITATION 89: Morris et al 2011**	
Country of origin	UK
Study design	Longitudinal semi-structured interviews with patients who had multiple long term conditions (n=30 recruited of whom 21 had multiple conditions)
Context	Qualitative study embedded in a RCT (aiming to improve self-management support). Semi-structured interviews were conducted to see what influenced prioritisation in self-management and how this changed over time. 21 people with at least one chronic condition were interviewed.
Intervention	Theoretical model produced from thematic analysis of interviews. Four sorts of factors were found to influence the impact of having multiple conditions on self-management:<br>1. Disruption by conditions (lack of engagement, separation of conditions, confusion, being overwhelmed, uncertainty)<br>2. Accommodation of conditions (continuity from existing illness behaviour / integration with existing practices, control over conditions and symptoms, enough understanding of conditions, confidence)<br>3. Factors influencing the shift from accommodation to disruption (exacerbations, confusion and contradictory information, events, loss of control, medication)<br>Factors influencing the shift from disruption to accommodation (taking control, links between existing knowledge and experiences, adapting information and practices into new routines, interaction with health professionals).
Mechanisms triggered	The model above was produced from the real life experiences of patients but has not been prospectively tested for improving self-management through emphasis on constructive and mitigation of destructive influences.
Reported outcomes	Authors suggest that clinicians might usefully engage with patients' understanding to reduce complexity and enhance engagement. It is important to note that the patients in this study sought to make new diagnoses minimally disruptive to existing management plans.
Authors interpretation	Prioritisation occurs and allows patients to manage their illnesses; specific priorities change with time and impact of each illness. Patients could benefit from discussion of priorities and better information to aid

	their prioritisations.
Strengths and limitations	High quality qualitative study.
Our interpretation	The study assumes that one facet of success is the ability to self-manage chronic disease.
Use made of citation in synthesis (contribution to theory building)	Consideration of how the identified influences form part of social interactions between patients, learners and professionals.

**CITATION 99: NHS Improvement 2012**	
Country of origin	UK
Study design	Policy document for long term conditions.
Context	This is a policy document for the management of long term conditions in the NHS.
Intervention	Suggests there are four elements within a pathway approach: stabilising the condition to get patients back to living their lives, assisting patients to live their lives with support provided through monitoring and review, timely intervention to the appropriate service when things go wrong, and providing choice and support towards the end of life.
Mechanisms triggered	Examples are given from different NHS trusts although these are not analysed or presented in any detail and different trusts are used to provide example of each of the elements (when in practice all four will be needed in each place). The mechanisms which led to successful implementation of change are not made explicit. The need for service coordination in real practice is identified.
Reported outcomes	Recommends seven day working in the NHS to achieve good services within each element.
Authors interpretation	A policy recommendation is made to move away from organisational models of delivery to pathway approached that focus on the needs of individual patients.
Strengths and limitations	This illustrates policy drivers and responses in this context but it does not provide robust evidence of change.
Our interpretation	Success is conceptualised as less variation in patient care between organisations but also as more individualised care. This tension is not resolved. Other markers of success given include: self-management, use of technology to support rehabilitation, partnership working, remote monitoring, shared care, reducing hospital admissions, advance care planning and access to information.
Use made of citation in synthesis (contribution to theory building)	Acknowledgement of trajectories of long-term conditions (in the context of multimorbidity) and need to address different elements.

**CITATION 105: Parboosingh et al 2011**	
Country of origin	USA
Study design	Theoretical discussion of practitioner interactivity and its impact on learning.
Context	Focus on continuing professional development in general practice.
Intervention	Argues that learning is the product of interactions between individuals in trusted relationships, therefore, in continuing professional development it is important for practitioners to be engaged in communities of practice.
Mechanisms triggered	Suggests that practice improvement initiatives would be more successful in producing behavioural change if networking and interactivity were used to develop emergent learning among practitioners.<br><br>The importance of trust and relationships is emphasised although no tested in practice.
Reported outcomes	Learning relevant to practitioners.
Authors interpretation	Primary care is a complex adaptive system and social processes should be studied to maximise the potential of improvement initiatives.
Strengths and limitations	These are interesting theoretical ideas which need further study in practice.
Our interpretation	Successful learning is conceptualised as emergent learning: intuitive generation of knowledge with the potential for practice improvements linked to evidence to develop customised solutions to complex problems.
Use made of citation in synthesis (contribution to theory building)	Importance of networking, trust, relationships and emergent learning incorporated into new model.

**CITATION 112: Russell et al 2008**	
Country of origin	Canada
Study design	Phenomenological evaluation of effect of external facilitators to enhance delivery of chronic condition care in primary care.
Context	Patients (n=20) with chronic illnesses and their physicians (n=13) were interviewed following participation in a RCT. The RCT sought to investigate the effect of external facilitators (n=3) in enhancing delivery of chronic condition care planning in primary care. Three study facilitators were also interviewed. Physicians received financial incentives for completion of care plans.
Intervention	Participants in the active arm of the RCT were required to produce jointly formulated comprehensive individual patient care plans. Nurse facilitators visited practices monthly. The study looked at a model of 'Chronic Illness Care Management' which consisted of: a written care plan developed between the patient and physician that included the patient's goals, medication review, education and self-care plans, psychological

	and social assessment, community integration and social support and prevention activities.
Mechanisms triggered	Family physicians viewed chronic illness management as predominantly about biomedical concerns and with the exception of a few enthusiasts were reluctant to consider non biomedical factors. The enthusiasts reported the process was useful for provision of holistic care. Some physicians did not think the systematic care plans differed to existing care or did not think patients were capable of collaborative working and/or thought care planning should be a nursing rather than medical task. Many patients did not notice any attempt had been made to change their care although those who did gave positive reactions.
Reported outcomes	Barriers to change were identified including the new model of working either being rejected by physicians or not recognised as being significantly different to current practice. Rejection was based on what physicians perceived to be their role and how they perceived their interactions should be with patients.
Authors interpretation	Authors identified the need for individual ownership of change in practice.
Strengths and limitations	Good qualitative study.
Our interpretation	The study designers' conceptualisations of success differed from participants. The latter had a narrower view of the role and responsibilities of a doctor and less expectation that patients would engage in self-management.
Use made of citation in synthesis (contribution to theory building)	Used to refine ideas about learning and dynamics between professionals and patients.

**CITATION 114: Sagasser et al 2012**	
Country of origin	Netherlands
Study design	Phenomenological study of interviews with GP trainees (n=21).
Context	Postgraduate GP trainees were interviewed to identify what helps or hinders in self-regulation of learning.
Intervention	Self-regulation was defined as monitoring of performance, identifying domains for improvement, undertaking learning activities, applying new knowledge or skills and self-assessing performance.
Mechanisms triggered	Trainees reporting two forms of self-regulation:<br><br>Short loop: over a week at the most and focused on easy-to-solve problems / minor learning activities.<br>Long loop: complex and recurring problems which needed multiple planned longitudinal learning activities. This loop was influenced by external assessments and formal training.<br><br>Supervision in practice was considered to facilitate both loops. Self-confidence was used to gage competence, learning was motivated by personal desire to be a good doctor as well as interpersonal stimulation.
Reported outcomes	Self-regulation is often described as a theoretical model so these authors sought to understand if and how it occurs in practice. Key elements of self-regulation were identified including: reflection on daily practice, benchmarking knowledge, seeking learning opportunities, opportunity to put learning into practice. These were influenced by personal, interpersonal and contextual factors.
Authors interpretation	Authors conclude that trainees need to be encouraged to seek external feedback and supervisors need to be supported to ensure that they take an active and critical role in encouraging reflection as well as acting as role models. Further consideration is needed of how external influences shape learning in practice.
Strengths and limitations	High quality qualitative methods.
Our interpretation	This paper is not specifically about multimorbidity. However, much of the learning required is likely to occur in practice with learners developing their conceptualisations of success and failure from their experiences and interactions with others. Learning about multimorbidity and care is likely to occur through the processes described, particularly with respect to issues such as management of uncertainty which require experience as well as knowledge.
Use made of citation in synthesis (contribution to theory building)	Used to refine how learning occurs in practice.

**CITATION 117: Schuez et al 2012**	
Country of origin	Germany
Study design	Modelling of patient illness representations and relationships between these.
Context	Questionnaire study of illness perceptions among 305 people aged 65 or older with at least two conditions mentions in the Charlson comorbidity index.
Intervention	Discusses self-efficacy and the 'common sense model' (ref). The common sense model assumes that individuals form subjective representations about their illnesses which in turn guide cognitive and behavioural responses.
Mechanisms triggered	Suggests that strengthening self-efficacy may improve illness controllability beyond the effects of illness-specific information.<br><br>Found that as people lived with an illness their perceptions of control over its outcomes increased. Effective treatment control also increased their sense of personal control, with distress associated with loss of treatment control.
Reported outcomes	Identity, causes, timeline, potential for cure/control, consequences and emotional responses all affected perceptions of illness.

Authors interpretation	Authors conclude that these findings offer better understanding of patient experiences and coping strategies for living with chronic illness. The results suggests that variability is due to person-level factors i.e. illness representations of different illnesses in the same person are similar while illness representations of the same illness in different people are different.
Strengths and limitations	Good quality paper. Further work is needed to see if person level factors can be targeted to improve coping strategies and living with multimorbidity.
Our interpretation	Highlights that personal belief systems and characteristic influence how people think about illness and health as well as context.
Use made of citation in synthesis (contribution to theory building)	Incorporation into the new model of the importance of understanding individual patients' conceptualisations of their illnesses.

**CITATION 126: Soubbi et al 2010**	
Country of origin	Canada/Netherlands
Study design	Theoretical model of primary care practice for patients with multimorbidity.
Context	Chronic disease management in primary care. Suggests that this cannot be achieved using disease-specific approaches and / or acute care models (just addressing immediate needs).
Intervention	Theoretical development of a model for care and practice in this paper is based on taking a communal and dynamic view of responses to chronic illness. Suggests this should incorporate attention to the co-creative nature of illness response, integration of the skills and resources of all participants (patients, professionals and families). For professional learning suggests that there is a need to design interventions which can harness emergent learning and practice that occurs from interactions with patients.
Mechanisms triggered	Model has not been tested in practice but it is suggested that trust, communal engagement and openness to experimentation will be necessary to facilitate collective learning.<br><br>Draws on complexity theory and communities of practice as models for social interactions that can lead to learning from patient experience as well as sharing evidence-based medical approaches. Suggests that a learning community can be formed through the interactions of patients, health professionals and families through consideration of each group's behaviour, skills, cognition and emotion.
Reported outcomes	The intended consequences are:<br>1. A shift from disease management to illness management – the latter being how people perceive, explain and cope with disease progression<br>2. Accounting for dynamic complexity in length and multifactorial chronic illnesses when tailoring adaptive management approaches to individual patients.
Authors interpretation	In rejecting the idea of a standardised framework of care delivery based on abstract evidence for specific disease management this paper offers an alternative vision whereby the principles of shared learning, collaborative practice and emergent care through community interaction are used to develop individualised chronic illness management approaches. Specific suggestions include: creating time and space to discuss care (with an educational focus using experiential learning techniques), making trust and compassion a strategic requirement, including emotional aspects, co-creation of care plans, identification of key activities depending on individual and illness, use of narrative medicine, flexible support, incremental change towards goals, challenging assumptions and recording the learning process.
Strengths and limitations	This is a well argued, theoretically grounded model but it has not been tested in practice.
Our interpretation	Success is conceptualised as collaborative working between patients, families and professionals to achieve health outcomes 'within the evolving opportunities and constraints of chronic illness'.
Use made of citation in synthesis (contribution to theory building)	Used to develop interpersonal interaction aspects of new model and to develop ideas about emergent learning for all participants in workplace-based education and care delivery.

**CITATION 127: Soubhi et al 2007**	
Country of origin	Canada
Study design	Theoretical model of primary care practice for chronic illness care.
Context	Patients with multimorbidity in primary care. Professionals learning to care for them (main focus is continuing professional development rather than students / trainees).
Intervention	Aim was to develop mechanisms for collaborative learning which would improve primary care for people with multimorbidity.<br><br>Proposes a model of multimorbidity management involving communities of healthcare providers to develop collective learning from their experiences. Model emphasises the importance of relationships between professionals for delivery of patient-centred care and the need for flexible working with continuous reassessment.
Mechanisms triggered	Draws on theories of communities of practice, clinical microsystems and mindlines.<br><br>Model not tested in practice but it is suggested that professional 'know-how' developed in practice from experience of interactions with patients can be harnessed through professionals sharing learning and working together to develop solutions, implement strategies and care plan and evaluate the effects of

	these which will lead to new learning.<br><br>Suggests key to the model working will be willingness among participants to learn from shared practice, further each other's goals, share stories of success and failure and promote continued evaluation of collective learning.
Reported outcomes	Intends to bring about collaborative working through which the challenges of multimorbidity can be met – going beyond disease specific approaches and clinical guidelines.
Authors interpretation	Presents a theoretically derived model of working and argues that this is particularly pertinent to multimorbidity in primary care due to the complexity of individual patients' circumstances and care needs.
Strengths and limitations	Well-developed theoretical model with identification of potential issues in practice, although the model has not been tested.
Our interpretation	Success is defined as continuous collaborative working and learning to achieve best practice.
Use made of citation in synthesis (contribution to theory building)	Ideas used to refine model with respect to experiential learning that occurs in practice.

**CITATION 128: Stewart et al 2002**	
Country of origin	New Zealand
Study design	Evaluation and research data from trainees analysed using a model of practice based education.
Context	Trainees in general practice.
Intervention	Paper looks at evaluation data from trainees generated through written feedback at the end of training placements (n=372) and focus groups (n=2) with trainees (n=16). Draws on Shipengrover and James' model of quality in general practice (ref).
Mechanisms triggered	Trainees emphasised their need for support and scaffolding of learning during general practice placements. A 'black box' was identified with respect to the processes that might lead to satisfied learners who understand general practice and are then equipped to provide community-based comprehensive care. Learners suggested these processes were dependent on themselves, GP trainers, practice environments and patient contact.
Reported outcomes	Informal learning occurs as the result of interactions with patients and GP trainers – how, why and what this consists of needs further study.
Authors interpretation	The authors demonstrate how learner evaluations and perceptions linked to a model of learning in general practice.
Strengths and limitations	Although this paper provides useful information the research rigour could have been improved by generation of more data (very limited data is presented). The analysis is descriptive and set in a particular context, but it does echo other findings in the literature.
Our interpretation	A high quality practice for learning was identified as one where the learner had access to adequate numbers of patients with a range of problems, trainers were available and approachable, informal discussions occurred, there was a supportive working environment and trainers demonstrated high quality practice.<br><br>Conversely learning failed when there was a lack of explicit goals, teaching opportunities were interrupted, feedback was not specific or constructive, there was a lack of opportunity to see patients with chronic diseases and other practice staff were unsure of the learner's role.
Use made of citation in synthesis (contribution to theory building)	Consideration of inputs, process issues and intended consequences in developing learning experience aspects of model.

**CITATION 130: Swanick et al 2006**	
Country of origin	UK
Study design	Theoretical model of specialist training in general practice.
Context	Postgraduate trainees in general practice.
Intervention	Draws on theories of experiential learning, apprenticeship and legitimate peripheral participation in addition to Millers pyramid of clinical competence (ref).
Mechanisms triggered	This paper does not report the testing of these theories in practice although others have done so and drawn the same conclusion; that is participation is key. The need for support to turn experience into learning is also highlighted.
Reported outcomes	Not addressed.
Authors interpretation	Essentially the purpose of the paper is to argue that designating learners as 'supernumerary' can be counterproductive due to the potential for this to lead to lack of opportunity or engagement for supervised practice.
Strengths and limitations	This is an example of a theoretical presentation of well-known learning models being applied to medical education but the ideas within it are untested (by this author).
Our interpretation	Highlights that responsibility can (perhaps should) come hand in hand with learning rather than following sequentially.
Use made of citation in synthesis (contribution to theory building)	Strengthens concept of participation already identified in more robust literature.

CITATION 138: Wearne et al 2012	
Country of origin	Australia/Netherlands
Study design	Literature review of empirical evidence, descriptions and recommendation for supervision in general practice.
Context	GPs as supervisors of postgraduate trainees in general practice.
Intervention	Review of the literature to develop a model of supervision. Draws on workplace-based learning theories and model is developed using a sociocultural perspective.
Mechanisms triggered	Identified that in the literature recommendations and opinions outweighed empirical evidence, however it could be seen that in practice supervisors intertwined clinical and educational activities. When an 'educational alliance' was formed between supervisor and trainee (due to interpersonal connections) this provided a good foundation for learning.<br><br>Found evidence that supervisors felt they had to juggle priorities to ensure patient care was not jeopardised by trainee learning. There was no evidence identified to clarify whether this was a problem or not.<br><br>Trainees wanted challenge as well as support.
Reported outcomes	Identified that too much supervision can lead to suboptimal development due to lack of interest as well as too little having the same effect due to trainees despair. Supervision is about trying to maintain balance for optimal development.
Authors interpretation	Model suggests that the expert clinician has responsibility for patient safety during the learning process: they need to organise the learning environment, assess trainees learning needs, facilitate learning (through provision of resources, promotion of reflection, provision of feedback, and role modelling), monitor learning, attend to trainees well-being and summarise learning.
Strengths and limitations	High quality interpretative review of the literature which has led to the development of a new theoretical model.
Our interpretation	Success is defined as 'just the right amount' of responsibility – the supervisor needs to have a relationship with their trainee that allows the development of trust with respect to handling risk while ensuring patient safety to produce optimal learning.
Use made of citation in synthesis (contribution to theory building)	Used to develop ideas of relationship-centred learning in new model.

CITATION 140: Yardley et al 2012	
Country of origin	UK/Netherlands
Study design	Theoretical overview of educational theory relevant to learning from experience.
Context	This paper provides an overview of experiential learning theories applied to undergraduate and postgraduate medical education. It contains a concentric model for learner support and highlights transitions in experiential learning time during the continuum of medical education.
Intervention	Draws on sociocultural perspectives to underpin learning theories: learning is situated, can be viewed as an individual and/or collective process, and learning is triggered by authentic practice-based experience.
Mechanisms triggered	Experiential learning theories identified as having real world resonance with workplace-based learning in medicine. Social interactions shape learning, and although learning can be triggered by experience support is needed to maximise learning potential. A core condition of learning is participation.
Reported outcomes	Learning will vary from individual to individual and is a product of their cognitive, emotional, personal and collective sense-making activities as a result of experiences in practice.
Authors interpretation	Sociocultural and experiential learning theories can explain workplace-based learning: transitions occur in medical education as learners develop increased independence and self-direction in their learning and more of their time is spent learning 'on the job'. Further work is needed to fully understand these processes and what the consequences of on the job learning are.
Strengths and limitations	The first author of this paper is a member of the current research team. The paper was independently appraised by other team members who noted that it provided good working examples of experiential learning within medical education and included critique of the use of these theories.
Our interpretation	Successful learning comes from the learner having a legitimate role in practice, receiving constructive feedback, and active engagement in considering contextual factors and possibilities for transfer. Past experience can potentiate or limit new learning and must be considered.
Use made of citation in synthesis (contribution to theory building)	The principles of experiential learning theories are not dissimilar to those of transformative / assimilation of change theories in patient focused literature. Both were used to develop the ideas within our model regarding learning in the broadest sense of making meaning and constructing knowledge whether as a learner, patient or professional.

Other empirical interventional studies

CITATION 36: Dent et al 2010

Country of origin	USA
Study design	Paired pre- and post- 4 week community visit / chronic disease management project survey of medical students (second year).
Context	N=170 matched surveys (out of 219 potential)<br>Curricular intervention was designed to prepare students to integrate practice with an understanding of the community context of patients. Rural and medically underserved sites were used.
Intervention	Used a Chronic Care Model framework to encourage students to consider interactions between patients and professionals in the context of the community in which they were based and to identify health system resources available to support improvements in health in the context of chronic diseases. Each student had to identify one patient to work with exploring the burden of disease and developing a self-management plan under the supervision of the patient's doctor.
Mechanisms triggered	Importantly these were not assessed.
Reported outcomes	Post assessment items showed statistically significant differences in knowledge with respect to community responsiveness, some change in attitude and an increase in reported intent to practice in community-oriented healthcare settings. Responses were rated on a 4 point Likert scale using a previously developed questionnaire in modified format.
Authors interpretation	Exposure to community settings can develop competencies needed for future practice e.g. models of chronic care.
Strengths and limitations	Intervention still focused on single diseases and reductionist indexing models. Survey did not use a validated scale. Given that the intervention was embedded in a 4 week placement with other activities cause and effect cannot be established. Study was uncontrolled.
Our interpretation	The findings that exposure increases understanding are as one would expect but the study is not robust enough to conclude more than this.
Use made of citation in synthesis (contribution to theory building)	Noted that learning from experience is of interest to medical educators in designing curricula for learning about chronic illness.

**CITATION 80: Leykum et al 2011**	
Country of origin	USA
Study design	Scale development and administration to inform baseline assessment in an interventional study of chronic care models.
Context	Primary care clinics (n=40) in South Texas: clinic clinicians and other staff (n=296) participated in a survey using a newly developed scale of learning and a pre-existing scale of assessment of chronic illness care.
Intervention	The aim of the study was to better understand learning in primary care practice. Drawing on complex adaptive systems frameworks the authors consider that implementation efforts need to take account of dynamic systems as well as seeking to improve care through education and decision support for individual providers. Learning scale contained 22 items with a 5 item subset focused on reciprocal learning. This subset showed reciprocal learning was strongly associated with implementation of chronic illness care models.
Mechanisms triggered	The authors suggest that each practice needs to be considered as a clinical microsystem to understand its dynamics and culture.<br><br>Reciprocal learning is suggested as a mechanism to bring about improvements. In the learning scale this was defined as scoring positively on 5 items: I am frequently taught new things by other people in this clinic, I learn a lot about how to do my job by talking to other people in the clinic, when we have a problem in this clinic we tend to examine it carefully so that we can come to an understanding of the problem and why it occurred, in this clinic we frequently learn about new things together as a group, I learn how to do things in this clinic by sharing knowledge with team members.<br><br>This suggests social interaction is an important mechanism for further study but within this paper the findings are limited to work validating a new learning scale for face validity in practice rather than testing it prospectively.
Reported outcomes	It is suggested that further work is needed to understand clinical microsystems and the impact these can have on attempts to implement new models of working, bring about learning and improved practice.
Authors interpretation	Authors suggest that reciprocal learning is an important attribute of learning in primary care clinics in the context of seeking to improve chronic illness care. They suggest developing models to do this will improve the care of patients with chronic disease and may also generally improve the clinic performance.
Strengths and limitations	It is difficult to tell from the methods section of this paper how much can be concluded from the statistical evidence with respect to the overall findings rather than simply the validation of the learning scale to measure learning. Further work may be needed to demonstrate the finding that reciprocal learning is associated with chronic care improvement recognised by patients rather than simply the implementation of a new model, although this does resonate with other qualitative work.
Our interpretation	The importance of social interaction and understanding dynamic practice is highlighted. Further work is needed to explore this and develop it with a view to new interventions or improvement initiatives.
Use made of citation in synthesis (contribution to theory building)	Ideas used to develop model of concurrency between learning and practice.

CITATION 87: Marsteller et al 2010	
Country of origin	USA
Study design	Cluster randomised trial of 'guided care': a specially educated registered nurse works with up to 5 GPs in a practice performing 8 clinical activities for up to 60 chronically ill older patients.
Context	14 Practices were randomised over a three year period to receive either guided care or usual care (49 doctors in total). Patients were over 65 years (650 in total).
Intervention	Doctors were surveyed at baseline and a year later to measure satisfaction with chronic care processes, time spent on chronic care, knowledge of their chronically ill older patients and care coordination between them and other staff.<br><br>8 activities undertaken by the nurse were: assessing the patient at home, creating an evidence-based care guide and action plan, monitoring and coaching the patient monthly, coordinating clinical interactions, smoothing transitions between sites of care for patients, promoting patient self-management, educating and supporting family caregivers, and facilitating access to community resources.
Mechanisms triggered	Not explored.
Reported outcomes	Practice doctors receiving guided care had statistically significant higher ratings of satisfaction with patient/family communication, and greater knowledge of the clinical characteristics of chronically ill older patients. No other statistically significant differences were found.
Authors interpretation	Authors were disappointed not to see greater differences between the intervention and control groups.
Strengths and limitations	A significant limitation is the lack of the patient perspective when assessing success or failure of this intervention. Also no comparison is made of clinical outcomes such as preferred place of care, or numbers of admission to hospital. The mechanisms at play were not explored.
Our interpretation	The limitations in how this intervention was evaluated suggest there was a missed opportunity to learn more about how and why this intervention played out in practice.
Use made of citation in synthesis (contribution to theory building)	Need to consider all perspectives noted in model development and look at a broader range of outcomes.

CITATION 96: Muir 2007	
Country of origin	UK
Study design	Descriptive report of undergraduate teaching based on focus group conducted for evaluation.
Context	Describes the evaluation of an experiential learning intervention for undergraduate medical students: 10 self-selected year one students participated in a focus group.
Intervention	Students were sent in pairs to see patients at home with the purpose of learning about patient experiences over time, and community-based care. Intervention was supported by GPs, community health workers and other volunteers. Students continued to visit their patient over the first three years of the course (5 times in total) and were encouraged to stay in contact beyond that time.
Mechanisms triggered	Little data from the students is offered and the mechanisms behind the intervention are not explored.
Reported outcomes	Students reported that it was beneficial to see 'real life' in a home context for these patients, that the visits gave them a better understanding of patient-centred medicine and an understanding of the patient curriculum. They also valued the continuity of contact.
Authors interpretation	Intervention was positively received.
Strengths and limitations	Only first year students were engaged in the evaluation so longitudinal element is not described or evaluated. Focus on awareness of patient experience seems to exclude other potentially relevant learning aims. Study was uncontrolled.
Our interpretation	As a descriptive study this provides evidence that experiential learning interventions can be successfully implemented but there is no in-depth analysis of the consequences. Student learning about chronic illness is not discussed in detail.
Use made of citation in synthesis (contribution to theory building)	Provided further evidence of the need to address how and why experiential learning functions in clinical practice.

CITATION 98: Nieman et al 2011	
Country of origin	USA
Study design	Descriptive evaluation of a primary care training programme for chronic illness education. Assessment of learning was made using an objective standardised clinical exercise (OSCE) to evaluate accuracy of differential diagnosis and communication skills.
Context	47 postgraduate trainees completed the programme, using the cases of 414 patients with chronic illnesses.
Intervention	Programme consisted of 4 parts: measurements of the health-related quality of life for patients with chronic illness (n=414), didactic sessions (n=47) in which trainees described chronically ill patients and their care, written narratives describing trainees' reactions to these patients (n=93) and portfolios of evidence of chronic illness learning.<br><br>Based on argument that trainees need to understand patients' perceptions of chronic illness and how this can affect their mental and physical functioning as chronic illness is an increasing priority in healthcare.
Mechanisms triggered	Although the authors describe each part of their intervention there is insufficient detail to identify 'real life'

	mechanisms at play. All the trainee reporting made is in the context of being assessed and the influence of this is not critiqued.
Reported outcomes	OSCE results are reported as ‘accurate’ skills but scoring is not given other than for participation. Trainers also rated portfolios as excellent (n-13) or satisfactory.
Authors interpretation	Findings suggest this was an effective way to promote chronic illness learning among trainees.
Strengths and limitations	This study is limited in that much of the intervention required taking trainees out of routine practice rather than focusing on learning integrated with clinical practice. The intervention activities may all support learning but it is unclear if trainees and trainers will have time or motivation to continue beyond the study. The outcomes are not reported in detail and there is little critique of the potential for longer term impact or not. In addition the study was uncontrolled.
Our interpretation	More time and supporting activities may improve focus on learning to provide care for people with chronic illness.
Use made of citation in synthesis (contribution to theory building)	Consideration in new model of how experience can be turned into constructive learning.

**CITATION 63: Henschen et al 2013**	
Country of origin	USA
Study design	Implementation and evaluation study of using the ‘Patient Centered Medical Home’ model as the basis for a longitudinal clerkship for medical students.
Context	Authors identified that medical students were not usually integrated into practices using the Patient Centered Medical Home model and so sought to do so in two community-based family medicine clinics, an academic internal medicine clinic and a paediatric clinic. Focus was on developing understanding of continuity of care, team-based care, care coordination and integration, quality and safety, and enhanced access to services.<br><br>56 medical student volunteers were recruited. ‘High risk’ patients were recruited to participate in each setting.
Intervention	Student teams were embedded into each practice and allocated a panel of patients. Peer teaching, supervision by qualified preceptors and grand rounds were used alongside patient care to support learning.<br><br>The patient centred medical home model is a model of practice where a lead clinician coordinates health professionals to work collaboratively to improve patient care. It has been most commonly used in the context of chronic illness.
Mechanisms triggered	Mechanisms of learning in practice were not studied.
Reported outcomes	Students attended 699 clinics, recruited 273 continuity patients, and participated in 9 grand rounds. Students reported increased confidence with the principles of the patient centred medical home model and increased valuing of continuity. Early clinical exposure and peer teaching were also highly valued.
Authors interpretation	Students were reported to become a cross between patient advocates and care providers.
Strengths and limitations	The study was uncontrolled and patient perspectives are not reported.
Our interpretation	The study provides an example of how integration of education and clinical care can be synergistic for all concerned albeit with respect to a specific intervention.
Use made of citation in synthesis (contribution to theory building)	Used to highlight importance of student participation in patient care not just for learning but to benefit all involved.

APPENDIX 3: MARKERS OF SUCCESS AND FAILURE INDENTIFIED IN THE SYNTHESIS

IN THE CONTEXT OF MULTIMORBIDITY IN PRIMARY CARE…	**HEALTH SERVICE DELIVERY**	**EXPERIENTIAL LEARNING** (Incorporating patient, professional, trainee and student learning from experience as review identified comparable processes in all groups with respect to sense-making and knowledge construction i.e. learning whether formally labelled as 'education' or not).	**CONCURRENCY**
**MARKERS OF SUCCCESS (EI MODEL STUDIES)**	• Reduced hospitalisations, improved care co-ordination, better health outcomes, doctors sharing tasks with other healthcare professionals to give more efficient care [24]<br>• Implementation of a 'Chronic Illness Care Management plan' (written plan developed between patient and physician of patients' goals, medication review, education and self-care, psychological and social assessment, community integration and social support and prevention activities) [112].	It was important for professionals to learn effective collaboration (personal relationships and connections) for high quality multidisciplinary-team working that included an active role for patients within 'their' team [24]	Learning and healthcare delivery were intertwined as changes in attitude were required for both patients and doctors in order for them to learn and work as a team [24]. The importance of professional ownership of change was identified as an essential mechanism to bring about practice developments [112].
**MARKERS OF SUCCCESS (EI STUDIES**	• Understanding each practice as a clinical microsystem in which provider knowledge and decision support are situated [80]<br>• Patient satisfaction with implementation of guided care model and improved knowledge amongst doctors of the clinical characteristics of their patients with chronic illness [87]<br>• Patient advocacy [63]	• Improved understanding of community / patient-centred practice with exposure [96, 98] and use of chronic care models [36, 63]<br>• Reciprocal learning between professionals from joint critical reflection, sharing knowledge and experience [80]<br>• Valuing continuity [63]<br>• Learning conceptualised as 'a social, shared process through which individuals incorporate new information in ways that lead them to change their mental models and adapt' within dynamic systems [80]	• Non-linearity and unpredictability expected and accounted for when implementing learning interventions [80]<br>• Integration of medical students into practice using the 'Patient Centered Medical Home' model produced increased patient support and learning about service delivery in chronic care [63]. Models like this focus on continuity of care, team based care, care coordination and integration, quality and safety and enhanced access. Integration of students allows them a role as patient advocates and contributors to meaningful care provision.<br>• A need identified of understanding more about creating the 'conditions necessary for collective learning with communities of clinicians who care for patients with multimorbidity and who develop new knowledge in practice' [126] as well as how best care is socially constructed or judged [80]
**MARKERS OF SUCCCESS (OTHER**	ENI<br>• Professional recognition of and attention to cognitive	ENI<br>• Doctors promote participation and direct	ENI<br>• When doctor provide students with authentic

**MODEL STUDIES)**	and emotional (e.g. sense of identity) aspects of illness, medication burden (risks of relative under/over management of particular conditions) and patient priorities (often dependent on current functional impact)<br>• Enablement of patients to make sense of relationships between illnesses in multimorbidity and prioritise competing relationships [19]<br>• Collaborative working with patients [81]<br>• 'Positive' strategies for coping pursued by patients: adherence, self-motivated change, negotiated empowerment [60]<br><u>TO</u><br>• Implementation of the 'CARE' approach: 'connecting, assessing, responding and empowering' [14], and other chronic care models / system level changes to using patient-centred approaches to produce productive interactions (recognising contrasting perspectives) between an informed and activated patient / significant others and proactive healthcare teams with visible leadership i.e. decisions made within the context of a person, appropriate prioritisation of goals and management and holistic approaches [14, 20, 31, 75]<br>• Developing understanding of multimorbid conditions on spectrum from concordant to discordant [31]<br>• 'Beacon practices' to develop professional education and patient care [67]<br>• Recognition that improving disease specific outcomes is not an automatic proxy for success [126]<br><u>PI</u><br>• Patients facilitated in a process of transformative learning following lived experiences of chronic illness, understanding that definitions of success can evolve with changing circumstances [10]<br>• Self-management by patients following negotiation of goals with professionals in shared decision making processes [55]<br>• Stabilising the condition to get patients back to living their lives, support through monitoring and review,	interaction between patients and students, and provide debriefing i.e. the doctor self-positioned as an expert resource offering challenge coupled with supported participation, senior assistance with cultural adjustment, graded responsibilities and active roles combined with scaffolding and conferring legitimacy [6, 40, 128]<br>• Development in students of a positive state of mind (confidence, motivation and sense of professional identity) as well as practical competence to become a doctor [6]<br>• Supportive learning environment with clear expectations of students; orientation to individual consultations, authentic role in patient care [6]<br>• Use of 'mindlines' as a mechanism to convey evidence to clinicians or bring about change [52]<br>• Development of communication skills for practitioners to enable them to hold unscripted conversations with patients [60]<br>• Patients learning to cope in different ways: social (maintaining a role, autonomy and having a can-do approach), emotional (when it is believed there is nothing to be done to change the situation, positive approach to life despite adversity) and practical (expectation of change, focused on control of disease and therapies) [81], developing self-efficacy [117]<br>• Learners employing both short loop (addressing immediate needs) and long loop (addressing complex and recurring problems which need longitudinal learning activities) learning as a result of patient interactions [114]<br>• Professional learning that is functional for care delivery [88]<br><u>TO</u><br>• Relational working and learning [14] Patient centred medical education,	roles in patient care, patients who choose to participate are able to benefit from student presence e.g. feeling valued, being given more time and gaining more information from questions asked by students (who also benefit from the converse as patients provide 'real life examples' had help students with communication skills [6]<br><u>PI</u><br>• Transformative learning and response shift models as mechanisms for professionals and patients to learn and work together to redefine success in the absence of cure [10]<br><u>LR</u><br>• Transformation as a process of learning about oneself and chronic illnesses in an iterative and continually changing manner [42]<br>• Educational alliances for learning, challenge as well as support, balance between patient and learner priorities, trust [138]<br><u>TO</u><br>• Learners holding responsibility for patient care in integrated models of learning and care delivery can organise doctors time effectively while facilitating learner-patient interaction [45]<br>• Modification of belief occurs through conversations as well as actions to provide learning and change; space needed for shared learning and collaborative working and unanticipated variation in intervention outcomes may be due to unexpected conversations that lead to new sense making and reorganisation of relationships and practice [70]<br>• Integration or skills and resources of all participants in chronic illness – patients, families and professionals through interventions that harness emergent learning and practice refined through

	timely intervention by appropriate service when things go wrong, providing choice and support towards the end of life: potential interventions to achieve this included seven day working and increased focus on individual patient need while seeking uniform quality across organisations, self-management, use of technology, partnership working, remote monitoring, shared care, reduced hospital admissions, advance care planning and access to information [99]<br>LR<br>• Importance placed on patient preferences, interpretation of evidence, prognostication, consideration of clinical feasibility and optimising therapies and care plans in order to provide individualised care that continually reviews risks versus benefits for patients [4, 75]<br>• Facilitating patients to embrace challenge and new ways of being [41]	learner-patient interaction; learning defined as producing optimal patient care authentic role in patient care, debriefing [15, 140]<br>• Positioning the doctor as an expert resource promoting participation with increased student-patient interaction to avoid patient and student passivity [15]<br>• Trusted relationships and communities of practice needed for on-going professional development [105]<br>• Responsibility hand in hand with learning [130,140]<br>• Legitimate role in practice, constructive feedback, active engagement to consider reapplying learning [140]<br>• Patients able to maintain a sense of humour, social support, keep active and take responsibility [126]<br>LR<br>• Understanding how and why people change when living with chronic illness, identification of needs, support for constructive adaption [42]<br>• Learning successful management of multimorbidity: weighing up prognosis, multi-factorial problems and symptoms, feasibility or management decisions and implementation, consideration of interactions between treatments and interventions for one condition and coexisting conditions [4]<br>PI<br>• Space to learn and grow professional identity [56]<br>• Integrated learning and practice [56]<br>• Self-management, use of technology to support rehabilitation, partnership working, remote monitoring, shared care, reducing hospital admissions, advanced care planning and access to information [99]	interactions with patients, e.g. making trust and compassion a strategic requirement, co-creation of care plans, recording of learning process [126]<br>• Trust and relationships are crucial and can be developed through action learning frameworks [126, 127] with patients and families central to the care process: communities of professionals need to learn to engage in the co-creation of care plans.<br>• Organisational culture that favours a sense of community, trust, openness to experimentation and discovery [127]<br>• Need to understand more about creating the 'conditions necessary for collective learning with communities of clinicians who care for patients with multimorbidity and who develop new knowledge in practice' [126].
**MARKERS OF**	• Physicians reluctant to consider non biomedical	• Lack of team work, face to face interaction or	*No explicit markers identifiable – implicitly the opposite*

**FAILURE (EI MODEL STUDIES)**	factors in chronic illness management (suggesting this was a nursing task) or believing patients incapable of collaborative working.[112]	understanding of roles and capabilities [24]<br>• Physicians skilled in crisis management of disease rather than long term care [112]	*of markers of success*
**MARKERS OF FAILURE (EI STUDIES)**	*No explicit markers identifiable – implicitly the opposite of markers of success*	*No explicit markers identifiable – implicitly the opposite of markers of success*	*No explicit markers identifiable – implicitly the opposite of markers of success*
**MARKERS OF FAILURE (OTHER MODEL STUDIES)**	<u>ENI</u><br>• Not accounting for patient perspectives in management [19]<br>• Lack of time preventing holistic approach (Martin 2002)<br>• Focus on suppressing disruption due to new diagnoses or changes in conditions, use of medication as a negotiating tool [89]<br>• 'Negative' strategies for coping pursued by patients: e.g. passivity/fatalism, unguided searching, disengagement [60, 89]<br><u>LR</u><br>• Lack of current management approach for the multiple problems particular to each individual with multimorbidity [4]<br><u>TO</u><br>• Lack of negotiation between patients and professionals [14]<br>• Lack of matching patients' needs to best therapeutic practices ('satisficing' 75] instead only focusing on single dimension of care (leading to unmet patient/professional learning needs, fragmentation, avoidable inpatient admissions or preventable complications, or receiving interventions for which there is little evidence of benefit and high risk of adverse health events [20, 33, 14, 126]<br>• Failure to reconcile patient focus (commonly on severity of symptoms, functional demands, informational and management needs) and clinical focus (commonly on identification of index condition, complications, risks, system constraints, medications, lab values and provider preferences) [31]<br>• Episodic approach to management rather than approaches integrated into daily routines for	<u>ENI</u><br>• Passive roles for patients and students [6]<br>• Student anxiety about causing offence/being perceived as unprofessional if they identified too strongly with patients [6]<br>• Sophisticated workarounds in response to dilemmas, inconsistences and unpredictable events – the experience of professionals shapes their practice more than externally imposed reform [22]<br><u>PI</u><br>• Lack of exposure during training [56] although this may be changing with the changing nature of healthcare<br>• Competency based curricula focused on outcomes with lack of recognition of complex interplay between actors, context, process and content [56]	<u>ENI</u><br>• Misapplication of patient safety agendas leading to risk avoidance rather than management strategies: detrimental to learning in the short term and patient care in the longer term [39,40]<br>• Understanding facets of control: the longer an illness lasts the more control over its outcomes individuals perceive – treatment control predicts personal control and emotional response but personal control can also predict treatment control and both are predicted by self-efficacy [117]<br><u>LR</u><br>• Lack of readiness / willingness to change [42]

	patients [31]<br>PI<br>• Patient not in a state of 'readiness for change' [10]<br>• Not working in partnership with patients [55]		
**OTHER ISSUES IN ENI STUDIES RELEVENT TO RESEARCH QUESTIONS**	• 'an endless struggle' for patients and professionals [101]<br>• Poor patient experience of care processes especially beyond treatment of medical conditions [33, 51]<br>• Individualisation, applying an integrated approach, medical considerations placed in perspective of shared decision-making and responsibility are necessities of daily practice for GPs [84] but there is a lack of mechanisms for unplanned and opportunistic care, and existing structures can militate against a holistic service for patients [19]<br>• Exercise, mindfulness and social prescribing needed as part of holistic care [92]<br>• Gaps in support and fragmentation of care and need for continuous remodelling of longitudinal care (13, 11 51]<br>• Concerns about lack of outcome accountability and/or functional communication systems, recognition of complexity of patient self-management [1, 13, 33]<br>• Professional perceptions of tensions between effort of delivery of activities and perceived potential for patient benefit [13, 19]<br>• Time lacking for complexity management [13, 19, 95]<br>• Quality and coordination of patient experiences do not have a straightforward correlation with clinical markers of disease although having a regular doctor works best for most people. Those with chronic lung or mental health problems appear to be at risk of the least well-coordinated or quality care [22]<br>• Tensions between GP-led, shared or specialist-led care [33]<br>• Need for active case finding by professionals [1]<br>• Challenges in joint goal setting: additional patient support does not necessarily increase this [3]	• Disrupted identities for patients with imperative to learn to balance between 'holding on' and 'letting go' as a means of expressing or withdrawing agency and control: seeking to restructure oneself, differentiating oneself from illness, lowering self-expectations and new meaning-making through narrative revisions to create a reconciled identity and accommodate shifts in time and person [8]<br>• Patients make trade-offs between morbidity and mortality (QOL v life expectancy) that may differ to those of professionals. One study found that patients with chronic illness were willing to give up 7.6 months of life and take a 5.1% increased chance of death in exchange for better than average health [120]<br>• Patients want to gain important self-management skills: correct use of medication, improved sleep, managing pain, reducing stress [51]<br>• Learning needed to manage competing priorities between co-existing diseases and drug-drug interactions [11]<br>• Learning needed to trade one risk with another according to current priorities e.g. continuity with convenience in consultations [13]<br>• Patient understanding of what a medical student or trainee is can be very variable [74]<br>• Patients with multimorbidity sometimes conceptualised as 'problem patients' by professionals and learners [11]<br>• Interpersonal relationships, professional development, personality, and teaching quality are important [97]	• Importance of narratives and story-telling for making sense [1,2]<br>• Variability in communication within and without consultations [9]<br>• Multimorbidity involves 'unique constellations of problems, shifting priorities and multi-dimensional decision-making' [13]<br>• Importance of respect [33]<br>• Undue focus on changing attitudes of physicians when they do not feel they have competency or control over organisation of services [34]<br>• Darer [34] surveyed doctors perceptions of adequate training against 10 competencies applicable to people with one or more chronic conditions, and not specific to a single disease (including geriatric syndromes, chronic pain, nutrition, developmental milestones, end-of-life care, psychosocial issues,<br>• patient education, assessment of caregiver needs, coordination of services, and interdisciplinary teamwork). Few doctors thought their training was adequate although this was higher in primary care than hospital trained specialists.<br>• Learners dislike a focus on managing disease, protocol driven behaviours, haphazard changes and emphasis on presenting problem alone in training interactions [39, 40].<br>• Patients can have problems in interacting with healthcare providers with respect to management of symptoms to maximise functionality [100]<br>• Patient perception of doctor empathy may affect enablement [92]

	• Narrow view of patient empowerment as managing medication regimes and/or following the direction of health care providers amongst some professionals [8]<br>• Need for professionals to understand that patients make contact with providers when self-management options (e.g. taking control of organising care) are exhausted [13, 33]<br>• Patient use of index condition models differs to professionals use of the same as patients tend to focus on whatever condition is most problematic at the time [33]<br>• Patients keen to participate in prioritisation according to own decision-making values [50]<br>• GPs say shared-decision making should come with shared responsibilities but many still feel overly responsible [84,135]<br>• Difficult for GPs to convert quality of life judgements (based on the meaning of illness for a patient) into appropriate medical practice [84]<br>• GPs: able to distinguish between symptomatic and preventative medication but find the latter harder to deprescribe; prefer to avoid potentially confrontational discussions about life expectancy and/or feel under pressure to conform to guidelines [118]<br>– Guidelines can paradoxically increase adverse drug events in elderly patients while expert driven recommendations do not necessarily impact on quality of life [118]<br>– lack of distinction in many guidelines between multiple chronic conditions which are symptomatic and multiple chronic risk factors [123]<br>• Need to recognise high prevalence of mental health disorders in multimorbidity and professional reluctance to diagnose depression in the context of chronic illness [11, 92, 19]<br>• Much of the NICE guidance on patient experience is based on expert consensus rather than research [103]	• Patient belief they have an important role to play [100] but need active engagement of doctors [100] to work collaboratively and take responsibility for the proactive management of chronic illness<br>• Trainee doctors unable to answer questions about their patients with chronic disease such as what their goals were in caring for them, what were the major obstacles in achieving these goals, what strategies could overcome the obstacles? or identify what patients wanted from them as overly focused on biomedical models [12]<br>• Need for all to develop understanding that definitions of success can change in unpredictable ways as the circumstances change; redefining of health can occur [13]<br>• Ambivalence towards change by doctors [19]<br>• Conferring of legitimacy, providing challenge with support and aiding cultural adjustment essential for learning [30]<br>• Role modelling is most effective when role models have clarity of ideas about what they are offering and why [32]. Practice is socially constructed and so learned behaviour and attitudes, and innovation in how people actually work is influenced by role models (N.B possible for outcome to be 'good' learning from poor role models [90])<br>• Lack of training for chronic illness [34, 141] is translating into increased exposure but unclear how much is enough [37, 65]<br>• Fostering of can-do attitude rather blaming of systems failures needed among professionals [68]<br>• Challenges for professionals can stem from internal learning needs (lack of awareness of guidelines, disagreement with recommendations, uncertainty regarding relevance / applicability / modifications, lack of motivation due to low expectation of	• Recogntition, relevance, emotion, immediacy, authenticity are important for learning [106]<br>• Relational continuity is often assumed to provide better understanding of each other and so the desire for relational continuity higher if a problem indicates serious illness [115]<br>• Breakdowns [122] – defined as common themes in the success or failure of human activities whether related to learning, care delivery or other endeavours can lead to various problems: personal (roles and responsibilities, clinical uncertainty, avoidance of discussions, sense of disempowerment and despair amongst professionals), or contextual and health system factors (lack of time, fragmentation of care, communication) but equally form the basis of new learning [123]<br>• GPs become dispensers of capital through authenticating the sick role [132] which can protect patients from the prevalent social view that those who are ill should pursue recovery to function effectively in social roles – need to understand the resources people have to live and act with [132]<br>• 'Illness puts one at risk of failure in everyday life' [132]<br>• Mutual respect, plus space to learn and grow professional identity are important for learners [134]<br>• Patients viewed by medical students as occupying a zone of transition between society and healthcare when interacting in general practice [134]

		clinical impact) or external factors (structures do not permit/facilitate innovation) [83]<br>• Community based learning best for gaining understanding about psychosocial issues, patient autonomy and communication skills [104]<br>• Collaborative structures and good interpersonal networks more effective for patient care than hierarchy [119]	
**OTHER ISSUES IN OTHER LITERATURE (PI, LR, TO) – only included if novel to items identified in rows above**	• Professionals need the provision of a mandate to focus on patients' lived experiences of symptoms [47]<br>• Working practices usually conservative and resistant to change [21]<br>• The industrialisation of medicine is paradoxical to the aims of shared decision making and patient centred care [113]<br>• Primary care is an example of a complex adaptive system where a collections of individual agents with freedom to act in ways that are not always totally predictable and whose actions are interconnected so that one agents actions changes the context for other agents<br>• Information continuity is defined as having all the necessary information about the patient at the point of care. It differs from, and might be argued is less than, management continuity which requires coordinating actions with other providers to deliver services in a complementary and timely manner along a recommended care pathway and still more so relational continuity which implies development of a trusting partnership [59].<br>• Need for comprehensivists not generalists and specialists [131]<br>• Some patients want their values to guide decision-making but not to necessarily take an active role in the decision-making process itself [50]<br>• Very limited evidence for longer appointments, integration of different clinical pathways with assistance with prioritisation, different modes of service provision such as guided health, telehealth	• Medical socialisation of trainees occurs alongside learning in practice [61]<br>• Desirable characteristics and qualities of trainers: Good at giving feedback, dares to give feedback, critical of trainees and learning processes, good at communicating with the trainee, respect for the trainee and able to inspire the trainee [17]<br>• Transition from hospital into general practice can be associated with isolation and loneliness [30]<br>• Patients have low expectations of being symptom free [33]<br>• Historical weighting of medical training towards acute settings [7]<br>• Persistence of disease-based approaches despite recognised insufficiency [23]<br>• Patients have their own thresholds for medication adherence and often take less than the prescribed medication [94]<br>• Lack of) shared information and decision making: side effects, fear of harm/dependence, complex instructions, sub-optimal communications, suspicions about motivations for prescribing and cost [94]<br>• Doctors educated to prescribe according to guidelines that are not designed for the patients they actually provide care for [139]. Lack of advice regarding prioritisation of recommendations according to risk-benefit analysis, cross-referencing, examples of common multimorbidities and treating older	• Choice is relational, dependent on nuanced preferences, value judgements, fluctuates in time as well as between people<br>• Time an underrecognised but crucial resource essential for the implementation of innovative tools to coordinate care [71]<br>• GPs – believe deprescribing will conflict with patient perceptions of benefit, feel information is lacking to assess risk-benefit balance, concerned this would be perceived as giving up [118]<br>• Societal expectation that we can only be killed rather than die naturally and therefore death is a result of failure to find a medical cure.<br>• Doctors and patients were positively disposed to students being present with doctors more concerned about confidentiality than patients, patients with experience of students were less concerned about seeing doctors alone [109]

	etc. although all intuitively helpful [71]<br>• Frailty, loneliness and social isolation all occur as part of morbidity with age [16]<br>• Patients often experience emotional oscillation between anxiety and strength [81]<br>• Team-working in practice should lead to 'a patient dependent team not a team dependent patient' [29]<br>• EBM alone cannot provide clear answers for these patients [139]<br>• Non-adherence to medication linked to multiple and uncoordinated prescriptions is a costly and widespread problem [16]<br>• Recognition of need for care co-ordination, explicitly overall responsibility, principles of palliative care, mental health, consideration of vulnerable groups and change from reductionist models [38]<br>• One study which defined quality of care as being offered recommended services and achieving biomedical markers (i.e. the provision of recommendations by disease) found that quality of care increases in multimorbidity [64]. This might be viewed by others as a problematic definition of quality, perhaps necessary but not sufficient. Others have suggested that paradoxically greater contact with healthcare services may lead to possibly poor quality of care in holistic terms [11]	people [66]<br>• Not every patient wants to participate in shared decision making [79] but this is perceived to be ethically correct. Some concerns have been raised about the risks of shared decision making leading to demands for more resources [79]<br>• Assumption that competency based approaches to education will provide doctors equal to societal expectations [61]<br>• Harnessing of new technology is needed to make guidelines more flexible and overcome differences between how guidelines are created and how everyday practice functions [57]<br>• Guidelines should contain provision of a rationale for prioritising recommendations and emphasise requirement for judgement and discussion with patients about risk-benefit balance [43]<br>• Guidelines can influence medical training adversely [57]: There is a changing role of guidelines as artefacts influencing medical practice from 'well intentioned emphasis on the evidence base to the proliferation of guidelines to indiscriminate incentives or performance related pay to the slow transmorphing into tablets of law' suggesting that doctors no longer feel able to make professional judgements when necessary to tailor treatment to the needs, aspirations, values and context of individual patients.<br>• Trainees who refuse to consider deviation from guidelines leading to over diagnosis and over treatment [57]	

Endnotes:

[i] Dewey J. Experience and education. 1938, New York, NY: Collier Books

[ii] Mezirow JD. Learning as transformation: critical perspectives on a theory in progress. 2000, 1st edn. San Francisco: Jossey-Bass

[iii] *Ibid, p8.*

[iv] Lave J and Wenger E. Situated Learning: Legitimate Peripheral Participation. 1991, Cambridge: Cambridge University Press

[v] Engeström Y, Expansive learning at work: toward an activity theoretical reconceptualization. Journal of Education and Work, 2001, 14:133-156. Engeström Y. Toward the encapsulation of school learning. 1991 In:DANIELS H, ed. An introduction to Vygotsky. 1991, 2nd edn. London: Routledge, pp. 153-172

[vi] Eraut M. Informal learning in the workplace. Studies in Continuing Medical Education, 2004, 26:247-273

[vii] Wong G, Westhorp G, Pawson R, Greenhalgh T. Realist synthesis: RAMESES Training Meterials. 2013. Available at http://www.ramesesproject.org/media/Realist_reviews_training_materials.pdf [Accessed 20.10.14]

[viii] Yardley S, Cottrell E, Protheroe J. Understanding success and failure in multimorbidity: protocol for using realist synthesis to identify how social learning and workplace practices can be optimised. Systematic Reviews 2013, 2:87

[ix] Pawson R: The science of evaluation: a realist manifesto. 2013, London: Sage

[x] Billet S. Workplace pedagogic practices: participation and learning. Australian Vocational Education Review 2002, 9:28-38
[xi] Kozulin A, Chaiklin S, Karpov Y, Egan K, Gajdamaschko N, Lidz Cs, Gindis B, Mahn H, Bodrova E, Leong Dj, Zuckerman G, Haenen J, Schrijnemakers H, Stufkens J, Giest H, Lompscher J, Miller Sm, Dipardo A, Potter C, Lantolf Jp, Portes Pr, Vadeboncoeur Ja, Panofsky Cp And Ageyev Vs .Vygotsky's educational theory in cultural context. 2003, Cambridge: Cambridge University Press

[xii] Yardley S, Teunissen PW, Dornan T. Experiential learning: AMEE Guide No. 63. Medical Teacher, 2012: 34:e102-15

[xiii] Crotty M. The foundations of social research: meaning and perspective in the research process. 1998, Sage : London

[xiv] Sinnott C, Mc Hugh S, Browne J, Bradley C. GPs' perspectives on the management of patients with multimorbidity: systematic review and synthesis of qualitative research. BMJ Open 2013, 3:e003610-2013-003610

[xv] Kuluski K, Gill A, Naganathan G, Upshur R, Jaakkimainen RL, Wodchis WP. A qualitative descriptive study on the alignment of care goals between older persons with mutli-morbidities, their family physicians and informal caregivers. BMC Family Practice 2013, 14:133

[xvi] Dornan T, Hadfield J, Brown M, Boshuizen H, Scherpbier A. How can medical students learn in a self-directed way in the clinical environment? Design-based research. Med Educ 2005, 39: 356-64

[xvii] Littlewood S, Ypinazar V, Margolis SA, Scherpbier A, Spencer J, Dornan T. Early practical experience and the social responsiveness of clinical education: systematic review. British Medical Journal 2005, 331: 387-391. Yardley, S, Littlewood S, Margolis SA, Scherpbier A, Spencer J, Ypinazar V, Dornan T. What has changed in the evidence for early experience? Update of a BEME systematic review. Medical Teacher 2010, 32:740-746

[xviii] Hopayian K, Howe A, Dagley V. A survey of UK medical schools' arrangements for early patient contact. Medical Teacher 2007, 29: 806-813

[xix] Bell K, Boshuizen HPA, Scherpbier A, Dornan T. When only the real thing will do: junior medical students learning from real patients. Med Educ 2009, 43: 1036-43

[xx] Van der Zwet J, Hanssen VGA, Zwietering PJ, Muijtjens AMM, Van der Vleuten CPM, Metsemakers JFM, Scherpbier AJJA. Workplace learning in general practice: supervision, patient mix and interdependence emerge from the black box once again. Medical Teacher, 2010, 32:e294-299

[xxi] Steven K, Wenger E, Boshuizen H, Scherpbier A, Dornan T. How clerkship students learn from real patients in practice settings. Acad Med 2014, 89:469-476

[xxii] Van der Zwet J, Dornan T, Teunissen PW, de Jonge LPJWM, Scherpbier AJJA. Making sense of how physician preceptors interact with medical students: discourses of dialogue, good medical practice and relationship trajectories. Adv in Health Sci Educ 2014, 19:85-98

[xxiii] Craig P, Dieppe P, Macintyre S, Michie S, Nazareth I, Petticrew M. Developing and evaluating complex interventions: the new medical research council guidance. BMJ 2008, 337:a1655
