# Supplementary material for: Modelling successful primary care for multimorbidity: a realist synthesis of successes and failures in concurrent learning and healthcare delivery
Source: BMC Fam Pract. 2015 Feb 25;16:23. doi: 10.1186/s12875-015-0234-9 (PMC4343192; doi:10.1186/s12875-015-0234-9)
Supplement: Additional file 2: — Search strategy. [file 12875_2015_234_MOESM2_ESM.docx]

# Additional file 2. Medline search strategy (via NHS Evidence)

| **1 AND 2 AND (3 OR 4)** |  |
| --- | --- |

1. Multimorbidity

| **No** | **Search term** |
| --- | --- |
| **1** | exp COMORBIDITY/ |
| **2** | comorbid*.ti,ab |
| **3** | co-morbid*.ti,ab |
| **4** | multimorbid*.ti,ab |
| **5** | multi-morbid*.ti,ab |
| **6** | exp CHRONIC DISEASE |
| **7** | (chronic adj2 disease*) .ti,ab |
| **8** | exp DISEASE PROGRESSION |
| **9** | (disease adj2progress*). Ti, ab |
| **10** | 1 OR 2 OR 3 OR 4 OR 5 OR 6 OR 7 OR 8 OR 9 |

1. Primary Care

| **No.** | **Search Term** |
| --- | --- |
| **1** | exp FAMILY PRACTICE/ |
| **2** | exp PHYSICIANS, FAMILY/ |
| **3** | exp PRIMARY HEALTH CARE/ |
| **4** | exp COMMUNITY HEALTH SERVICES/ |
| **5** | "general practi*".ti,ab |
| **6** | GP.ti,ab |
| **7** | (primary adj2 care).ti,ab |
| **8** | (family adj2 physician*).ti,ab |
| **9** | (family adj2 practi*).ti,ab |
| **10** | (family adj2 doctor*).ti,ab |
| **11** | ((community adj2 (service* OR care))).ti,ab |
| **12** | (general adj2 physician*).ti,ab |
| **13** | 1 OR 2 OR 3 OR 4 OR 5 OR 6 OR 7 OR 8 OR 9 OR 10 OR 11 OR 12 |

1. Education

| **No** | **Search term** |
| --- | --- |
| **1** | learn*.ti,ab |
| **2** | exp EDUCATION, MEDICAL/ |
| **3** | Educat*.ti,ab |
| **4** | 1 OR 2 OR 3 |

1. Workplace experience (note: ‘training’ is incorporated into the term ‘education’)

| **No.** | **Search term** |
| --- | --- |
| **1** | exp WORKPLACE/ |
| **2** | workplac*.ti,ab |
| **3** | (experien*adj2learn*).ti,ab |
| **4** | exp NARRATION/ |
| **5** | narrat*.ti,ab |
| **6** | exp ATTITUDE OF HEALTH PERSONNEL/ |
| **7** | attitude*.ti,ab |
| **8** | 1 OR 2 OR 3 OR 4 OR 5 OR 6 OR 7 |
| **9** | exp STUDENTS MEDICAL/ |
| **10** | Medical student*.ti,ab |
| **11** | exp EDUCATION MEDICAL GRADUATE |
| **12** | Medical graduate*.ti,ab |
| **13** | 9 OR 10 OR 11 OR 12 |
| **14** | 8 AND 13 |
