# Supplementary material for: Modelling successful primary care for multimorbidity: a realist synthesis of successes and failures in concurrent learning and healthcare delivery
Source: BMC Fam Pract. 2015 Feb 25;16:23. doi: 10.1186/s12875-015-0234-9 (PMC4343192; doi:10.1186/s12875-015-0234-9)
Supplement: Additional file 3: — Citation list. [file 12875_2015_234_MOESM3_ESM.docx]

**Additional files: List of all citations included in the synthesis**

1. Ahmed S, Gogovor A, Kosseim M, Poissant L, Riopelle R, Simmonds M, Krelenbaum M, Montague T : Advancing the chronic care road map: a contemporary overview. Health Quart 2010, 13:72-9.
2. Alderson TS, Bateman H, Alderson TSJ: Doctors telling stories: the place of anecdote in GP registrar training. Med Teach 2002, 24:654–7.
3. Alexander JA, Hearld LR, Mittler JN, Harvey J: Patient-physician role relationships and patient activation among individuals with chronic illness. Health Serv Res 2012, 47:1201-1223.
4. American Geriatrics Society Expert Panel on the Care of Older Adults with Multimorbidity: Guiding principles for the care of older adults with multimorbidity: An approach for clinicians. J Am Geriatr Soc 2012, 60:E1-25.
5. Anonymous: New approach to behavior change geared toward individual practitioners. Dis Manag Advis 2002, 8:93-5, 81.
6. Ashley P, Rhodes N, Sari-Kouzel H, Mukherjee A, Dornan T: ‘They’ve all got to learn’. Medical students’ learning from patients in ambulatory (outpatient and general practice) consultations. Med Teach 2009, 31:e24-31.
7. Assal JP: Revisiting the approach to treatment of long-term illness: from the acute to the chronic state. A need for educational and managerial skills for long-term follow-up. Pat Educ Counsel 1999, 37:99-111.
8. Aujoulat I, Marcolongo R, Bonadiman L, Deccache A: Reconsidering patient empowerment in chronic illness: a critique of models of self-efficacy and bodily control. Soc Sci Med 2008, 66:1228-39.
9. Bain DJ: Doctor-patient communication in general practice consultations. Med Educ 1976, 10:125-31.
10. Barclay-Goddard R, King J, Dubouloz CJ, Schwartz CE, Response S: Building on transformative learning and response shift theory to investigate health-related quality of life changes over time in individuals with chronic health conditions and disability. Arch Phys Med Rehabil 2012, 93:214-20.
11. Barnett K, Mercer SW, Norbury M, Watt G, Wyke S, Guthrie B: Epidemiology of multimorbidity and implications for health care, research, and medical education: a cross-sectional study. The Lancet 2012, 380:37-43.
12. Baughan DM, Revicki D, Nieman LZ: Management of problem patients with multiple chronic diseases. J Fam Pract 1983, 17: 233-239.
13. Bayliss A, Edwards E, Steiner F, Main S: Processes of care desired by elderly patients with multimorbidities. Fam Pract 2008, 25:287-293.
14. Bikker AP, Mercer SW, Cotton P. Connecting, Assessing, Responding, Empowering (CARE): A universal approach to person-centred, empathic healthcare encounters. Ed Prim Care 2012, 23:454-457.
15. Bleakley A, Bligh J: Students learning from patients: let’s get real in medical education. Adv Health Sci Educ Theory Pract 2008, 13:1573-1677.
16. Boeckxstaens P, De Graaf P: Primary care and care for older persons: position paper of the European Forum for Primary Care. Qual Prim Care 2011, 19:369-389.
17. Boendermaker PM, Conradi MH, Schuling J, Meyboom-de Jong B, Zwierstra RP, Metz JC: Core characteristics of the competent general practice trainer: a Delphi study. Adv Health Sci Educ Theory Pract 2003, 8:111–6.
18. Bower PF, Harkness EF, Macdonald WF, Coventry PF, Bundy CF, Moss-Morris R: Illness representations in patients with multimorbid long-term conditions: Qualitative study. Psychol Health 2012, 27:1121-26.
19. Bower P, Macdonald W, Harkness E, Gask L, Kendrick T, Valderas JM, Dickens C, Blakeman T, Sibbald B: Multimorbidity, service organization and clinical decision making in primary care: A qualitative study. Fam Pract 2011, 28:579-587.
20. Boyd CM, Fontin M: Future of multimorbidity research: how should understanding of multimorbidity inform health system design? Pub Health Rev, 2010 32:451-474.
21. Brown JS, Duguid P: Organizational learning and communities-of practice: toward a unified view of working, learning, and innovation. Org Science 1991, 1:40-57.
22. Burgers JS, Voerman GE, Grol R, Faber MJ, Schneider EC: Quality and Coordination of Care for Patients With Multiple Conditions: Results From an International Survey of Patient Experience. Eval Health Prof 2010, 33:343-364.
23. Cassell EJ: Teaching the fundamentals of primary care: a point of view. Milbank Q 1995, 73):373-405.
24. Chan BC, Perkins D, Wan Q, Zwar N, Daniel C, Crookes P, Harris MF: Finding common ground? Evaluating an intervention to improve teamwork among primary health-care professionals. Int J Qual Health Care 2010, 22:519-24.
25. Cherubini A, Corsonello A, Lattanzio F: Underprescription of beneficial medicines in older people: causes, consequences and prevention. Drugs Aging 2012, 29:463-475.
26. Clark NM, Gong M: Management of chronic disease by practitioners and patients: are we teaching the wrong things? BMJ 2000, 320:572-5.
27. Clark NM, Nothwehr F, Gong M, Evans D, Maiman LA, Hurwitz ME, Roloff D, Mellins RB: Physician-patient partnership in managing chronic illness. Academic Medicine 1995, 70:957-9.
28. Colditz GA: Medical education meeting community needs. Med Educ 1983, 17:291-5.
29. Coles C: Educating the health care team. Pat Educ Counsel 1995, 26:239-44.
30. Cornford C, Carrington B: A qualitative study of the experiences of training in general practice: a community of practice? J Educ Teach 2006, 32: 269–82.
31. Corser WD: Increasing Primary Care Comorbidity: A Conceptual Research and Practice Framework. Res Theory Nurs Pract 2011, 25:238-251.
32. Cote L, Leclere H. How clinical teachers perceive the doctor–patient relationship and themselves as role models. Acad Med 2000, 75 :1117–24.
33. Cowie L, Morgan M, White P, Gulliford M: Experience of continuity of care of patients with multiple long-term conditions in England. J Health Serv Res Policy 2009, 14:82-87.
34. Darer JD, Hwang W, Pham HH, Bass EB, Anderson G: More training needed in chronic care: a survey of US physicians. Acad Med 2004, 79:541-8.
35. Dawes M. Co-morbidity: we need a guideline for each patient not a guideline for each disease. Fam Pract 2010, 27:1-2.
36. Dent MM, Mathis MW, Outland M, Thomas M, Industrious D: Chronic disease management: teaching medical students to incorporate community. Fam Med 2010, 42:736-40.
37. de Jong J, Visser MR, Mohrs J, Wieringa-de Waard M: Opening the black box: the patient mix of GP trainees. Brit J Gen Pract 2011, 61:e650-7.
38. Department of Health: Improving quality of life for people with long term conditions. Department of Heath, 2013. London. Available at: [<https://www.gov.uk/government/policies/improving-quality-of-life-for-people-with-long-term-conditions>] (last accessed 31.07.14)
39. Dornan T, Scherpbier A, Boshuizen H: Supporting medical students’ workplace learning: experience-based learning (ExBL). Clin Teach 2009, 6: 167-71.
40. Dornan T, Boshuizen H, King N, Scherpbier A: Experience-based learning: a model linking the processes and outcomes of medical students’ workplace learning. Med Educ 2007, 41: 84-91.
41. Dubouloz CJ, King J, Paterson B, Ashe B, Chevrier J, Moldoveanu M: A model of the process of transformation in primary care for people living with chronic illnesses. Chron Illness 2010a, 6:282-93.
42. Dubouloz, CJ, King J, Ashe B, Paterson B, Chevrier J, Moldoveanu M: The process of transformation in rehabilitation: what does it look like? Int J Ther Rehabil 2010b, 17:604-15.
43. Durso S: Using clinical guidelines designed for older adults with diabetes mellitus and complex health status. JAMA 2006, 295:1935–1940.
44. Ekdahl AW, Hellstrom I, Andersson L, Friedrichsen M: Too complex and time-consuming to fit in! Physicians' experiences of elderly patients and their participation in medical decision making: a grounded theory study. BMJ Open 2012, 2:e001063.
45. Ferenchick G, Simpson D, Blackman J, DaRosa D, Dunnington G: Strategies for efficient and effective teaching in the ambulatory care setting. Acad Med 1997, 72:277–80.
46. Fernald DH, Staudenmaier CJ, Main DS, O’Brien-Gonzales A, Barley GE: Student perspectives on primary care preceptorships: enhancing the medical student preceptorship learning environment. Teach Learn Medicine 2001, 13:13-20.
47. Fortin M, Hudon C, Bayliss EA, Soubhi H, Lapointe L: Caring for body and soul: The importance of recognizing and managing psychological distress in persons with multimorbidity. Int J Psych Med 2007, 37:1-9.
48. Fortin M, Soubhi H, Hudon C, Bayliss EA, van den Akker M: Multimorbidity's many challenges. BMJ 2007, 334:1016-1017.
49. Fortin M, Bravo G, Hudon C, Lapointe L, Dubois MF, Almirall J: Psychological distress and multimorbidity in primary care. Ann Fam Med 2006, 4:417-422.
50. Fried T, McGraw S, Agostini J, Tinetti M: Views of older persons with multiple morbidities on competing outcomes and clinical decision making. J Am Geriatr Soc 2008, 56: 1839-44.
51. Frueh BC, Larme AC, Noel PH, Pugh JA: Collaborative care needs and preferences of primary care patients with multimorbidity. Health Expect 2005, 8:54-63.
52. Gabbay J, le May A: Evidence based guidelines or collectively constructed “mindlines?” Ethnographic study of knowledge management in primary care. BMJ 2004, 329:1013.
53. Gaver A, Borkan JM, Weingarten MA: Illness in context and families as teachers: a year-long project for medical students. Acad Med 2005, 80:448-51.
54. Geyman JP, Bliss E. What does family practice need to do next? A cross-generational view. Fam Med 2001, 33:259-67.
55. Glasgow RE, Davis CL, Funnell MM, Beck A: Implementing practical interventions to support chronic illness self-management. Joint Commission Journal on Quality & Safety 2003, 29:563-74.
56. Glasgow NJ, Wells R, Butler J, Gear A: The effectiveness of competency-based education in equipping primary health care workers to manage chronic disease in Australian general practice settings. Med J Aust 2008, 188:S92-6.
57. Guthrie B, Payne K, Alderson P, McMurdo MET, Mercer SW: Adapting clinical guidelines to take account of multimorbidity. BMJ 2012, 345:e6341.
58. Haas WH, Crandall LA, Bain DJ: Characteristics of family practitioners with large geriatric practices. J Am Geriatr Soc 1980, 28:289-94.
59. Haggerty JL: Ordering the chaos for patients with multimorbidity. BMJ 2012, 7876:e5915.
60. Haidet P, Kroll TL, Sharf BF: The complexity of patient participation: lessons learned from patients' illness narratives. Pat Educ & Counsel 2006, 62:323-9.
61. Heath I, Rubinstein A, Stange KC, van Driel ML: Quality in primary health care: a multidimensional approach to complexity. Br Med J 2009, 338:b1242.
62. Heath I. In praise of young doctors. BMJ 2012, 345:e4549-e4550.
63. Henschen BL, Garcia P, Jacobson B, Ryan ER, Woods DM, Wayne DB, Evans DB: The patient centred medical home as curricular model: perceived impact of the “Education-Centred Medical Home”. J Gen Int Med 2013, 28:1105-9.
64. Higashi T, Wenger NS, Adams JL, Fung C, Roland M, McGlynn EA, Reeves D, Asch SM, Kerr EA, Shekelle PG : Relationship between number of medical conditions and quality of care. N Engl J Med 2007, 356:2496-504.
65. Hobbs J, Speers S, Herbert J, Nixon G, Poteet L, Hatch P: Clinical resources to teach components of a new Family Medicine Clerkship Curriculum. Fam Med 2011, 43:566-73.
66. Hughes LD, McMurdo, ME, Guthrie, B: Guidelines for people not for diseases: the challenges of applying UK clinical guidelines to people with multimorbidity. Age Aging 2013, 42:62-69.
67. Jackson CL, Askew DA, Nicholson C, Brooks PM: The primary care amplification model: taking the best of primary care forward. BMC Health Serv Res 2008, 8:268.
68. Johnson JK, Woods DM, Stevens DP, Bowen JL, Provost LP, Sixta CS, Wagner EH: Joy and challenges in improving chronic illness care: capturing daily experiences of academic primary care teams. J Gen Int Med 2010, 25:S581-5.
69. Jones DS: Needed: A coherent architecture for 21st-century clinical practice and medical education. Alt Ther Health Med 2010, 16:64-7.
70. Jordan ME, Lanham HJ, Crabtree BF, Nutting PA, Miller WL, Stange , Kurt C, McDaniel , Reuben R Jr: The role of conversation in health care interventions: Enabling sensemaking and learning. Implement Sci 2009, 4:1-13.
71. Kadam U: Redesigning the general practice consultation to improve care for patients with multimorbidity. BMJ 2012, 345:e6202.
72. Kamerow D: How can we treat multiple chronic conditions? BMJ 2012, 29:e1487.
73. Kernick D: A theoretical framework for multimorbidity: from complicated to chaotic. Br J Gen Pract 2012,:e659-e661.
74. King D Benbow SJ, Elizabeth J, Lye M: Attitudes of elderly patients to medical students. Med Educ 1992, 26:360-3.
75. Klinkman M, van Weel C: Prospects for person-centred diagnosis in general medicine. J Eval Clin Pract 2011, 17:365-370.
76. Lacroix A, Jacquemet S, Assal JP, Benroubi M: The patients' voice: testimonies from patients suffering from chronic disease. Pat Educ Counsel 1995, 26:293-9
77. Légaré F, Ratté S, Stacey D, Kryworuchko J, Gravel K, Graham ID, Turcotte S: Interventions for improving the adoption of shared decision making by healthcare professionals. Cochrane Database Syst Rev 2010, 12: CD006732.
78. Legare F, Stacey D, Graham ID, Elwyn G, Pluye P, Gagnon M, Frosch D, Harrison MB, Kryworuchko J, Pouliot S, Desroches S. Advancing theories, models and measurement for an interprofessional approach to shared decision making in primary care: a study protocol. BMC Health Serv Res 2008, 8:2.
79. Legare F, Turcotte S, Stacey D, Ratte S, Kryworuchko J, Graham ID. Patients' Perceptions of Sharing in Decisions A Systematic Review of Interventions to Enhance Shared Decision Making in Routine Clinical Practice. Patient 2012, 5:1-19.
80. Leykum LK, Palmer R, Lanham H, Jordan M, McDaniel RR, Noel PH, Parchman M: Reciprocal learning and chronic care model implementation in primary care: results from a new scale of learning in primary care. BMC Health Serv Res 2011, 11:44.
81. Loffler C, Kaduszkiewicz H, Stolzenbach C, Streich W, Fuchs A, van den Bussche H, Stolper F, Altiner A: Coping with multimorbidity in old age - a qualitative study. BMC Fam Pract 2012, 13:45.
82. Logan R: The teaching of community medicine in the undergraduate curriculum. Br J Med Educ 1969, 3:185-91.
83. Lugtenberg M, Zegers-van JM,JM, Westert GP, Burgers JS: Why don't physicians adhere to guideline recommendations in practice? An analysis of barriers among Dutch general practitioners. Implement Sci 2009, 4:54.
84. Luijks HD, Loeffen MJW, Lagro-Janssen AL, van Weel C, Lucassen PL, Schermer TR: GP's considerations in multimorbidity management: a qualitative study. Brit J Gen Pract 2012, 62:e504-e510
85. Mangin D, Heath I, Jamoulle M: Beyond diagnosis: rising to the multimorbidity challenge. BMJ 2012, 344:e3526-e3528.
86. Mann K, Holmes DB, Hayes VM, Burge FI, Viscount PW: Community family medicine teachers’ perceptions of their teaching role. Med Educ 2001, 35:278-85.
87. Marsteller JA, Hsu YJ, Reider L, Frey K, Wolff J, Boyd C, Leff B, Karm L, Scharfstein D, Boult C: Physician satisfaction with chronic care processes: a cluster-randomized trial of guided care. Ann Fam Med 2010, 8:308-15.
88. Martin C, Rohan BG: Chronic illness care as a balancing act. A qualitative study. Aust Fam Physic 2002, 31:55-9.
89. Morris R, Sanders C, Kennedy A: Shifting priorities in multimorbidity: a longitudinal qualitative study of patients' prioritization of multiple conditions. Chron Illness 2011,7:147.
90. Matthews C: Role modelling: how does it influence teaching in family medicine? Med Educ 2000, 34:443–8.
91. Mayor V: Long-term conditions. 3: Being an expert patient. Br J Comm Nurs 2006, 11:59-63.
92. Mercer SW, Jani BD, Maxwell M, Wong SY, Watt GC: Patient enablement requires physician empathy: a cross-sectional study of general practice consultations in areas of high and low socioeconomic deprivation in Scotland. BMC Fam Pract 2012, 13:6.
93. Mercer. Managing patients with mental and physical multimorbidity. BMJ 2012, 345:e5559.
94. Mishra SI, Gioia D, Childress S, Barnet B, Webster RL: Adherence to medication regimens among low-income patients with multiple comorbid chronic conditions. Health Soc Work 2011, 36:249-58.
95. Moth G: Chronic care management in Danish general practice - a cross-sectional study of workload and multimorbidity. BMC Fam Pract 2012, 13:52.
96. Muir F: Placing the patient at the core of teaching. Med Teach 2007, 29:258-60.
97. Munro N, Hornung R, McAleer S: What are the key attributes of a good general practice trainer: a Delphi study. Educ Gen Pract 1998, 9:263–70.
98. Nieman LZ, Cheng L: Chronic illness needs educated doctors: An innovative primary care training program for chronic illness education. Med Teach 2011; 33:e340-8.
99. NHS Improvement: Effective pathways for long term conditions. Leicester : NHS Improvement, 2012 Web publication. <http://www.improvement.nhs.uk/documents/LTC_Brochure.pdf>
100. Noel PH, Parchman ML, Williams JW, Cornell JE, Shuko L, Zeber JE, Kazis LE, Lee AFS, Pugh JA:The challenges of multimorbidity from the patient perspective. Journal of General Internal Medicine 2007, 22: 419-24.
101. O'Brien R, Wyke S, Guthrie B, Watt G, Mercer S: An 'endless struggle': A qualitative study of general practitioners' and practice nurses' experiences of managing multimorbidity in socio-economically deprived areas of Scotland. Chronic Illness, 2011 7:45-59.
102. O’Flynn N, Spencer J, Jones R: Does teaching during a general practice consultation affect patient care? Br J Gen Pract, 1999. 49:7-9
103. O'Flynn N. Improving the experience of care for people using NHS services: summary of NICE guidance. BMJ 2012, 344:d6422.
104. O’Sullivan M, Martin J, Murray E: Students’ perceptions of the relative advantages and disadvantages of community-based and hospital-based teaching: A qualitative study. Medical Education. 2000, 34:648–655.
105. Parboosingh IJ, Reed VA, Palmer JC, Bernstein HH: Enhancing Practice Improvement by Facilitating Practitioner Interactivity: New Roles for Providers of Continuing Medical Education. J Contin Educ Health Prof 2011, 31:122-127.
106. Pearson DJ, Lucas BJ: Engagement and opportunity in clinical learning: findings from a case study in primary care. Med Teach 2011, 33:670–7.
107. Peile EB, Easton GP, Johnson N: The year in a training practice: what has lasting value? Grounded theoretical categories and dimensions from a pilot study. Med Teach 2001, 23:205–11.
108. Phillips J. Patients can educate doctors about long term disease. BMJ 1999, 319:785.
109. Pichlhofer O, Tonies H, Spiegel W, Wilhelm-Mitteracker A, Maier M: Patient and preceptor attitudes towards teaching medical students in general practice. BMC Medical Education 2013, 13:33.
110. Rakel D: Creating expertise in health and healing. Journal of the American Board of Family Medicine. J Am Board Fam Med 2007, 20:611.
111. Roland M, Paddison, C: Better management of patients with multimorbidity. BMJ 2013, 346:f2510.
112. Russell G, Thille P, Hogg W, Lemelin J: Beyond fighting fires and chasing tails? Chronic illness care plans in Ontario, Canada. Ann Fam Med 2008, 6:146-153.
113. Salisbury C. Multimorbidity: redesigning health care for people who use it. Lancet 2012, 380:7-9.
114. Sagasser MH, Kramer AWM, van der Vleuten CPM: How do postgraduate GP trainees regulate their learning and what helps and hinders them? A qualitative study. BMC Med Educ 2012, 12: 67.
115. Schers H, Webster S, van den Hoogen H, Avery A, Grol R, van den Bosch W: Continuity of care in general practice: a survey of patients’ views. Br J Gen Pract 2002, 52: 459-62.
116. Schneider A, Korner T, Mehring M, Wensing M, Elwyn G, Szecsenyi J: Impact of age, health locus of control and psychological co-morbidity on patients' preferences for shared decision making in general practice. Pat Educ Counsel 2006, 61:292-8.
117. Schuez B, Wurm S, Warner LM, Ziegelmann JP: Self-efficacy and multiple illness representations in older adults: A multilevel approach. Psychol Health 2012, 27:13-29.
118. Schuling J, Gebben H, Veehof LJG, Haaijer-Ruskamp FM: Deprescribing medication in very elderly patients with multimorbidity: the view of Dutch GPs. A qualitative study. BMC Fam Pract 2012, 13:56-61.
119. Scott J, Tallia A, Crosson JC, Orazano AJ, Stroebel C, DiCicco-Bloom B, O’Malley D, Crabtree B: Social network analysis as an analytic tool for interaction patterns in primary care practices. Ann Fam Med. 2005, 3:443-448.
120. Sherbourne CD, Sturm R, Wells KB: What outcomes matter to patients? J Gen Intern Med 1999, 14:357-66.
121. Smith CS, Irby DM: The roles of experience and reflection in ambulatory care education. Acad Med 1997, 72:32–5.
122. Smith CS, Morris M, Francovich C, Hill W, Gieselman J: A qualitative study of resident learning in the ambulatory clinic. The importance of exposure to ‘breakdown’ in settings that support effective response. Adv Health Sci Educ 2004, 9:93–105.
123. Smith SM, O'Kelly S, O'Dowd T: GPs' and pharmacists' experiences of managing multimorbidity a 'Pandora's box'. Br J Gen Pract 2010, 60:501-503.
124. Smith SM, Soubhi H, Fortin M, Hudon C, O'Dowd T: Interventions for improving outcomes in patients with multimorbidity in primary care and community settings. Cochrane Database Syst Rev 2012, CD006560.
125. Smith SM: Managing patients with Multimorbidity: systematic review of interventions in primary care and community settings. BMJ 2012, 345:e5205.
126. Soubhi H, Bayliss EA, Fontin M, Hudon C, van der Akker M, Thivierge R, Posel N, Fleiszer D: Learning and caring in communities of practice: using relationships and collective learning to improve primary care for patients with multimorbidity. Annals Fam Med 2010, 8:170-177.
127. Soubhi H: Toward an ecosystemic approach to chronic care design and practice in primary care. Annals Fam Med 2007, 5:263-9.
128. Stewart JHP: Learning from the learners: what do trainees want from general practice vocational training? Asia Pac Fam Med 2002, 1:28–32.
129. Svenberg K: A memorable consultation: Writing reflective accounts articulates students’ learning in general practice. Scand J of Prim Health Care 2007, 25:75–79.
130. Swanick T, Plint S: From supernumerary to supervised professional development: work-based learning for specialist training for general practice. Educ Prim Care 2006, 17 :97–103.
131. Tinetti ME, Fried TR, Boyd CM: Designing health care for the most common chronic condition – Multimorbidity. J Am Med Assoc 2012, 307:2493-2494.
132. Townsend A. Applying Bourdieu's theory to accounts of living with multimorbidity. Chron Illness 2012, 8:89-101.
133. Treasure W: First do no harm:education deals with the application of general principles in uncertain situations. Br J Gen Pract 2013, 63:151.
134. van der Zwet J, Zwietering PJ, Teunissen P, van der Vleuten CPM, Scherpbier, AJ J A: Workplace learning from a socio-cultural perspective: creating developmental space during the general practice clerkship. Adv Health Sci Educ 2011, 16:359-73.
135. van Dijk-de Vries A, Moser A, Mertens V, van der Linden J, van der Weijden T, Th. M van Eijk, J: The ideal of biopsychosocial chronic care: how o make it real? A qualitative study among Dutch stakeholders. BMC Fam Pract 2012, 13:14.
136. van Walraven C, Oake N, Jennings A, Forster AJ: The association between continuity of care and outcomes: a systematic and critical review. J Eval Clin Pract 2010, 16:947-56.
137. Wagner EH, Austin BT, Davis C, Hindmarsh M, Schaefer J, Bonomi A: Improving chronic illness care: translating evidence into action. Health Affairs 2001, 20:64-78.
138. Wearne S, Dornan T, Teunissen PW, Skinner T: General practitioners as supervisors in postgraduate clinical education: an integrative review. Med Educ 2012, 46:1161-73.
139. Wehling, M: Guideline-Driven Polypharmacy in Elderly, Multimorbid Patients is Basically Flawed: there are almost no Guidelines for these Patients. J Am Geriatr Soc 2011, 59:376-376.
140. Yardley S, Teunissen PW, Dornan T: Experiential learning: AMEE Guide No. 63. Med Teach. 2012; 34:e102-15
141. Zweig S, Williamson HA, Jr: Adverse effects of faculty practice on diagnostic content of residents' outpatient experience. J Fam Pract 1987, 25:491-6.
