# Supplementary material for: Modelling successful primary care for multimorbidity: a realist synthesis of successes and failures in concurrent learning and healthcare delivery
Source: BMC Fam Pract. 2015 Feb 25;16:23. doi: 10.1186/s12875-015-0234-9 (PMC4343192; doi:10.1186/s12875-015-0234-9)
Supplement: Additional file 4: — Data extraction sheet v1.0. [file 12875_2015_234_MOESM4_ESM.docx]

| **FULL ARTICLE REFERENCE** | |  | | | |
| --- | --- | --- | --- | --- | --- |
| **SUMMARY** | |  | | | |
| **If clear rejection on reading full article give justification here** | |  | | | |
| **ITEM** | | **EXTRACTED ELEMENTS** | | **DETAILED INFORMATION** | **QUALITY NOTES ETC – E.G. RELEVANCE TO RQ** |
| **CONTEXT** | *STUDY DESIGN OR ARTICLE CATEGORY* |  | |  |  |
|  | *TRAJECTORY OF ILLNESS/SPECIFIC DISEASES/CO/MULTIMORBIDITY* | *POPULATION DEMOGRAPHICS* | |  |  |
|  | *CONTINUUM OF EDUCATION* | *POPULATION DEMOGRAPHICS* | |  |  |
|  | *PERSPECTIVES* | *PRACTITIONERS* | |  |  |
|  |  | *LEARNERS* | |  |  |
|  |  | *PATIENTS* | |  |  |
|  |  | *POLICY MAKERS/COMMISSIONERS* | |  |  |
|  |  | *COMMUNITY/PUBLIC* | |  |  |
|  |  | *OTHERS* | |  |  |
|  | *DRIVERS FOR INTERVENTION* | *POLICY* | |  |  |
|  |  | *OTHERS* | |  |  |
| **MECHANISMS** | *INTERVENTION IN PRACTICE* | *HEALTH SERVICE DELIVERY ACTIVITIES AND GOALS* | |  |  |
|  |  | *WORKPLACE BASED EDUCATION (ExBL) ACTIVITIES AND GOALS* | |  |  |
|  |  | *MODEL/THEORY USED FOR SERVICE DELIVERY* | |  |  |
|  |  | *MODEL/THEORY USED FOR EDUCATION* | |  |  |
|  |  | *DEVELOPMENTAL SPACE (ZPD, Z ACTUAL PRACTICE?)* | |  |  |
|  | *IDENTIFIABLE REAL WORLD WORKING PRACTICES* | *FOCUS OF LEARNING (E.G AUTHENTIC PATIENTS)* | |  |  |
|  |  | *FOCUS OF SERVICE* | |  |  |
|  |  | *ALTERATIONS FROM POLICY IN IMPLEMENTATION* | |  |  |
|  |  | *THEORY-PRACTICE GAPS* | |  |  |
|  |  | *WHOLENESS* OF CARE PROCESS* | |  |  |
|  |  | *WHOLENESS OF EDUCATIONAL PROCESS* | |  |  |
|  | *SOCIAL PROCESSES AND INTERACTIONS* | *AGENCY* | |  |  |
|  |  | *STRUCTURES* | |  |  |
|  |  | *INTERACTIVITY* | |  |  |
|  |  | *NEGOTIATION* | |  |  |
|  |  | *TABOOS* | |  |  |
|  |  | *ROLES AND IDENTITY* | |  |  |
|  |  | *ARTEFACTS* | |  |  |
|  | *CONSTRUCTION OF INTERACTIONS* | *GENERAL ATTITUDES* | |  |  |
|  |  | *METAPHORS* | |  |  |
|  |  | *CAUSAL STATEMENTS/CLAIMS* | |  |  |
|  |  | *NARRATIVES* | |  |  |
|  |  | *CONSTRUCTION OF SUCESS AND FAILURE IN ABSENCE OF CURE* | |  |  |
|  |  | *RISK, RESPONSIBLITY, TRUST* | |  |  |
|  |  | *CONSTRUCTION OF LIVING WITH SYMPTOMATIC CHRONIC ILLNESS* | |  |  |
|  |  | *CONSTRUCTION OF LIVING WITH DYING* | |  |  |
| **OUTCOMES** | *PRACTICE* | *PROVISION OF APPROPRIATE INDIVIDUALISED HEALTHCARE* | |  |  |
|  |  | *PROVISION OF CONSTRUCTIVE WORKPLACE-BASED LEARNING FOR FUTURE PROFESSIONALS* | |  |  |
|  |  | *EVIDENCE OF SUSTAINABILITY* | |  |  |
|  | *OTHER CONSEQUENCES* | *CONSTRUCTIVE* | |  |  |
|  |  | *DESTRUCTIVE* | |  |  |
| **OVERALL QUALITY ASSESSMENT** | | | | | |
| *RELEVENCE TO RESEARCH QUESTION – does the research address the theory under test?* | | | |  | |
| *RIGOUR – does the research support the conclusions drawn from it?* | | | |  | |
| **SUMMARY NOTES FOR KEY RESEARCH QUESTIONS** | | | | | |
| *‘what works for whom, to what extent, in what circumstances, in what respect, how and why?’* | | |  | | |
| *How and why are success and failure in medical activities conceptualised in the absence of cure?* | | |  | | |
| *How and why does learning about and delivering healthcare in primary care for people with co/multimorbidity work?* | | |  | | |
| *How do interactions between agents and structures at individual, local and wider institutional levels affect consequences?* | | |  | | |
| *How do official explanations compare to actual practice of both education and service delivery?* | | |  | | |

*WHOLENESS: PATIENT CARE AND LEARNING TASKS SHOULD BE ‘END TO END’ COMMENCING WITH PATIENT PROBLEM AND ENDING WITH ACTION PLAN FOR PATIENT

Please select any specific words or sentences/text from the article to quote here under each category listed in the left hand column. For example describing patients as ‘challenging’ would be considered as a potential ‘failure’ of care delivery while ‘improved care’ would be a conceptualisation of success (even if what it means is not defined)

| **Category** | **Quotes/selected words** |
| --- | --- |
| Conceptualisations of failure in the absence of cure |  |
| Conceptualisations of success in the absence of cure |  |
| Failure words |  |
| Metaphors for failure in the absence of cure |  |
| Metaphors for success in the absence of cure |  |
| Success words |  |

| Citations identified for round 3 follow up | Outcome of checking |
| --- | --- |
|  |  |
|  |  |
|  |  |
|  |  |
|  |  |
|  |  |
|  |  |
