# Supplementary material for: Modelling successful primary care for multimorbidity: a realist synthesis of successes and failures in concurrent learning and healthcare delivery
Source: BMC Fam Pract. 2015 Feb 25;16:23. doi: 10.1186/s12875-015-0234-9 (PMC4343192; doi:10.1186/s12875-015-0234-9)
Supplement: Additional file 5: — Data extraction sheet v2.0. [file 12875_2015_234_MOESM5_ESM.docx]

| **FULL ARTICLE REFERENCE** | |  | |
| --- | --- | --- | --- |
| **SUMMARY** | |  | |
| **If clear rejection on reading full article give justification here** | |  | |
| **ITEM** | | **EXTRACTED ELEMENTS** | **DETAILED INFORMATION** |
| **CONTEXT** | *STUDY DESIGN OR ARTICLE CATEGORY* |  |  |
|  | *TRAJECTORY OF ILLNESS/SPECIFIC DISEASES/CO/MULTIMORBIDITY* | *POPULATION DEMOGRAPHICS* |  |
|  | *CONTINUUM OF EDUCATION* | *POPULATION DEMOGRAPHICS* |  |
|  | *PERSPECTIVES* | *PRACTITIONERS* |  |
|  |  | *LEARNERS* |  |
|  |  | *PATIENTS* |  |
|  |  | *POLICY MAKERS/COMMISSIONERS* |  |
|  |  | *COMMUNITY/PUBLIC* |  |
|  |  | *OTHERS* |  |
|  | *DRIVERS FOR INTERVENTION* | *POLICY* |  |
|  |  | *OTHERS* |  |
|  |  |  |  |
| **OVERALL QUALITY ASSESSMENT** | | | |
| *RELEVENCE TO RESEARCH QUESTION – does the research address the theory under test?* | | |  |
| *RIGOUR – does the research support the conclusions drawn from it?*  *Is any intervention sustainable? – resources, and future professionals being equipped alongside current professionals delivering care* | | |  |

| **SUMMARY NOTES FOR KEY RESEARCH QUESTIONS*** | |
| --- | --- |
| *‘what works for whom, to what extent, in what circumstances, in what respect, how and why?’*  *(models, wholeness of care / education process, provision of individualised care / education)* |  |
| *How and why are success and failure in medical activities conceptualised in the absence of cure?* |  |
| *How and why does learning about and delivering healthcare in primary care for people with co/multimorbidity work?*  *(focus of learning, learning outcomes/consequences)* |  |
| *How do interactions between agents and structures at individual, local and wider institutional levels affect consequences?*  *(living with chronic illness, living with dying)* |  |
| *How do official explanations compare to actual practice of both education and service delivery?*  *(theory-practice gaps)* |  |
| *Does this paper contain a diagrammatic model which might be useful in thinking about our key research questions or how concurrent education and healthcare delivery happens in practice* | Y/N – if yes please copy and paste at the end of this coding sheet |

*WHOLENESS: PATIENT CARE AND LEARNING TASKS SHOULD BE ‘END TO END’ COMMENCING WITH PATIENT PROBLEM AND ENDING WITH ACTION PLAN FOR PATIENT

| Citations identified for round 3 follow up | Outcome of checking |
| --- | --- |
|  |  |
|  |  |
|  |  |
|  |  |
|  |  |
|  |  |
|  |  |
